# Practical immunomodulatory landscape of glioblastoma multiforme (GBM) therapy

**DOI:** 10.1186/s43046-024-00240-4

**Published:** 2024-10-28

**Authors:** Seyedeh Elham Norollahi, Bahman Yousefi, Fatemeh Nejatifar, Shahrokh Yousefzadeh-Chabok, Ali Rashidy-pour, Ali Akbar Samadani

**Affiliations:** 1 Cancer Research Center and, Department of Immunology, Semnan University of Medical Sciences, Semnan, Iran; 2 Department of Hematology and Oncology, School of Medicine, Razi Hospital, Guilan University of Medical Sciences, Rasht, Iran; 3 Guilan Road Trauma Research Center, Trauma Institute, Guilan University of Medical Sciences, Rasht, Iran; 4 Rasht, Iran; 5 Research Center of Physiology, Semnan University of Medical Sciences, Semnan, Iran

**Keywords:** Glioblastoma multiforme, Signaling pathways, Immunotherapy, Translational element

## Abstract

Glioblastoma multiforme (GBM) is the most common harmful high-grade brain tumor with high mortality and low survival rate. Importantly, besides routine diagnostic and therapeutic methods, modern and useful practical techniques are urgently needed for this serious malignancy. Correspondingly, the translational medicine focusing on genetic and epigenetic profiles of glioblastoma, as well as the immune framework and brain microenvironment, based on these challenging findings, indicates that key clinical interventions include immunotherapy, such as immunoassay, oncolytic viral therapy, and chimeric antigen receptor T (CAR T) cell therapy, which are of great importance in both diagnosis and therapy. Relatively, vaccine therapy reflects the untapped confidence to enhance GBM outcomes. Ongoing advances in immunotherapy, which utilizes different methods to regenerate or modify the resistant body for cancer therapy, have revealed serious results with many different problems and difficulties for patients. Safe checkpoint inhibitors, adoptive cellular treatment, cellular and peptide antibodies, and other innovations give researchers an endless cluster of instruments to plan profoundly in personalized medicine and the potential for combination techniques. In this way, antibodies that block immune checkpoints, particularly those that target the program death 1 (PD-1)/PD-1 (PD-L1) ligand pathway, have improved prognosis in a wide range of diseases. However, its use in combination with chemotherapy, radiation therapy, or monotherapy is ineffective in treating GBM. The purpose of this review is to provide an up-to-date overview of the translational elements concentrating on the immunotherapeutic field of GBM alongside describing the molecular mechanism involved in GBM and related signaling pathways, presenting both historical perspectives and future directions underlying basic and clinical practice.

**Graphical Abstract:**

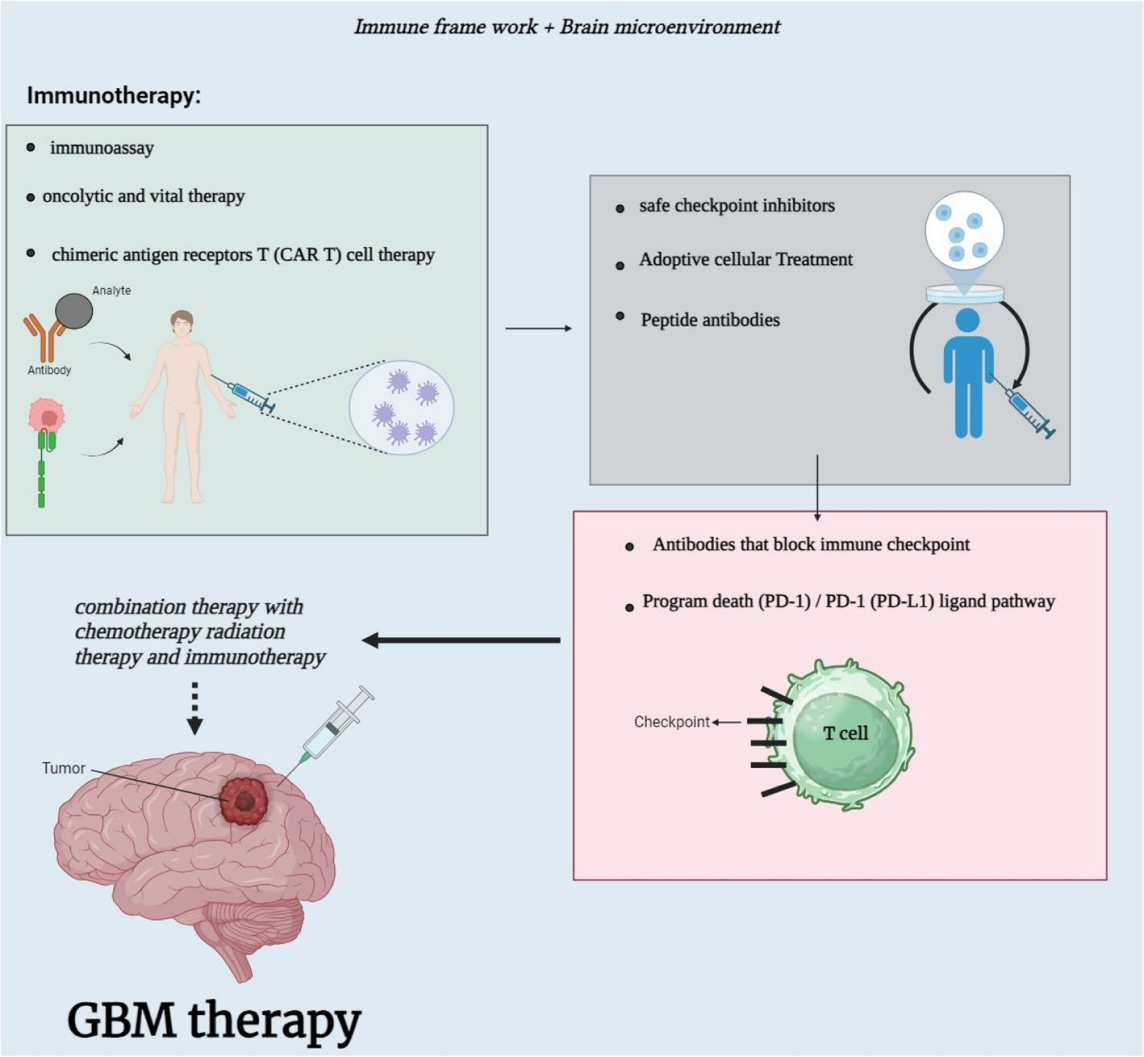

## Introduction

The most common and deadly primary brain tumor among adult patients is glioblastoma (GBM) [1]. With notable variations between patients, GBM is a physiologically diverse tumor that demonstrates all of the traditional characteristics of malignancy [2]. Notably, the gold standard of treatment is combined radio-chemo- and tumor-treating field therapy [3, 4], which increases the mean overall survival of patients to 21 months [4]. Although prognostically significant, GBM subtypes have been identified by genetic [5] and epigenetic [6] approaches and personalized treatments that target certain pathogenic processes or molecular targets that have not yet been developed. Specifically, designed cells are expected to coordinate invasion along pre-existing central nervous system (CNS) structures such as blood arteries, subarachnoid space, and white matter tracts [7], which may result in a collective invasion [8, 9]. GBM cells can infiltrate as single cells [10] or as a group [11, 12] by modifying their cellular skeleton and extracellular matrix [7]. Local and distant bulky relapses may be brought on by distant GBM cells’ reinvasion into the main tumor position and the invasion of distant tissues. Many early tumor mutations are shared by recurring tumors and their offspring [13], recommending that phylogenetic progenitor clones of primary tumors live in habitats where they can emerge from their latent state and proliferate locally [14]. Numerous invasion patterns have been identified [15, 16], and they all rely on interactions with the microenvironment [8] and genetic programs [17]. GBM brain tumor-initiating cells (BTIC) and differentiated cells can be used to imitate invasion [18–20]. Anyway, the involved cellular and molecular elements in GBM are of great importance and can help researchers strongly to find a better diagnostic and therapeutic method.

### Immune structure of GBM

**Fig. 1:**
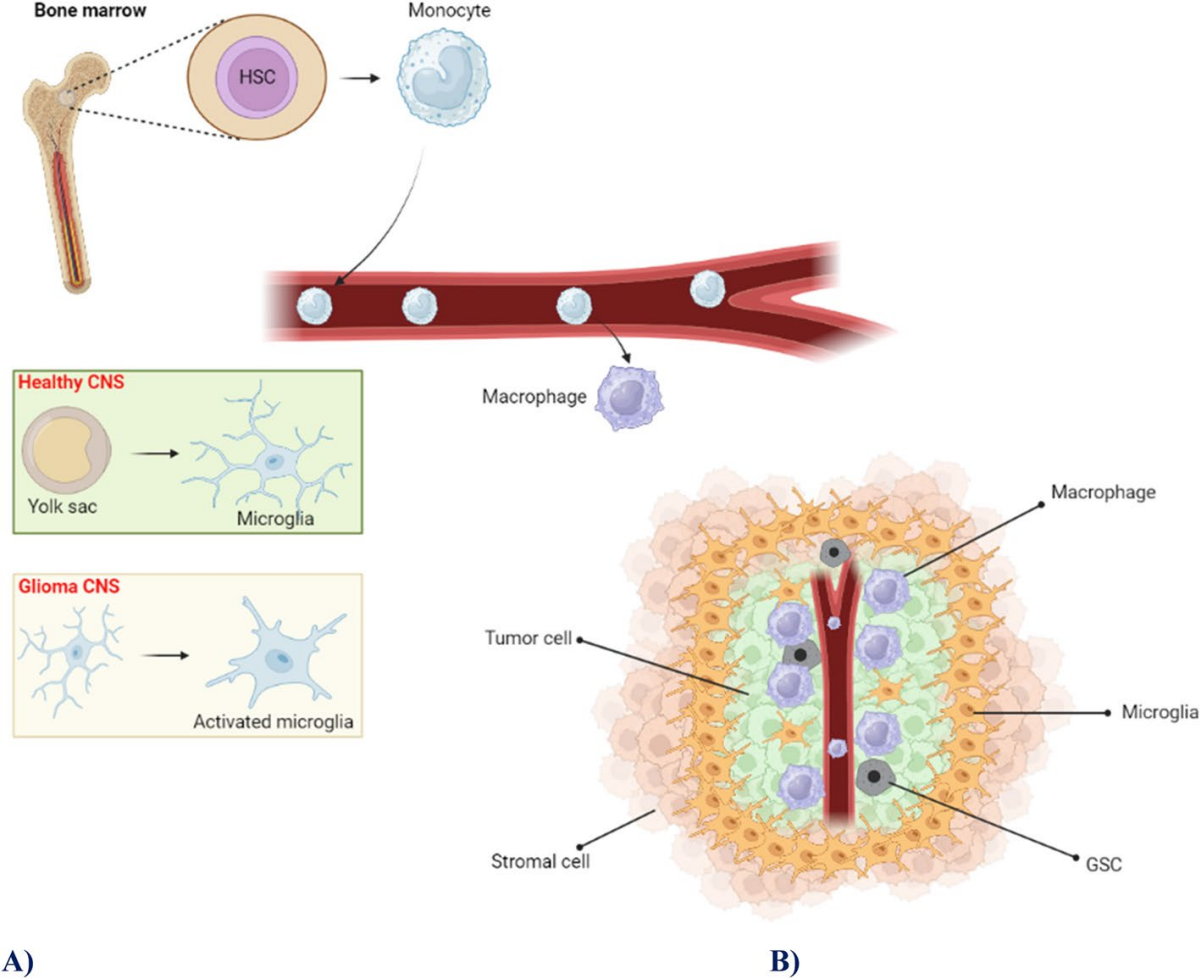
Tumor-related macrophages (TAM) in GBM. **A** TAMs originated from two eclectic originations. The brain is full of bone microglia or marrow-derived monocytes. **B** In the proneural GBM cellular population, the major number of TAMs are BMDMs, which are chiefly, located in the perivascular niches which are also a niche for glioma stem cells (GSCs). Most microglia occur in the peritumoral area

Various considerations in the use of quality expression from the Omnibus database Quality Expression and The Cancer Genome Atlas (TCGA) have shown that qualitatively rich expression is associated with immune responses, particularly of the tumor-associated macrophage (TAM) genes, in the mesenchymal (MES) subtype of GBM compared with other subtypes [21], most likely indicating that TAM has a specific sub-role in terms of GBM. Indeed, TAMs have a major role in tumor development. However, several correlated studies suggest that TAMs may perform different functions in GBM subtypes. In contrast, despite increasing evidence from animal models and TCGA analyses of human glioblastoma (hGBM) [22], the clinical value of these data remains unclear, as neurofibromatosis type 1 (NF1) deficiency increases TAM infiltration. Although a growing body of preclinical data proposes that disease-specific therapies may be of preferred patient benefit, these subtypes have not been clinically proven to be biomarkers to predict survival rates [5]. However, it remains unclear what governs the variations in the immunological composition of the GBM subtypes. One possibility may be that genetically modified tumor-associated or tumor-specific antigens exist in distinct subtypes that influence different molecular immune responses and underlie differences [5, 23]. The inflammatory and proangiogenic microenvironment that is produced by glioblastoma increases adhesion molecule expression and decreases tight junctions in endothelial cells, which in turn increases blood–brain barrier (BBB) permeability. These changes allow leukocytes to exit the bloodstream through extravasation through the brain’s endothelial wall and infiltration of tumor masses. In addition to TAMs, many additional immune cells could be identified in GBM parenchyma, albeit at a much lower frequency. T cells certainly make up the majority of lymphocytes in GBM, but according to flow cytometric analysis, their frequency is less than 0.35% of cells isolated from hGBM tumor tissue biopsy samples. Despite being an important cytoprotective agent in tumor cell elimination, CD8 + cytotoxic T cells are only sporadic present in GBM parenchyma and account for less than 35% of total CD3 + T cells [24]. The sensitivity of T cells isolated from GBM patients is lower than T cells from healthy controls sensitive to direct in vitro anti-CD3 activation, which indicates an immunosuppressed condition [24]. Recent research has demonstrated an association between immune-inhibitory receptor indoleamine 2,3-dioxygenase 1 (IDO1) levels, which is expressed more frequently by T cells that have infiltrated a GBM, with poor prognosis of the disease [25, 26]. Regulatory T cells (Tregs) could also be identified in GBM parenchyma and are thought to have immunosuppressive functions and inhibit antitumor immunity in different solid tumors like breast, ovarian, and pancreas cancers [27]. A phase I clinical trial investigating the utility and safety of an IDO1 inhibitor combined with temozolomide (TMZ) in children with primary malignant brain tumors is currently underway [28, 29]. In vitro, T cell activity in GBM patients is restored to levels comparable to healthy controls after Treg depletion removes the T cell proliferative defect [30]. Therefore, targeting Tregs may reverse tumor immune evasion and contribute to conventional or tumor immunotherapy. An in silico investigation of 22 human NP immune cell types confirmed and indicated collective increases in multiple cell types, including memory T cells (CD4 +), neutrophils, and polarized type 2 macrophages in cell MES tumors compared with non-MES tumors and classic (CL) and MES samples [22]. Theoretically, the immunosuppressive properties of TAM could block the effector T cell infiltration at higher concentrations. However, the etiology of the direct invasion of TAMs and T cells specific to this subtype is not immediately clear. This may be because T cells leave the blood stream passively following TAMs secondary to BBB damage during GBM development. However, this is unlikely because the T cell-to-TAM ratio in the tumor differs from that in the blood, and the number of lymphocytes is higher than monocytes. One possible explanation is that the chemokines chemokines C–X–C motif ligand (CXCL) and C–C motif ligand (CCL) secreted by MES tumors attract T or TAM cells, respectively, in tandem with other subtypes of GBM. Transgenic mouse models (GEMMs) can have enhanced, stable summary hGBM subtypes, providing an important tool for studying subtype-specific and related immunopathology development of effective therapies [31, 32]. These individual GEMMs make an unprecedented opportunity to identify the molecular signaling and immune cells that contribute to glioma formation and their continued proliferation driven by the microenvironment subtype-specific glioma. GEMM with different GBM subtypes is a better choice than other models for specific questions about interactions between the tumor and its microenvironment. Mouse orthologous allograft employing murine GBM cell lines which have been cultured for many years in serum or species-incompatible hGBM xenografts, especially those that are incompatible with chemokine and receptor them, indicated its significance. One of the desirable features of these biological models is the use of immunocompetent mice, where immune and tumor cells belong to the same species. This may eliminate species-specific interactions between cytokines, chemokines, and their receptors, which are essential for differential immune mobilization and cell-type incompatibility. The GEMM model of GBM can be used to answer critical biological questions on the relevance of differential immune cell infiltration in different subtypes of hGBM. Several additional studies later showed that blood-derived myeloid progenitor cells in mice did not contribute to postnatal adult microglia significantly. Thus, the major number of adult microglia arise from the yolk sac, maintained by longevity, and have low self-renewal capacity [33–35]. By monitoring the lifespan of microglia with long-term imaging from a single cell in mice model, it has been demonstrated that resident neural microglia have an average lifespan of 15 months, which is roughly equal to the lifespan of other microglia. Although the naive parenchyma of CNS is dominated by resident microglia, different condition was identified in the tumor-bearing CNS. In tumor-bearing brains, the BBB is damaged and mononuclear chemoattractant protein (MCP) family expression is increased. This allows monocytes to enter the tumor from the peripheral border and then differentiate into macrophages. Hematopoietic stem cell-derived DC macrophage progenitor cells are the progeny that form monocytes. These progenitor cells differentiate into monocytes in the bone marrow, then released into the bloodstream to invade peripheral organs [36]. Mouse monocytes could be divided into two main cellular populations, CX3CR1int, Ly6C + , CCR2 + inflammatory monocytes, and Ly6C − , CX3CR1hi as well as circulating monocytes CCR2 − [37]. It is well known that Ly6C + , CX3CR1int, and CCR2 + inflammatory monocytes leave the bloodstream and migrate to inflamed tissues. Once these cells reach the inflamed tissue, they differentiate into macrophages as they gradually upregulate CCR2 and simultaneously upregulate CX3CR1 [38]. Interestingly, TAMs express multiple levels of CCR2 and CX3CR1 in a reciprocal new model, suggesting that these cells are continuously transformed from infiltrated monocytes to mature macrophages [39]. This dynamic surface molecule switching suggests that myeloid-derived monocytes have high plasticity and that they mature after localization in the tumor [40]. It has been found that bone marrow-derived microglia and macrophages respond differently to different types of CNS injury and may have different functions [41, 42]. A recent example applying the complex parabiosis model shows that peripheral mononuclear cells infiltrate the inflamed CNS in an experimental autoimmune encephalomyelitis model and play an important role in the process of progression to paralysis [43]. Bone marrow-derived cells were found in the perivascular region, while resident glial cells were more strongly expressed in the peritumoral region (Fig. 1). RNA-seq analysis revealed that bone marrow-derived TAMs and microglia-derived TAMs mainly shared this gene involved in “cell migration,” while genes involved in “inflammatory cytokines” and “transformation” are related. It became clear that there is an adjusted upward [39]. These differences can be partly explained by the difference between progenitor cells that these two cell populations are derived from and differences in transcription factors that they selectively use for gene regulation [44]. These results indicate that Cx3cr1 deletion indirectly promotes the transport of inflammatory monocytes to the central nervous system and leads to increased accumulation in perivascular regions [45]. However, there was no direct effect on microglial accumulation in the peritumoral region. Bone marrow-derived monocytes promote glioma stem cell proliferation through IL-1β production [45]. These novel findings recommend that bone marrow compartment-derived TAMs promote glioma formation, whereas microglia have not a central role in tumor growth and they appear to be mainly involved in tumor cell invasion.

### Role of immunosuppression in GBM

Macrophages that reside in tumors are mainly considered important promoters of tumor growth due to their pro-angiogenic and immunosuppressive effects. These cells include myeloid-derived suppressor cells (MDSCs). Interestingly, murine MDSCs are the main cells that can express both Gr1 and CD11b markers and can differentiate into granulocyte subsets and monocytes. Importantly, in GBM, a few granulocytic MDSCs can occur within the tumor microenvironment [39]. Monocytic MDSCs can yield many different mechanisms to suppress cell-mediated immune action, including upregulating Arg1 product, promoting the expansion of Treg populations, and/or T cell apoptosis [46]. Considerably, most of these features are permanent in the macrophages with M2 phenotype. Previous in vitro studies suggested that myeloid monocytes may dichotomize into a standard active pro-inflammatory phenotype and also a main anti-inflammatory phenotype [47]. Remarkably, M1 cells generate many inflammatory cytokines and oxidative metabolites that are necessary for host defense, but they can hurt all different tissues [48]. Menacingly, M2 cells improve the healing of the wound and suppress the responses of the unwanted immune system [49]. However, according to these impressive findings from practical experiments in cell culture, the absolute dichotomous dispensation of M1 and M2 is not commonplace in vivo environment [50]. Indeed, bone marrow-derived macrophage and tumor-associated microglia transcriptional investigation indicated mixed structures of M1 with M2 phenotypes in all TAM colonies. For instance, the canonical M2 marker arginase 1 had an upregulation level in both microglia and bone marrow-derived macrophages, and notably, the special M1 cytokine IL-1β had an upregulation level in all cells. Anyway, it is not evident whether these M1 and M2 features belong to diverse colonies or whether single cells can be able to express two molecular subsets at diverse levels. Transparently, TAMs have high plasticity and have been indicated to switch between two M1 and M2 phenotypes responding to foreign environmental stimulators [51]. There are several endeavors to polarize the TAMs to the fate of M1. Anyhow, there are major challenges as soluble parameters yielded by tumor cells may revert TAMs to the M2 phenotype despite translational medicine.

### Main signaling pathways in GBM

#### Notch signaling in GBM

One of the main signaling pathways in GBM is Notch signaling. Noteworthy, the Notch signaling pathway has a crucial responsibility in the regulation of many eclectic molecular, developmental, and cellular functions comprising number determination apoptosis, differentiation, neurogenesis, self-renewal, homeostasis, cell migration, and stem cell maintenance [52–54]. These main elements are strongly and widely spread in the brain cells, and Notch 1 is also expressed in astrocytes, neurons, epithelial and endothelial cells, and progenitor cells [55–57]. Interestingly, Notch 2 and 3 molecules are expressed strongly in progenitor cells [55–58], and in this way, the Notch 4 molecule is expressed in the endothelial cells [59]. DII1 is shown to be expressed in all parts of intermediate neuron progenitors and neuron cells [57, 58, 60, 61]. DII3 is expressed also in approximately all sections of intermediate neuron precursors [60]. In addition, DII4 is also expressed in the endothelial cells [62]. It is remarkable to say that many cells express Jagged 1 including neurons, progenitors, and intermediate neural progenitors [56, 57, 60, 63–65], and alongside this, the only cell that could express Jagged 2 is a neuron [57, 59]. The Notch pathway is operated by many different factors comprising metabolic interactions of Notch receptors and neighboring cellular ligands and suppressed by cis interactions through binding to the cell [66–68]. Activation of the Notch signaling pathway results in the expression of proteins included in the diagnosis of lineage. Dysregulation in the Notch signaling pathway is related to several types of cancers including colon, pancreatic, brain tumors, skin, breast, cervical, and blood [69–72]. Several translational researches about GBM confirmed that Notch receptors including Notch 1, 3, and 4, or their molecular components such as Hey1 and DII1 have irregular and unusual expression in brain tumors [73–75]. The high expression of Notch receptors 1 and 4 or other elements of Notch signaling including DII1, DII4, Hey1, Hey2, Jagged 1, and Hes1 have been confirmed and reported in many different studies [73, 76, 77]. Notch receptor 4 is also related to primary GBM with high grades. Anyhow, many studies have indicated low expression levels of Notch proteins including Notch 1 and Notch 2 in GBM [78]. The association between GBM and expression of Notch molecule has been evaluated in different cells like mesenchymal [79], classical [5], and nervous [5, 80]. So far, there are not sufficient studies examining the epigenetic performance of the Notch signaling pathway in GBM [81, 82]. Hey1 methylation status is thought to mediate the pathogenesis of GBM, and past research suggested it as a predictive marker for GBM patients [83]. Moreover, in the xenograft cell lines 4910 and 5310, treatment with inhibitors of histone deacetylase (HDAC), comprising sodium butyrate, mediated apoptosis of GBM cells, downregulating Hey1 expression and reducing DNA (cytosine-5) expression. In this regard, cerebellar growth and neurodevelopmental interaction between glial cells and Bergmann cells are regulated by the delta/Notch-like epidermal growth factor receptor (DNER), which results in the expression of Purkinje cells in a delta-dependent manner [84]. Also, in GBM-derived neurons, deletion of HDAC can activate the DNER/Deltex signaling, resulting in the inhibition of cell differentiation and neuronal growth [84]. However, more studies are needed to investigate the epigenetic role of Notch signaling in GBM and find potential therapeutic targets.

#### Hedgehog signaling in GBM

**Fig. 2:**
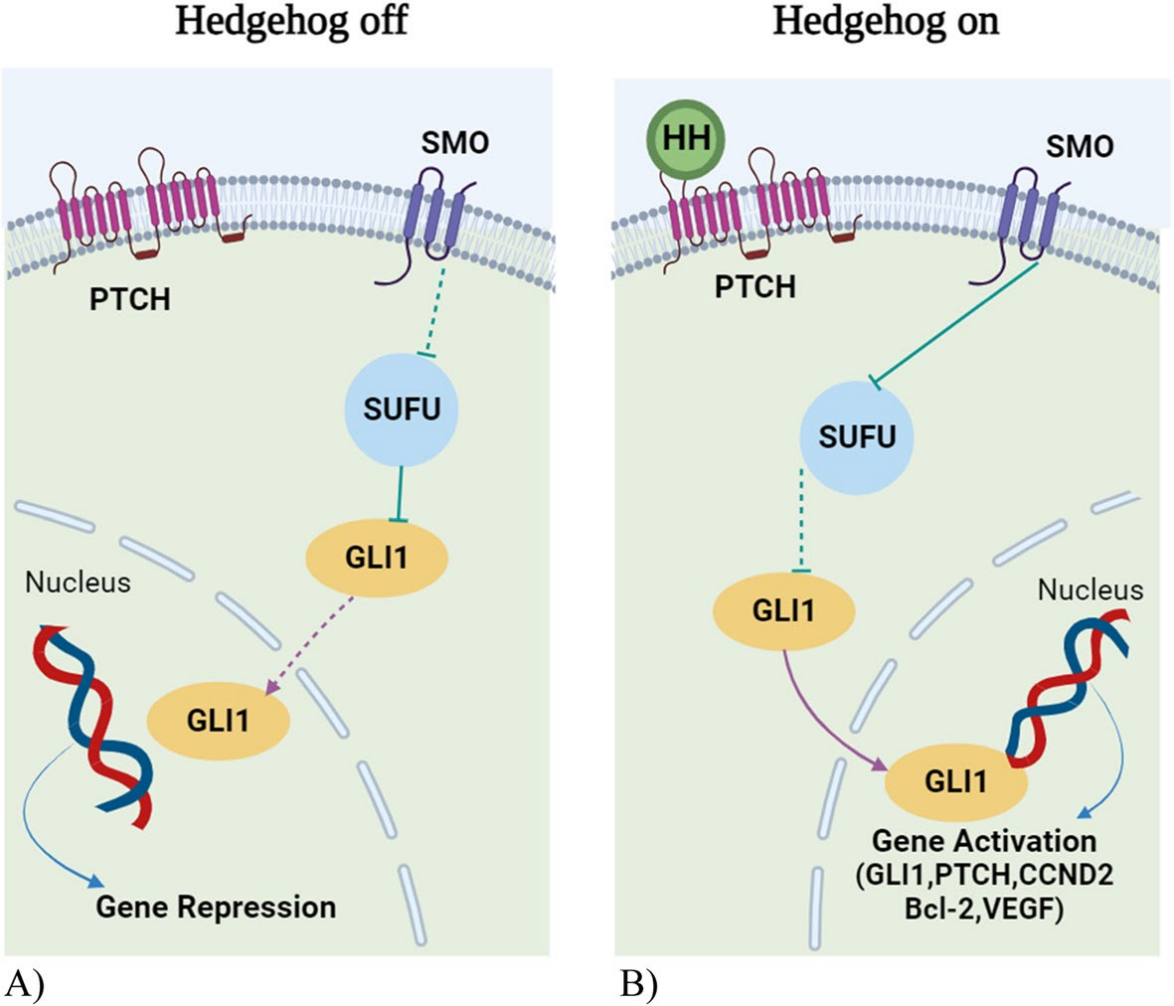
Hedgehog signaling pathway. **A** Hedgehog signaling pathway inactivation in the absence of a Hedgehog ligand. **B** The binding of the Hedgehog to the patched receptor (PTCH) revitalizes the hedgehog signaling pathway. SMO, smoothing receptor; SUFU, suppressor

Hedgehog (HH) signaling has an important role in embryogenesis as well as tumorigenesis [85, 86]. Regulation of cellular proliferation and differentiation can be an important pathway for embryonic patterning [87, 88]. Regulation of tissue repair, stem cell maintenance, and regeneration can play an important role in HH signaling after puberty [86]. Congenital malformations such as holoprosencephaly and microcephaly are the result of dysregulation of the HH signaling pathway [85, 89]. Cancer susceptibility syndromes such as Guerlain syndrome [90, 91] and various cancers such as glioma are associated with upregulation of HH [92, 93]. Signaling pathways are activated by three of the HH ligands, namely, desert hedgehog (DHH), Indian hedgehog (IHH), and sonic hedgehog (SHH) [94]. HH ligands activate signaling pathways by binding to patch receptors (PTCH) that override smoothing (SMO) receptors as shown in Fig. 2 [85]. The CDO mediates the binding of PTCH to HH, a binding-reducing cell adhesion molecule that is regulated by oncogenes, GAS1, and BOC [95, 96]. Activated SMO inhibits the suppressor of fused (SUFU) gene action, thus preventing degradation of the zinc finger (GLI) of the glioma-associated oncogene family (GLI) [86]. The GLI family includes three transcription factors (GLI1, 2, and 3) [97]. Activated GLI1 upregulates several genes like PTCH1GLI1, VEGF, Bcl-2, and cyclin D2 (CCND2). In this way, the HH signaling pathway upregulation is related to glioma. Expression of the HH signaling pathway is associated with glioma growth and progression through cancer stem cell augmentation [98, 99]. SHH ligands are highly expressed in both gliomas and surrounding tissues [98, 100]. GBM aggressiveness is thought to correlate well with truncated tGLI1 isoforms [101, 102]. GBM cells in contrast to normal healthy cells express tGLI1 [101]. Migration and invasion of glioma cells are reduced by inhibiting the HH signaling pathway [103, 104]. Bromodomain-containing protein 4 regulates GLI1 transcription by directly binding to gene promoters [105, 106]. Furthermore, lysine acetyltransferase 2B levels correlate with the expression of HH target gene [107]. The HH signaling through cancer stem cell maintenance has also been suggested to play an important role in GBM development and progression [98, 99]. Therefore, in GBM treatment, the signaling cascade is considered a promising target. In this regard, epigenetic regulators may have a crucial role in cancer improvement by deregulating the HH signaling cascade [107, 108]. Consequently, HH signaling could be a useful therapeutic target for GBM.

#### Wingless signaling pathways in GBM

**Fig. 3:**
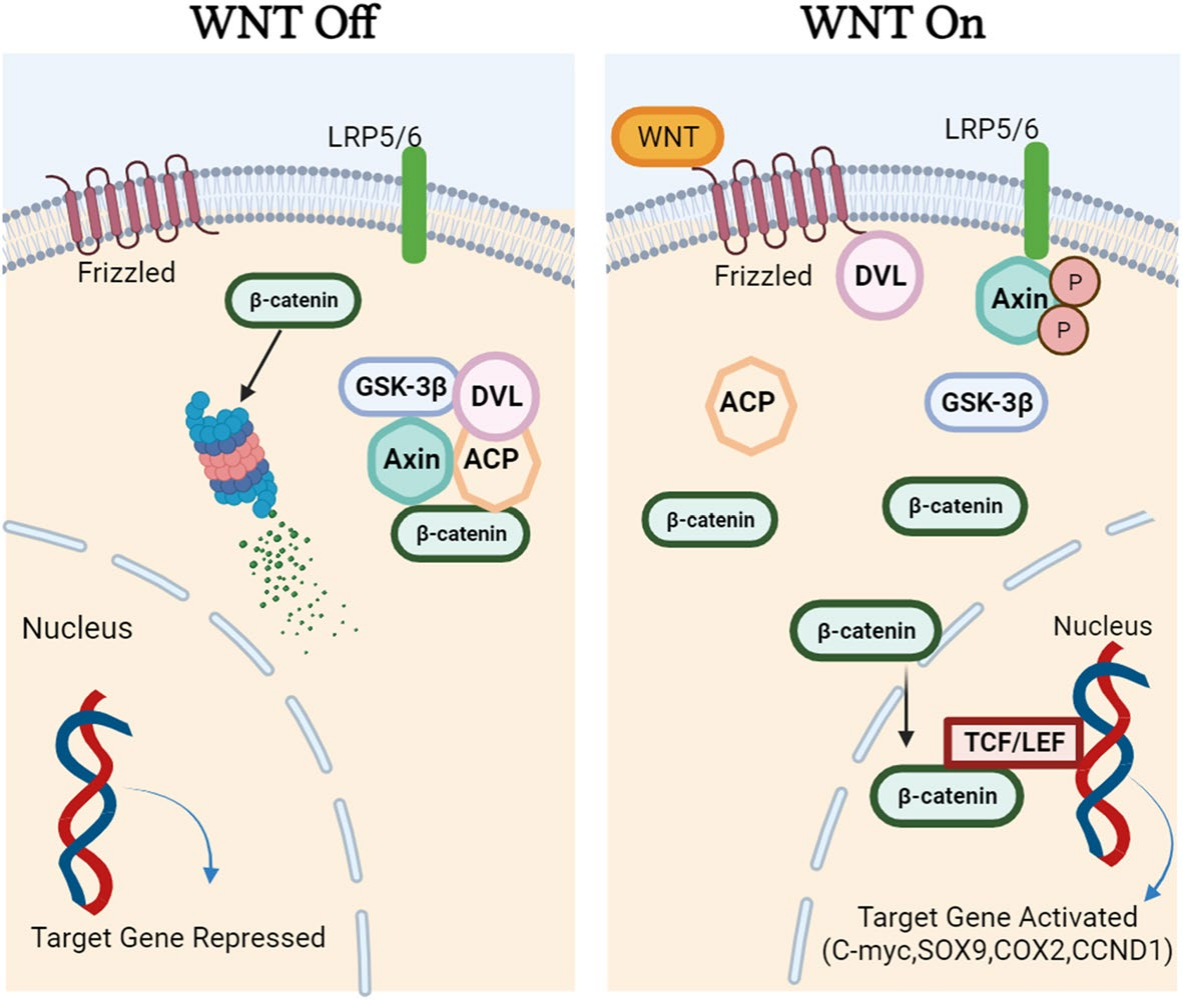
Schematic illustration of the WNT signaling pathway. Left: Inactivation of the WNT signaling pathway. Right: The binding of a WNT ligand to the FZD receptor activates the WNT signaling pathway. FZD, frizzled receptor; LRP5/6, low-density lipoprotein receptor-related proteins; APC, adenomatous polyposis colon. GSK3β, glycogen synthase kinase 3β. DVL, disturbed. CCND1: Cyclin D1; TCF/LEF: T cell agent/lymphoid enhancer agent

The wingless (WNT) signaling plays a critical role in the development, proliferation, migration, and final fate of embryonic cells [109, 110]. It also regulates the adult stem cells’ differentiation, regeneration, and maintenance [111]. Deregulation of WNT signaling causes various CNS pathologies [112, 113] and various tumors, including GBM [75, 114–116]. Importantly, Fig. 3 shows the WNT signaling pathway. The binding of WNT ligands to the cell membrane frizzled receptors (FZDs) activates this signaling pathway [117]. WNT signaling activation inhibits glycogen synthase-3 (GSK-3) and leads to cytosolic *β*-catenin stabilization [81, 118]. GSK-3 plays a role in promoting WNT-FZD complex formation and phosphorylation and degradation of the *β*-catenin [81]. During activation of the WNT signaling pathway, a high proportion of cytoplasmic *β*-catenin translocates to the nucleus, making multimeric complexes through binding to the transcription factors like T cell factor/lymphocyte-enhancing factor (TCF/LEF), which inhibits target genes transcription As c-Myc, CCND1, CD44 SOX9, and COX2 [119–121]. Aberrant WNT signaling cascades regulate various pathways involved in the maintenance of stem cells [122] and therapeutic resistance [123]. Unlike some other cancers, changes in the WNT signaling pathway leading to constitutively active signalings are rare in gliomas. Nevertheless, it is also approved that WNTs play a key role in the dysregulation cascade of glioma stem cells [124]. Furthermore, WNT signaling pathway alterations distinguish between healthy and glioma tissue. The expression intensity of beta-catenin and TCF4 (its transcription factor) is higher in glioma cells than in normal brain cells [125]. In high-grade gliomas, some WNT signaling activators, such as TCF4 and SOX, are increased [75, 126]. In addition, oncogenic phenomena such as cell proliferation, apoptosis, and inhibition of invasion are thought to be associated with the Wnt/β-catenin signaling pathway in GBM [127]. Other WNT signaling factors like FZD1, DKK1, and LEF1 are expressed at more than normal levels in glioma and are associated with poor disease consequences [128]. Active oncogenic activity in glioma cellular populations is thought to be related to changes in the WNT/β-catenin signaling pathway [128]. These results are supported by the fact that chemoresistance and radiation are associated with changes in standard WNT signaling [129]. Several studies have shown that advanced GBM invasion and poor prognosis are associated with WNT expression [130]. High-grade gliomas are associated with both WNT signaling pathway factors, such as LEF1 and HOXA13, which promote glioma growth and cell migration [131]. This is in line with studies showing that WIF-1 loss increases tumor invasion through mediating metastasis-associated lung adenocarcinoma transcript-1 (MALAT1) activity [132].

### Epigenetic alterations in GBM

#### DNA methylation

**Fig. 4:**
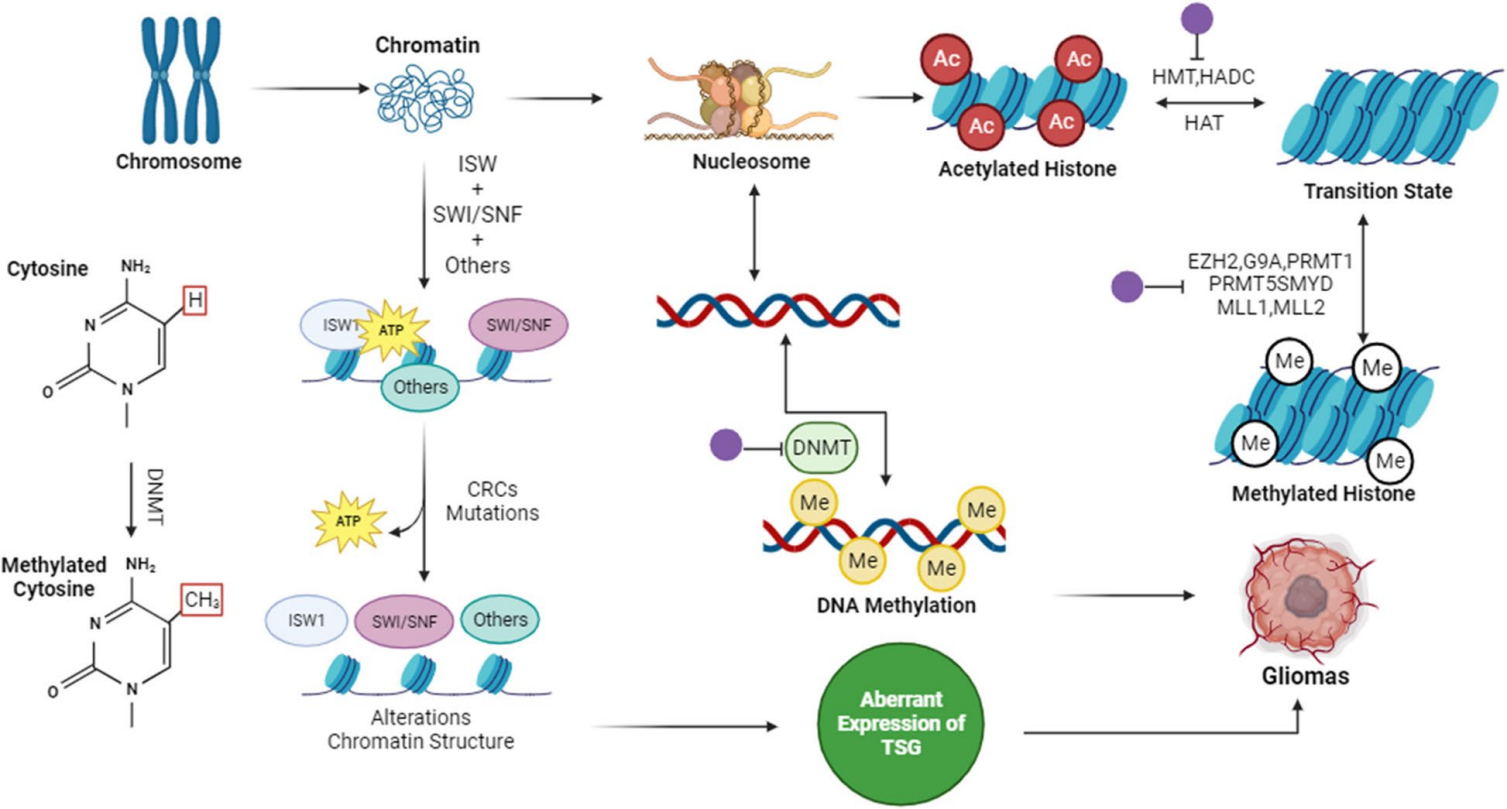
Performance of epigenetic-based therapeutic landscape in the treatment of glioma. Nucleosomes are formed and organized by chromatin as a result of DNA strands coiled near histone octamers. In general, reactions in the presence of DNMTs lead to DNA methylation at the 5-carbon position of cytosine residues (CpG sites), ultimately leading to glioma development. Epigenetic-based therapeutic targets inhibit DNMT, but DNMT also plays an important role in reactivating TSG and related genes to treat glioma. In addition, acetylation, methylation, and phosphorylation reactions lead to histone modifications (HM) at multiple sites. Epigenetic-based therapeutic strategies inhibit HMT and HDAC function, freeing up more sites on histone ends for acetylation, a process that reverses aberrant HM and ultimately tumor cell proliferation may inhibit and cause apoptosis. In addition, chromatin remodeling combinations comprising ISW I and SWI/SNF rely on ATP hydrolysis to provide energy to complete changes in chromatin structure. Mutations in the CRC protein led to an aberrant expression of TSG or other genes included in cell cycle control, leading to the development of glioma. ATP, adenosine triphosphate; DNA, deoxyribonucleic acid. DNMT, DNA methyltransferase; HM, histone modifications. TSG, tumor suppressor gene; HMT, histone methyltransferase; HDAC, histone deacetylase

DNA methylation is believed to be one of the most important discoveries of epigenetic modification processes. There are four possible positions, including the C-5 position of cytosine, the N-4 position of cytosine, the N-6 position of adenine, and the N-7 position of guanine [133]. Also, 5’-CpG-3' cytosines to generate 5-methylcytosine (5mC) take place in DNA methylation mainly in the mammalian cells. The methylation reaction is also performed by a DNA methyltransferase (DNMT) that uses a methyl donor group called the *S*-adenosylmethionine cofactor [134]. DNA methylation patterns of glioma cells and normal cells have been shown in many studies [135] to be different. It is noteworthy that the simultaneous presence of general hyper- and hypomethylation levels of CpG islands is another characteristic of cancer cells. Therefore, in glioma cells, the status of DNA methylation in various related genes is the standard biomarker for the diagnosis of GBM (Fig. 4) [136]. In the promoter region of the gene, DNA gain is the most frequent epigenetic alteration in tumor cells. Also, the regulation of gene expression in the human genome is dependent on the methylation status of the promoter regions. In addition, promoter regions control approximately 50% of tissue-specific genes and all constitutive genes. Nearly all CpG islands in normal physiological conditions are hypomethylated, but some constituent genes, such as DNA repair genes, and tumor suppressor genes (TSGs) are commonly hypermethylated in tumor tissue. This abnormal status of methylation plays an important function in repressing gene transcription and gene’s biological function loss. Also, 5-hydroxymethylcytosine is an epigenetic marker of 5mC oxidation and plays a role in glioma development. In this regard, it has been confirmed that tumor grade has a negative correlation with low levels of 5hmC DNA [137]. Furthermore, in patients of all ages, the CpG island methylator phenotype (G-CIMP) is suggested to be a prognostic marker of glioma [138, 139]. In particular, Jha et al. indicated distinct methylomas in pediatric GBM compared with geriatric GBM. This study indicates that the G-CIMP prognosis marker of glioma in senile GBM cannot be readily extrapolated to GBM in pediatric patients and that there is an urgent need to identify clear prognostic indicators. Also, aberrant DNA methylation is an important marker of TSG inactivation. Several TSGs have also been recognized in gliomas comprising p14ARF, p16INK4a [140], MLH1, and NDRG2 [141]. In addition, the p16INK4a gene maintains the dephosphorylated activated state of the retinoblastoma tumor suppressor protein in the normal cyclin D-Rb cascade and regulates cell cycle progression. Over 50% of homozygous deletions of the p16INK4a gene were also detected in GBM tissue, and p16INK4a is altered in 80% of glioma cells. Thus, restoration of p16INK4a inhibits cell proliferation and induces cell cycle arrest [140]. In addition, MGMT (O-6-MethylGuanine DNA Methyltransferase) is a key DNA damage repair gene that can repair alkyl damage induced by BCNU (bis-chloroethylnitrosourea). Approximately 40% of glioma tissues have been observed to have hypermethylation of MGMT promoter [142]. The degree of methylation has a strong association with tumor prognosis and incidence. Therefore, its importance could be more than prognosis according to tumor grade or age. Several studies have shown that the degree of MGMT promoter methylation is the most important marker for assessing sensitivity to temozolomide (TMZ) in glioma treatment. Conversely, downregulated MGMT could significantly restore TMZ chemosensitivity in vivo and in vitro [143]. In addition to the above genes, CpG islands methylation in the promoter regions of LATS1, LATS2 [144], and p73 [145], and the genes described in Table 1 are also strongly related to GBM progression.

**Table 1:** DNA methylation of genes or proteins in glioma cells

Genes/Proteins	Location on chromosome	Functions	Symbol	References
ARF tumor suppressor	9	Controls cell cycle	P14ARF	[145, 146]
Cyclin-dependent kinase inhibitor 2A	9	Inhibits cell proliferation and triggers cell growth arrest	CDKN2A/p16INK4a	[147]
Tumor protein P73	1	Reduces cell proliferation and enhanced apoptosis	P73	[148, 149]
Mitogen-activated protein kinase	8	Inhibits cell growth	MKP-2	[150]
Nuclear receptor binding SET domain protein 1	5	Suppresses cell growth	NSD1	[151]
MicroRNA 129–2	11	Suppresses cell growth, and triggers apoptosis	miR129-2	[152, 153]
HIV-1 tat interactive protein 2	11	Suppress cell growth and proliferation	HTATIP2	[154]
Solute carrier family22 member 18	11	Inhibits cell growth and induces apoptosis	SLC22A18	[155–157]
TNF receptor superfamily member 11a	18	Elevates cell apoptosis	RANK(TNFRSF11A)	[158]
Neogenin	15	Induces cell apoptosis	NEO1	[159, 160]
Nonsteroidal anti-inflammatory drug-activated gene-1	19	Induces cell apoptosis and Inhibits cell growth	NAG-1	[161]
Glioma pathogenesis-related protein 1	12	Promotes cell apoptosis	GLIPR1	[162]
Testin	7	Triggers cell apoptosis	TES	[163]
Brain expressed X-linked1	X	Increases sensitivity to chemotherapy-induced apoptosis	BEX1	[164]
Brain expressed X-linked2	X	Enhances apoptosis, as well as inhibits migration and invasion	BEX2	[164, 165]
N-Myc downstream-regulated gene 2	14	Decreases cell proliferation	NDRG2	[166, 167]
Human mutl homolog 1	3	Repairs damage DNA	hMLH1	[168]
O^6^-alkylguanine DNA alkyl transferase	10	Repairs damage DNA	MGMT	[169–171]
Epithelial membrane protein 3	19	Reduces cell proliferation	EMP3	[172, 173]
Kruppel-like factor 4	9	Decreases cell proliferation	KLF4	[173–175]
WNK lysine deficient protein kinase 2	9	Suppresses cell invasion	WNK 2	[176]
Slit guidance ligand 2	4	Inhibits cell migration and invasion	SLIT 2	[177]
Micro RNA 124–1	8	Inhibits cell proliferation, invasion, and migration	miR-124a	[178, 179]
Tissue factor pathway inhibitor 2	7	Suppresses cell proliferation, invasion, and migration	TFPI-2	[180–182]
Protocadherin 10	4	Triggers the cell growth arrest and apoptosis	PCDH10	[183]
RUNX family transcription factor 3	1	Suppresses cell migration and invasion	RUNX3	[184]

#### Histone modifications in GBM

Histone modifications (HM) occur in different ways in the epigenome of mammalian species. The histone building block, the nucleosome, is an octamer consisting of 147 compositionally coiled base pairs with two H2A, two H2B, two H3, and two H4s. Correspondingly, nuclear histones have both N-terminal and C-terminal binding sites. Relatively, because the N-terminal half of lysine extends outside the nucleosome, the N terminus has a significance situation and is critical to modifications comprising acetylation, methylation, ADP-ribosylation, ubiquitination, and phosphorylation. For gene expression changes without alterations of base pairs, these modifications and differences play an important role. Transcription errors permanently take place in the expression of various genes that have an important function in the progress and development of glioma, and these transcription errors may be due to misplaced HMs. Among the various HM proteins, histone methyltransferases, histone deacetylation, and two proteins that cause methylation at multiple sites on histones have received more consideration than other HDACs. Interestingly, HDAC, HDAC1, HDAC2, HDAC3, HDAC5, and HDAC9 enzymes from diverse classifications in glioma cell lines have main alterations and differences, as well as the HDAC5 and HDAC9 expression in high-grade medulloblastoma, the level of H3 acetylation in astrocytoma of high-grade compared to low-grade medulloblastoma and considerably the normal tissue of the brain increases. Notably, type II mRNA levels and class IV HDAC levels had downregulation results in GBM in comparison with low-grade astrocytoma and healthy and normal brain tissue. In this way, the application of HDAC inhibitors (HDACIs) has become an active and practical research category for curing some different cancers. In addition, HDACIs have been employed combined with radiotherapy and chemotherapy to cure and control GBM. Meanwhile, the mechanisms of antitumor activity of HDACIs, comprising promoting cell differentiation, inducing apoptosis, inhibiting angiogenesis, and blocking the cell cycle, may eventually prevent and control the proliferation and programmed cell death of various tumor cells [147]. Several recent studies have indicated and confirmed that the degree of histone lysine methylation is regulated by histone methyltransferases containing EZH2, MLL1, MLL2, and G9a in all types of glioma cells. These modifications are closely correlated to genome integrity and gene transcription regulation [166]. The protein arginine methyltransferase 5 (PRMT5) gene is used in the diagnosis and treatment of GBM, whose nuclear expression is associated with poor survival in glioma patients. GBM cell treatment with a PRMT5 inhibitor mirrored the action of PRMT5 knockdown and played and led to the apoptosis of differentiated GBM cells [168]. It is acknowledged that glioma cell proliferation inhibition and apoptosis activation may be achieved through suppressing the function of histone methyltransferases or HDACs [185], recommending that these proteins It has been suggested that the suppressor could be used as a potential drug to treat glioma. A recent study showed that the G9a histone methyltransferase, which regulates the demethylation of H3K9, is also associated with glioma progression [186]. Therefore, its suppressor is considered a promising candidate for the treatment of glioma [187]. Recent studies [188] have shown overexpression of EZH2 in many tumor tissues, such as tumor tissue. It is a glioma and is closely associated with cancer cell development, metastasis, and invasion. Advances in clinical research suggest that the use of EZH2 gene silencing techniques or EZH2 suppressors could prevent glioma cell proliferation [189]. Therefore, EZH2 has been suggested as a new target that could open new avenues for the treatment of glioma [190]. In addition, H3F3A contains two changes in the histone tail, a glycine (G) amino acid to arginine (A)/valine (V) amino acid change at codon 34 (G34R/V) and a methylation at lysine (K) 27 (K27). It is one of the key regulators of post-transcriptional modifications in pediatric GBM [146].

#### Chromatin remodeling in GBM

The chromatin remodeling complex (CR) has an adenosine triphosphates (ATPase) function and relies on ATP hydrolysis to provide the energy to fulfill changes in the structure of chromatin [167]. The complexes could be classified as ISW I, SWI/SNF, and others based on the different subunits capable of hydrolyzing ATP. Also, these proteins and complexes that are related to cell cycle inhibition and activation, DNA repair, DNA methylation, and DNA transcription have a significant role. Mutations in the CR protein are associated with many diseases in humans. Additionally, these mutations are responsible for CR failure, which ultimately leads to chromosome misalignment, blocking the transcription machinery and making DNA inaccessible to complexes capable of repairing the damage. This can lead to abnormal gene expression. If these mutations make abnormalities in TSGs or proteins controlling the cell cycle. They may finally contribute to cancer incidence [169]. A recent study found that CR controlled drug resistance in the GBM [170]. Targeting the GBM stem cells with kinase inhibitors could reversibly induce these cells into a slow, cyclical steady state. In addition, this status activates the Notch signaling and significantly upregulates the histone demethylases KDM6A/B. This has a key role in the removal of H3K27 trimethylation in cis-regulatory regions of the genome, subsequently contributing to increased H3K27Ac levels. CR has an important function in these types of cell shifts, and this study provided new targets for future beneficial therapeutic advances. Furthermore, by targeting developmental and epigenetic cascades, it could be possible to destroy drug-resistant tumor cells and prevent recurrence of disease. Research approved that upregulated CR factors, including lymphocyte-specific helicase (LSH), accelerate glioma progression [171]. In addition, this study shows that glycogen synthase kinase-3β (GSK3β) and the regulated transcription factor E2F1 in astrocytoma and GBM are involved in the development of glioma and expression of LSH [171]. Also, reduction of E2F1 decreases the expression of LSH and cell proliferation, while deletion of GSK3β increases its accumulation in E2F1 in the LSH promoter and ultimately increases LSH expression. In this regard, lipoprotein receptor-related protein 6 (LRP6), which serves as an upstream regulator for GSK3β signaling, is often over-expressed in glioma cells. Degradation of LRP6 reduces the recruitment of E2F1 to the promoter of LSH, thus reducing LSH the expression levels. LSH ultimately plays an important role in suppression of cell growth. Overall, there is a mechanistic relationship between expression of LSH in glioma cells and induction of the LPR6/GSK3β/E2F1 axis, suggesting a novel role for LSH in both malignant astrocytoma and GBM. So, understanding the contribution of LSH in the development of gliomas will therefore improve our understanding of gliomas and suggest LSH as a promising therapeutic target in patients with these types of brain tumors.

### Noncoding RNA in GBM

#### Role of microrna in GBM

**Fig. 5:**
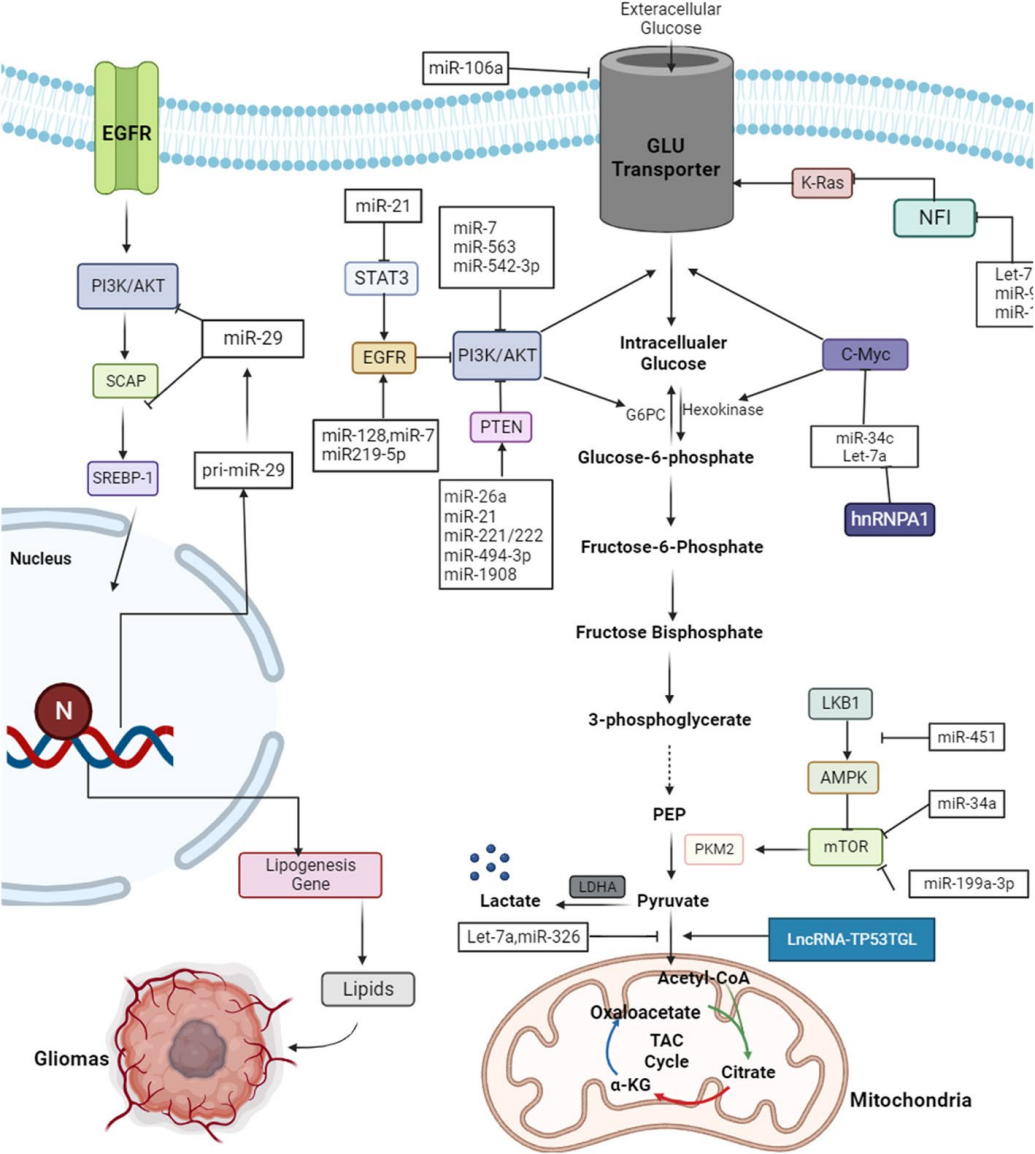
Role of miRNAs in regulating lipid metabolism and glycolysis in glioma. Glycolysis takes place in the cytosol when d-glucose enters the cell through membrane transporters of glucose. After a series of enzymatic reactions, d-glucose is converted to pyruvate and then is entered into the TCA cycle. Conversely, in conditions with limited oxygen levels, pyruvate is converted to lactate. Moreover, miRNAs and long noncoding RNAs control glycolysis in oncogenic conditions. Glucose transporter family expression is regulated by noncoding RNAs, thereby altering glucose internalization rates. It is important to say that the miRNAs also inhibit the F PI3k/AKT signaling, which plays an important role in the metabolism of GBM cells. In addition, miRNAs inhibit c-Myc and mTORC2, which regulate GBM glycolytic metabolism. On the other hand, miRNAs also have a central role in the lipid metabolism of GBM cells. The transcription factor SREBP-1 triggers cholesterol synthesis and is highly upregulated in various tumors, including GBM [187]. Furthermore, SCAP/SREBP-1 signaling mediated by EGFR upregulates miR-29 by binding to precise sites within the promoter. Interaction between miR-29, SREBP-1, and the 3′-UTR of SCAP subsequently represses their expression. Thus, the miR-29-SCAP/SREBP-1 feedback loop regulates EGFR signaling-mediated GBM proliferation through the regulation of cholesterol synthesis: G6PC, glucose-6-phosphatase; G6PD, glucose-6-phosphate dehydrogenase, PEP, phosphoenolpyruvate; PKM2, pyruvate kinase M2; LDHA, lactate dehydrogenase A; α-KG, α-ketoglutarate; EGFR, epidermal growth factor receptor; STAT3, signal converter and transcriptional activator 3; PTEN, phosphatase, and tensin homologs; NF1, neurofibromin 1; AMPK, AMP-activated protein kinase; LKB1, liver kinase B1; PI3K/AKT, phosphatidylinositol 3-kinase/protein kinase B; cMYC, c-myelocytoma virus oncogene homolog; SREBP-1, sterol regulatory element binding protein 1; and SCAP, SREBP cleavage-activating protein

A group of small RNA sequences known as microRNAs (miRNAs) exhibit post-transcriptional regulation during mRNA degradation [148, 172–175]. Considerably, miRNAs are currently being used to control cellular metabolism in GBM. Moreover, as shown in Fig. 5, miRNAs direct the expression of metabolic genes either directly or through the regulation of signaling cascades of cancer [149], metabolic oncogenes, and tumor inhibitors [150]. Some miRNAs can target mRNA enzymes that promote metabolic processes such as lipid, glutamine, and glucose metabolism, oxidative phosphorylation, and glycolysis. Also, this miR-106a gene affects GLUT3 (SLC2A3) and decreases glucose influx during glycolysis [151]. In GBM stem cells, the miR-143 gene affects HKII and induces differentiation [152]. Also, let-7a and miR-326 regulate GBM metabolism through inhibiting PKM2 expression [153, 154]. In addition to glucose metabolism, miRNA-153 also targets glutaminase and downregulates the metabolism of glutamine in glioma cells [155]. miR-100, miR-16, miR-101, and miR-23 target the mitochondrial ATP synthase ATP5B or ATP5A1 to control mitochondrial energy metabolism in GBM cells [156]. Many anticancer factors in its downstream cascade play key roles as controllers in metabolism in GBM cells. Therefore, miRNAs targeting these factors may also indirectly affect the GBM cell metabolism. EGFR is also expressed in high levels in approximately 50% of GBM cells and is also a GBM pathological target with EGFR amplification or mutation. EGFR activates PKCε monoubiquitination, which leads to the induction of NF-κB and an increase in the expression of PKM2 to promote glycolysis and tumorigenesis in GBM [157]. Of note, expression of EGFR is decreased in GBM compared with gliomas of low-grade malignant potential by various miRNAs like miR-7, miR-219-5p, and miR-128 [158–160]. Oncogenic K-Ras induces the growth of cancer cells through dissociating glutamine and glucose metabolism [161]. MiRNAs, including let-7a and miR-134, that target KRAS that are downregulated in GBM are associated with disease-poor prognosis [162]. Similarly, miR-9 indirectly affects K-Ras by targeting neurofibromin 1[163]. In addition, miR-9 is expressed more than normal levels in GBM and this expression is also associated with poor disease prognosis [164]. In this regard, C-Myc is one of the main regulators of metabolism in tumoral cells by increasing the target gene expression like LDH-A, Glut1, glycolytic enolase, and serine hydroxymethyltransferase which increases C1 metabolism [157]. Various studies have shown that Let-7a and miR-34 directly affect c-Myc in GBM [154, 165]. In addition, the PI3K/Akt signaling pathway is also an important controller that has a key role in regulating the Warburg effect in cancer and GBM cell metabolism [176]. miR-542-3p [177] and miR-7 [178] target Akt and PI3K in GBM, respectively. miR-503 [179] also could inhibit the PI3K/Akt signaling. PTEN (a PI3K antagonist) is a major tumor inhibitor of GBM regulated by miR-26a, miR-221/222, miR-21, miR-10a/10b, miR-1908, and miR-494-3p [180–182]. Also, the Warburg effect regulation has a key role in the LKB1-AMPK cascade [183]. On the other hand, mTORC2 affects glycolytic metabolism through acetylation of FOXO and upregulation of c-Myc in GBM [165]. Considerably, miR-199a-3p has been observed to target mTORC2, which is downregulated in GBM compared with normal brains [184]. In addition to the aforementioned mechanisms, regulation of miRNA may be a more promising approach. For example, c-Myc targets let-7a downregulate hnRNPA and subsequently inhibit expression of let-7a by binding to and inhibiting the progression of pri-let-7a [154]. Additionally, let-7a directly targets PKM2. Consequently, the let-7a/c-Myc/hnRNPA1/PKM2 feedback loop upregulates PKM2 in GBMs and induces glycolysis. Interestingly, miRNAs also have a crucial role in GBM lipid metabolism. Indeed, malignant tumors are fundamentally characterized by this metabolic dysregulation. Also, the regulatory element of sterol transcription factor binding protein 1 (SREBP-1) causes cholesterol synthesis and has increased expression in various tumors such as B. GBM [191]. For instance, SCAP/SREBP-1 upregulates miR-29 through EGFR signaling and by binding to precise sites within the promoter. The interaction between miR-29, SREBP-1, and three major untranslated regions (3′-UTR) of SCAP subsequently represses their expression. Thus, the miR-29-SCAP/SREBP-1 feedback loop regulates glioma cell proliferation through the regulation of EGFR signaling and cholesterol synthesis [192]. Approximately 50% of miRNA genes are thought to reside in cells of glioma or their susceptibility sites, and these genes may regulate approximately 3% of all tumor genes of glioma and 30% of coding genes there is. Similarly, one miRNA could affect 100 GBM mRNAs simultaneously [193], whereas one mRNA in glioma can relax one or multiple miRNAs [194]. This study suggested that many miRNAs are altered in GBM, ultimately affecting the regulation of mRNAs associated with gene expression [195]. Correspondingly, miRNAs have several important functions in glioma proliferation. In particular, miRNAs have a significant role in modulating the expression of cancer genes and genes involved in tumorigenesis and regulating different signaling pathways. These are viruses that also regulate glioma stem cell differentiation, are encoded as oncolytic, and play an important role in the growth of tumors [196]. In comparison, the knockdown of miR-221/222 decreased cell invasion by altering TIMP3 levels of the tissue inhibitor of metalloproteinase-2. Degradation of miR-221/222 further enhanced the expression of TIMP3 and significantly shortened the development of tumors in xenograft models. Another study found that overexpression of miR-221/222 decreased p27kipl-level staging [197]. P27kipl prevents cell cycle transition from the G1 phase to the S phase through binding to the cyclin E complex and CDK2. Therefore, downregulated miR-221/222 may have an up regulatory effect on p27kipl to suppress tumor growth [194]. In cancer metabolism, circular RNAs and long non-coding RNAs (lncRNAs) play a very important role [198] and play a central role in gene regulation [199–202]. It is confirmed that 198 lncRNAs were downregulated and 27 lncRNAs were upregulated in GBM, indicating the critical role of these nucleic acids in GBM. Recently, there has been some evidence suggesting that lncRNAs are also involved in the cellular metabolism of GBM. For example, TP53TG1 lncRNA supports cell proliferation and migration by expressing genes like IDH1 and PKM2 in glioma cultures with reduced levels of glucose [203]. The lncRNA LEF1-AS1 increases GBM cell proliferation and prevents apoptosis through the Akt/mTOR signaling, which controls glycolysis [22]. However, further investigations are needed to confirm the association of lncRNAs and GBM regulation. In contrast, circular RNAs have an important role in the GBM cellular metabolism. For example, Fbxw7circRNA translates a novel 21 kDa protein called FBXW7-185aa, which reduces USP28-mediated stabilization of c-Myc [204]. This may be related to the Warburg effect. However, little is known about the role of circRNAs in the regulation of cell metabolism [205].

### Therapeutic approaches for GBM therapy

Mesenchymal stem cells could suppress the development, invasion, and metastasis of hard tumors. They are therefore considered to be excellent therapeutic modalities to treat tumors, but their exact role in tumorigenesis is currently unknown [206]. The response of GBM tumors to surgery, chemotherapy, and radiation is not completely clear, so new treatments are greatly needed [207]. Because miRNAs affect the expression of various genes, they are potential candidates for GBM therapy. For example, miR-873 downregulates IGF2BP1 expression which results in decreased carcinogenesis and metastasis of GBM [208]. Instead, miR610 reduces cell proliferation and GBM proliferation by inhibiting the expression of CCND2 and AKT3 at translational as well as transcriptional levels [209]. In this account, lncRNAs such as ASLNC20819 and ASLNC22381 that target IGF-1 play several crucial roles in GBM progression. Targeted therapy of lncRNAs is therefore likely to be an effective therapeutic approach [210]. Compared to normal cells, altered epigenetic changes occur in tumor cells, which can be inhibited by using inhibitors that alter the activity of epigenetic enzymes (EEs). In this regard, for example, a potential epigenetic therapeutic agent such as 5-aza-2'-deoxycytidine (5-aza-CdR) increases GBM cell apoptosis through the caspase-8 pathway [211].

### Epigenetic drugs for GBM therapy

Epigenetic therapies have been investigated in clinical trials, but only a few have received approval from both the European Medicines Agency and the Food and Drug Administration for use in treating cancer [212]. Epigenetic regulators such as BMI1 and EZH2 are useful in vitro and in vivo. The application of EZH2 inhibitors aids control of the progression of GBM [210]. Agents that inhibit DNMT1 are said to reduce DNA methylation and possibly activate tumor suppressor genes. For example, decitabine and azacitidine, which belong to the DNMT inhibitor known as 5-aza-CdR, are a type of FDA-approved epigenetic drugs for the treatment of medulloblastoma, myelodysplastic syndrome, and acute myeloid leukemia [213, 214]. HDACI, a histone deacetylase inhibitor, blocks the glioma gene transcription and further influences the cell cycle. They work by blocking cell division in the G1 and G2 phases, which in turn promote apoptosis and cell differentiation [215]. HDACIs can reduce glioma growth and development by further degrading a combination of heat shock and matrix proteins and inhibiting angiogenesis in tumors [216]. DNMT and HDACI inhibitors may be used individually or synergistically in combination with other agents to treat various tumors [217]. Therefore, HDACIs offer new opportunities as therapeutic agents for GBM. Studies of HDACI are ongoing and include phase I and II trials [218, 219]. A combination of temozolomide and vorinostat in a clinical phase I study which was conducted by the Children’s Oncology Group, suggested that the combination of vorinostat and TMZ is recommended for refractory or relapsed primary tumors of CNS [220]. Vorinostat and TMZ combination was well tolerated for 5 days in pediatrics with recurrent malignancies of CNS, and the dose-limiting toxicity was myelosuppression. Vorinostat causes acetylated H3 accumulation in peripheral blood mononuclear cells. A phase II trial investigating vorinostat application for recurrent cases of GBM was conducted by the North Central Cancer Treatment Group [221]. This study demonstrated that vorinostat monotherapy was beneficial in recurrent GBM. After treatment, the acetylation rates of H2B, H3, and Hand4 were significantly increased. RNA microarray analysis reveals changes in vorinuler-regulated genes with E-cadherin upregulation [194].

### *Role of CAR T cell* t*herapy in GBM*

CAR T cells (chimeric antigen receptor T cell) are allogeneic and autologous modified T cells obtained from a patient’s peripheral blood and expanded in vitro. They are genetically engineered using electroporation or viral vectors to express CAR cell membrane molecules. Their extracellular domains may identify tumor-associated antigens, and their intracellular domains contain signals that activate T cells. The modified T cells then are injected into the patient’s body, where they will identify cells bearing the corresponding tumor antigens [222]. The TCR-CD3 complex has six independent genetic products. CD3 g, d, ϵ, z, and TCR a, b chains. TCR a and b chains may bind to HLA-peptide complexes. The g, d, ϵ, and z chains of CD3 could activate T cells [223]. The intracellular signaling domains of the activated T cells typically contain signaling domains that are recognized as first-generation CARs even in the absence of other signaling domains. The addition of costimulatory signaling domains (usually 41BB or CD28) creates second-generation CARs. The third-generation CARs arise from the combination of many different co-stimulatory proteins and several co-stimulatory domains [224]. This is thought to stimulate T cell production, leading to cancer cell killing by cytotoxic cells [225, 226]. In a phase 2 trial of patients with refractory or relapsed B cell acute lymphoblastic leukemia, nearly 81% of the patients achieved remission 3 months after CAR-T cell therapy. The survival rate after 6 months was 73% and the event-free rate was 90%. Furthermore, after 12-month the survival rate and event-free rate were around 50% and 76%, respectively [227]. Another phase 1–2 trail involving 22 centers reached similar conclusions [228]. In addition to the successful clinical experiences in hematological malignancies mentioned above, many of these CAR-T therapies have also been demonstrated in some other solid malignancies such as GBM [229], pancreatic [230], colorectal [231], and renal cell disease. Clinical trials are underway [232], for ovarian cancer [233], and breast cancer [234]. Although CAR-T therapy has not yet been clinically implemented in solid tumors, it offers hope for patients with other types of cancer who have few therapeutic options.

### Clinical application of AUTO-T cells for GBM

CAR-T cell application in GBM patients is still limited because GBM does not express tumor-specific antigens [235]. However, with the advent of this CAR in the second and third generations overcoming the low heterogeneity of GBM tumors has been made possible which resulted in improved clinical efficacy. This includes studies on various CAR-T cell targets and combined therapeutic strategies like combination immune checkpoint blockade and chemotherapy. Nevertheless, only three of these trials reported clinical responses for CAR-T cell targets. Importantly, interleukin 13 receptor alpha 2 (IL13-Ra2) [236], epidermal growth factor receptor variant III (EGFRvIII) [237], and human epidermal growth factor receptor 2 (HER2) [238], are clinically shown to be an effective and safe target for the efficacy of CAR-T cell therapy in GBM disease.

### *Allogeneic CAR-T* c*ells for GBM* t*herapy*

Although allogeneic T cells have many superiorities over autologous T cells, they pose unique challenges that must be addressed to achieve clinical success. These challenges include (A) proper selection of T cell sources, as well as (B) avoidance of GVHD and (C), and host immune rejection to achieve potent activation and proliferation in vivo [239].

### Source of T cells for GBM therapy

**Fig. 6:**
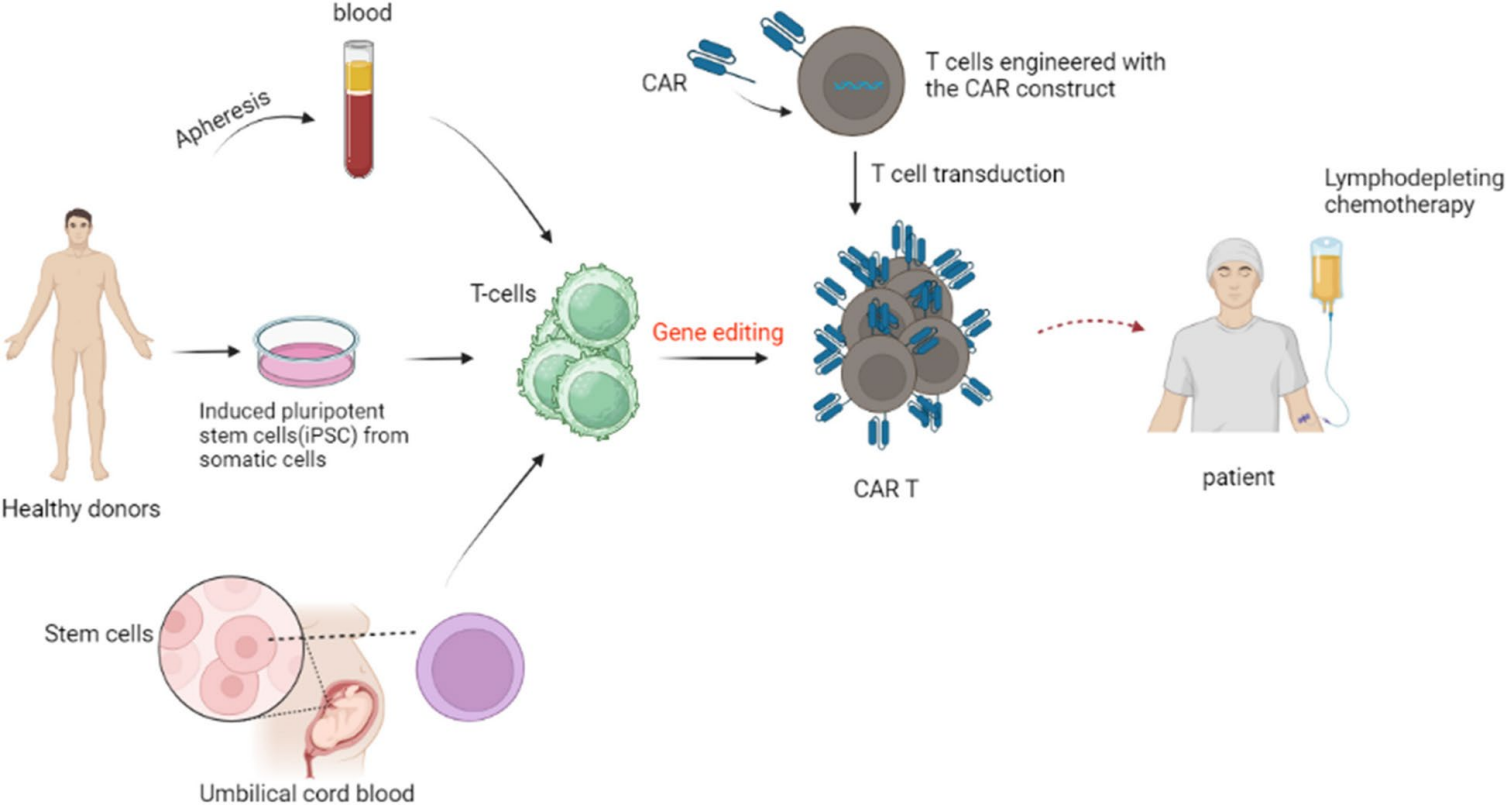
Generation of allogeneic (“standard”) CAR T cells and T cell sources. Allogeneic T cells could be obtained from healthy donor peripheral blood mononuclear cells, cord blood, or induced pluripotent stem cells (iPSCs). CAR-T cells are produced by viral transduction and in vitro expansion

Non-mobilized peripheral blood leukapheresis derived from the patient is the main and most commonly used material to start the production of autologous CAR-T cells. In contrast, in healthy adult volunteers apheresis is conducted in an allogeneic setting [240] (Fig. 6). Recruiting healthy donors yields a large number of donated cells from every single subject. Peripheral blood mononuclear cells are preferred because donors, unlike patients with cancer, do not undergo radiotherapy or chemotherapy [239]. Other cell sources, such as umbilical cord blood (UCB)-derived T cells, could also be considered for the development of allogeneic CAR-T cells. Application of UCB-derived T cells reduces the activation of the NF-κB pathway resulting in decreased responsiveness and consequent reduced generation of many pro-inflammatory cytokines, thus decreasing the frequency and intensity of GVHD. [241]. In the hematopoietic stem cell transplantation (SCT) as a therapy for hematological malignancies, transplantation of UCB has a better outcome than the corresponding unrelated donor in terms of incidence of GVHD, late effects, and overall survival, showing similar outcomes compared to matched related-donor transplants [242–244]. UCB-derived CAR-T cells have already been used, demonstrating the feasibility and efficacy of this approach, as UCB-derived CAR-T cells can identify and destroy target cells [245]. Another promising choice is induced pluripotent stem cells (iPSCs). This makes it possible to generate pluripotent stem cells using adult somatic cells by introducing specific transcription factors [246]. In this way, iPSC-derived T cells pose longer telomeres which results in higher proliferation capacity in comparison with mature T cells. So far, one study has shown that anti-CD19 CARs are derived from iPSC-derived T cells and that these CAR T cells can specifically recognize and kill target cells [247]. However, no major progress in generating CAR-T cells using iPSCs has been achieved recently. Given that GVHD is a leading cause of death post allogeneic SCT, the main focus has been placed on generating allogeneic CAR-T cells to avoid GVHD [248]. In recent years, many groups have worked to improve the classification and diagnosis of GVHD. Currently, there is a consensus on defining two primary GVHD categories, acute and chronic [249]. Nevertheless, the investigations on CAR-T cells, in particular allogeneic CAR-T cells, do not mention the differential effect of these approaches on each category of GVHD, especially chronic GVHD. With the advent of allogeneic CAR-T cells in the clinical setting, further studies are needed to clarify their effect on both categories of GVHD. Many groups hypothesize that the primary cause of GVHD is ab T cells, the most commonly used type of T cells to develop CAR T cells [250]. Two primary strategies have been proposed to reduce the risk of GVHD, based on either virus-specific T cell selection or genetic TCR locus ablation. Because the alloreactivity risk increases with the diversity of the donor’s TCR repertoire and the number of transferred T cells [239], there are reasons to use purified T cells having low diversity of TCR repertoire. Indeed, the application of virus-specific memory T cells in hematopoietic SCT may control viral infection without inducing GVHD [251, 252]. Repeated stimulation of donor T cells may reduce the GVHD risk by increasing the frequency of virus-specific memory cells, but predicting the extent of alloreactivity of these cells in advance remains challenging [253]. A small clinical study applying allogeneic virus-specific T cells that express anti-CD19 CAR constructs showed that they were safe and could exert antitumor activity without clinically developing GVHD [254]. A new clinical trial is underway with anti-CD30 and anti-CD19 CAR T cells modulated with Epstein-Barr virus-specific allogeneic T cells [255]. Using virus-specific T cells as a source for allogeneic CAR T cells remains a promising approach that needs to be investigated in next-generation clinical studies. In recent years, the robust development of technologies for gene editing has made available the main tools needed to block endogenous TCR expression and minimize the GVHD risk (Fig. 2). Various groups have reported that by genetically knocking out the exons of the TCRa constant (TRAC) and/or TCRb constant 1 or 2 (TRBC1 or 2) loci using small interfering RNAs, T cell surface eliminates expression of the ab TCR [256], ZFNs [257], TALENs [258], MegaTAL nucleases [259], artificial homing endonucleases, or CRISPR/Cas9 [260]. A direct comparison between TALENs, megaTAL nucleases, and CRISPR/Cas9 showed that the latter two were the best at disruption of TCR [259]. As there is only one α-chain constant region gene, this is considered the most efficient and direct approach to disrupt the ab-TCR and is, therefore, the most commonly used [261]. Additionally, further modifying CAR-T cells is possible by multiplexing. Indeed, the CRISPR/Cas9 technique has been applied to produce MHC class I- and TCR-deficient allogeneic CAR T cells supplemented with PD1 [262], PD1/CTLA4, or Fas, knockout. Multiple gene editing may help reduce the alloreactivity of CAR-T cells while enhancing their resistance to immunosuppression and apoptosis. However, it also improves the off-target cleavage risk, which may lead to hyperproliferation of CAR-T cells because of tumor suppressor gene disruption [263]. One of the most intriguing alternatives to avoid GVHD and achieve functional advantage in a more controlled manner is the direct introduction of CAR transgene into the TRAC locus. Indeed, in addition to the reduction in GVHD, this manipulation allows regulated and homogeneous CAR expression under the control of the TCR promoter. This is a trait that has been shown to lead to reduced differentiation and depletion of CAR T cells [261, 264]. This mutant has similar advantages, and it has also been studied in the context of TCR-manipulated T cells [265]. Other strategies that have been proposed to reduce the risk of GVHD include the application of non-Ab T cells [266] or T cells derived from hematopoietic SCT donors. The first involves populations of innate lymphocytes like NK [267], gd-T cells [268], or invariant NKT cells (iNKT) [269]. In the case of gd-T cells, these rare cells (5% of T lymphocytes) can proliferate in vitro, exhibit potent anti-tumor cytotoxic activity, and identify targets independently of restriction of MHC, possible and unlikely to cause GVHD [270]. Preclinical experiments with CAR-gd T cells have shown some promising results, including glioma-associated targets such as disialoganglioside GD2 [271]. NK cells and iNKT cells will be explained in detail later. The use of T cells from SCT donors is limited to cases who relapse after allogeneic hematopoietic SCT. Here, the same donated CAR-T cells could be used in case of relapse, and this procedure showed GVHD in only 6.9% of cases in a meta-analysis conducted on seven studies [272].

**Fig. 7:**
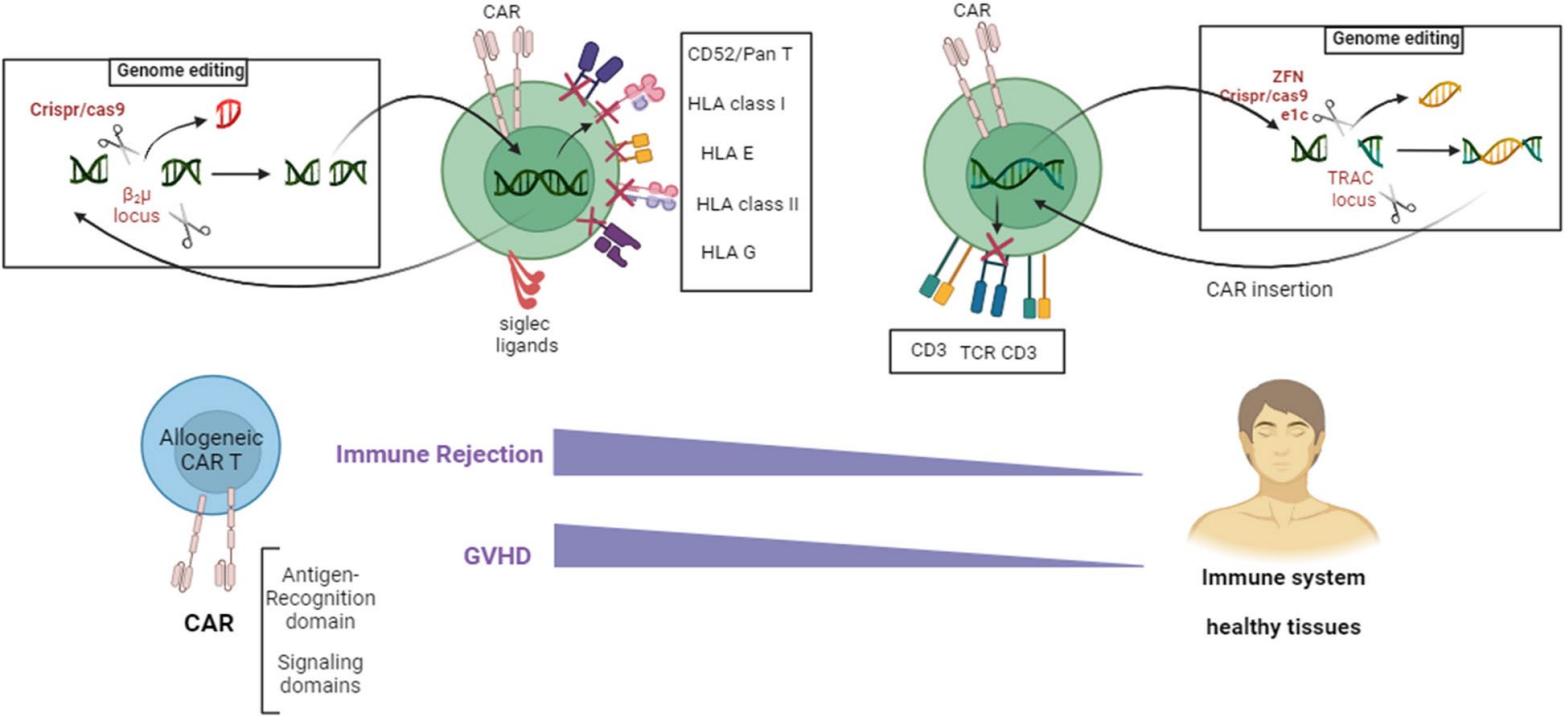
Allogeneic CAR-T cells must evade host immune rejection and GVHD. Allogeneic CAR-T cells evade the patient's immune response by genetically disrupting HLA class I and II molecules, resist anti-CD52 antibody lymphocyte ablative, therapy by removing CD52 molecules, and reduce HLA able to inhibit NK removal by increasing expression of Siglec ligands of -E and G variants. To protect a patient from GVHD, allogeneic CAR-T cells can be engineered to lose TCR expression

Another important challenge in therapies using allogeneic CAR-T cells is that these cells must be kept and expanded in vivo. This property has been demonstrated in studies of autologous CAR-T cells in neuroblastoma and hematologic malignancies [273]. It is associated with response to therapy [274]. As previously mentioned, allogeneic CAR-T cells do not have the same limitations as autologous cells. A major concern in enhancing T cell function and thus persistence in vivo is to decrease its immunogenicity. Repeated administration is possible because allogeneic CAR-T cells can be produced in higher numbers compared to autologous CAR-T cells (Fig. 7). Some early findings using this approach as an attempt to circumvent transplant rejection in vivo showed its feasibility [275]. However, repeated dosing needs repeated immunosuppression of the patient, and repeated encounters with the host’s immune cells increase allogeneic reaction risk, at least with antibody generation from past transfusions. Aiming for long-term persistence of lymphopenia is also an option, but it is necessary to generate CAR-T cells that can resist lymphopenic agents. To this end, ab-TCR-deficient CAR-T cells, rendered resistant to several purine nucleotide analogs with the deletion of the deoxycytidine kinase gene, can efficiently function in the presence of agents with lymphodepleting potential and can kill tumor cells [276]. In addition, CAR-T cells were rendered resistant to depletion by knocking out CD52 using an anti-CD52 monoclonal antibody (alemtuzumab) applied as a preconditioning regimen [277]. Regardless of the number of infusions or the strength of lymphodepletion, it is always desirable to reduce the allogeneic CAR-T cell’s immunogenicity, and a direct method is the genetic nullification of class I MHC molecules. Although they are highly polymorphic molecules, they all are the same in having the b2 microglobulin protein and disruption of this subunit allows the removal of all surface MHC class I molecules on the T cell [278]. Second-level allogeneic rejection may be mediated by the presence of class II HLA on the CAR-T cells membrane. Indeed, activated human T cells express the MHC class II molecules DR, DP, and DQ, on their cellular membrane, that are regulated by MHC class II trans-activators (CIITA). Although the function of MHC class II molecules on T cells remains controversial [279], it is possible that they may induce allogeneic rejection through the recognition of CD4 + T cells. This problem could be circumvented by genetic manipulation of transcription factor regulators CIITA and X [280]. Allogeneic anti-CD19 CAR T cells with a triple knockout of TCR, class I, and class II HLA outperform double knockout cells and showed good persistence in a study on a model of mouse tumor with antitumor activity but no GVHD [281]. Other cells that may mediate allogeneic responses are NK cells [282], but NKs are functionally impaired in some tumors, especially those of blood origin [283]. Expression or overexpression of inhibitory ligands could be a potential approach to prevent NK allo-rejection mediated by NK cells, HLA-E, or G ligands [284, 285] or Siglec 7/9 ligands [286] is one of the most promising choices. Finally, new workarounds are developed for the rejection of CAR-T cells. A promising solution is the latest generation of CARs that mediate the elimination of activated host NK cells and T cells through the extracellular expression of 4-1BB ligands in combination with intracellular CD3z signaling molecules [287].

**Fig. 8:**
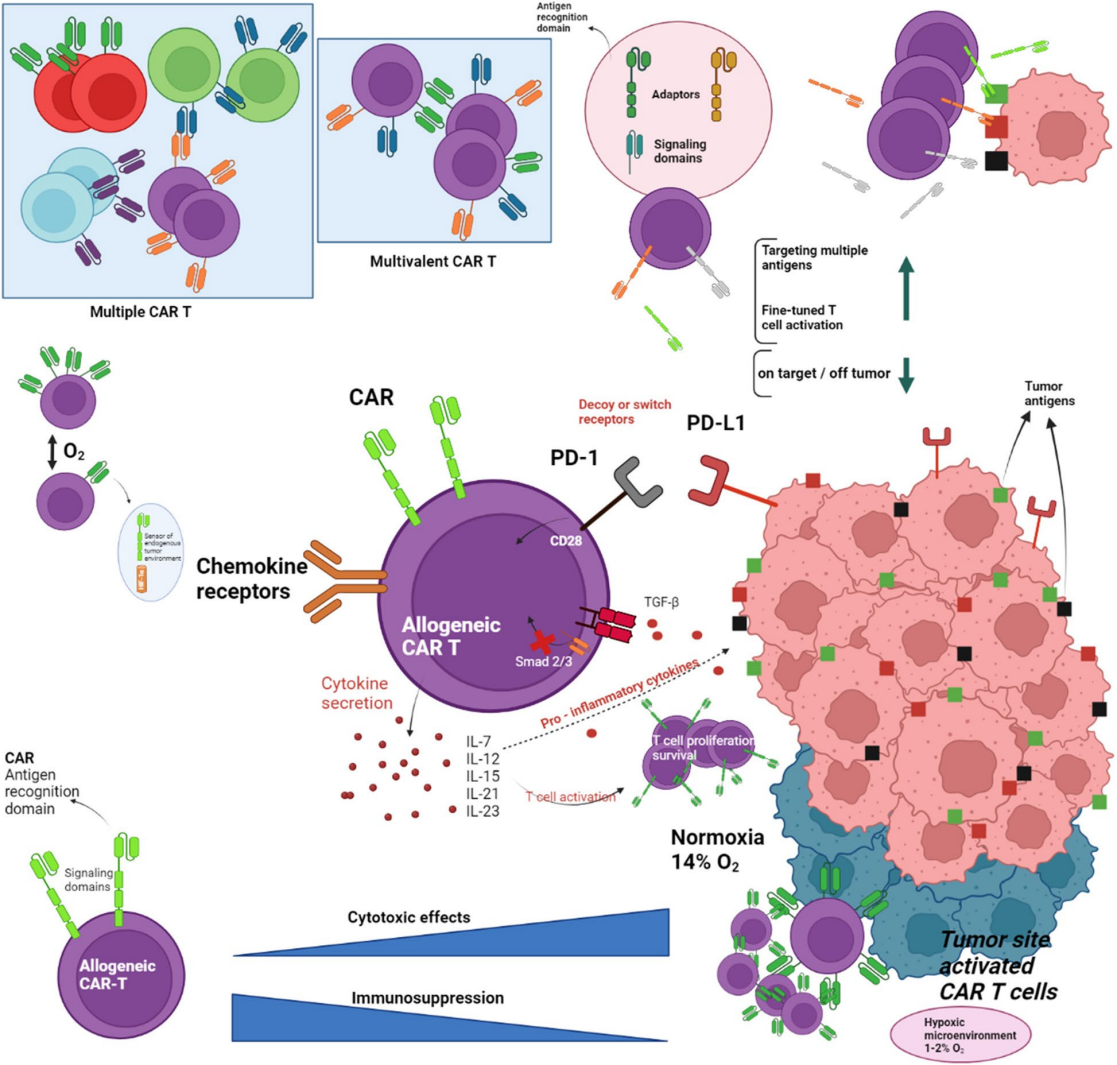
Allogeneic CAR-T cells provide a versatile platform for attacking GBMs and their environment. GBM heterogeneity requires a multi-target approach. This can be achieved by allogeneic CAR-T cells using multiple CAR-T cell mixtures, multivalent CAR-T cells, or modular CAR-T combined with multiple adapters. To overcome the immunosuppressive TME of GBM, different strategies can be employed to develop allogeneic CAR-T cells. Secretion of proinflammatory cytokines (IL-7, IL-12, IL-15, IL-21, IL-23, etc.), expression of lock or switch receptors (to convert immunosuppressive signals into activating signals), expression of chemokine receptors (to convert CAR-T targeting cells to tumor sites), and generation of locally activated CAR-T cells (e.g., hypoxia-induced CAR-T cells)

Loss of antigen is a common mechanism of tumor resistance to CAR-T cell therapy [288] and a major cause of recurrence in GBM [236] and hematological malignancies [289] as well as in preclinical models of solid tumors [290]. An interesting method to overcome antigen flight is the application of a “universal” modular CAR design. Here, a scFv recognizing a target antigen is fused to a soluble intermediate molecule (or adapter) to which a construct containing activation signals that are expressed by T cells can be attached (Fig. 8).

These CARs are designed based on the antibody-Fc receptor, streptavidin–biotin interactions, scFv against specific tags, or other possible combinations [291, 292]. Two of the most popular universal modular CARs are the Universal CAR (UniCAR) and the Shared Universal and Programmable (SUPRA) CAR. The SUPRA CAR consists of a receptor containing a leucine zipper on T cells and another scFv with a leucine zipper adapter molecule that targets a specific antigen [293]. The UniCAR system also has two components: the first one is a CAR-T cell expressing a CAR against the peptide E5B9 derived from core antigen La-SS/B and the other is a so-called targeting module from E5B9 peptide. A tumor-specific antigen-binding domain usually is fused to scFv [294]. The UniCAR system could also target multiple antigens and combine various signaling domains to provide an on/off switch that improves control of activation of CAR T cells [294–296]. In general, adapter molecules allow it to control CAR T cell activity by target selection, addressing one or more of these targets sequentially or simultaneously. Furthermore, effector activity could be individually activated or deactivated for each target by removing or adding soluble adapters without CAR-T cell depletion. Universal modular CAR-T cells, therefore, offer an opportunity to target several tumor antigens with lower toxicity. Along with these desirable properties, adjusting adapter doses could mitigate potential complications of modular CAR-T cells [297]. However, modular CAR-T cells still have some drawbacks related to the exogenous nature of the adapter molecules that can produce neutralizing antibodies within the host. Moreover, each new adapter may require clinical validation, manufacturing development, and approval from regulators for efficacy and safety [298]. Universal standard and modular CARs can be combined to obtain a ready-to-use “fully universal” CAR for modifying target specificity while allowing fine-tuned control. This approach may be particularly suitable for a solid tumor like GBM, that has a high heterogeneity.

### Oncolytic virus in GBM therapy

The concept of viral therapy for malignant tumors was first reported in a case report published in 1912 in which a female with cervical cancer experienced regression of tumor regression being vaccinated against an attenuated rabies virus. Since that time, case studies have reported spontaneous recovery in patients treated with the virus, particularly in leukemia and lymphoma cancers [299, 300]. Nevertheless, concerns about significant side effects and the development of chemotherapy stalled early advances in oncolytic virus therapy [301]. Its potential was reassessed by the late twentieth century, with advances in viral molecular biology and the advent of reverse genetics systems that enabled viral engineering [302]. GBM has been proposed because the tumor is confined to the brain, has no distant metastases, and post-mitotic cells primarily surround growth, allowing the application of viruses that require a replication-active cell cycle. In particular, it is suitable for oncolytic virus (OV) therapy [303]. Currently, immunotherapy in the treatment of GBM, with oncolytic virus, has been promisingly considered and it consists of two groups: (a) OVs with the replication ability, which are selectively replicated in infected cancer cells and suppress tumor cells. (b) Specific OVs are now genetically engineered against pathogen-specific receptors expressed on tumor cells and thus can replicate efficiently and selectively. In addition, viral vectors with replication defects are used as a means to transfer therapeutic genes. Viral infection and amplification ultimately trigger the host’s immune response against tumors and eliminate cancer cells. More than 20 so far, such as adenovirus (Ad) [304], herpes simplex virus type 1 (HSV-1) [305, 306], measles virus (MV) [307], reovirus [308], poliovirus [309], and Newcastle Oncolytic virus candidate disease viruses [310] are being tested in clinical studies as anti-GBM therapies. In addition, new developments in OV delivery techniques are also underway to overcome the limitations caused by the BBB. PVSRIPO, a live attenuated poliovirus type 1 vaccine, replaces the corresponding internal ribosomal entry site with that of human rhinovirus type 2 to limit neurotoxicity. PVSRIPO targets GBM via CD155, which is a high-affinity ligand for immunoglobulins and T cell immunoreceptors with immunoreceptor tyrosine-based inhibitory motif domains that are hugely overexpressed on malignant cells. A phase I clinical trial confirmed the absence of neurotoxic potential with intratumoral IBD of PVSRIPO in cases with recurrent GBM and FDA gave it breakthrough therapy designation in May 2016. Relatively, PVSRIPO immunotherapy was higher than in previous treatment periods at 24 and 36 month controls. Based on phase I results, a randomized phase II clinical trial on PVSRIPO alone or its combination with single-cycle lomustine in cases with relapsed GBM is ongoing. The therapeutic efficacy of this new treatment in GBM patients is awaited. The pace of clinical activities has accelerated significantly [311], since the first time that viral engineering to oncolytic HSV in a murine glioblastoma model is used [312], with several completed or ongoing studies being conducted. In addition, several studies have used genetically engineered oncolytic adenoviruses combined with immune checkpoint inhibition or standard therapy. Phase I and II studies are currently being performed, currently in GBM patients and are expected to yield positive results. Adenoviruses have also been modified into an adenoviral vector, agratimagene besadenovec (AdV-tk), containing the HSV thymidine kinase gene, which is then modified with antiviral agents like valacyclovir, which act as toxic nucleotide analogs that can eliminate tumor cells. Herpes drug prodrugs have also been modified [313]. This approach, known as gene-mediated cytotoxic immunotherapy, proved safe in a clinical phase Ib study in new-onset malignant gliomas [314]. A phase II trial was subsequently conducted and showed a significant increase in malignant glioma survival in association with AdV-tk-based therapy [313]. Although clinical trials have demonstrated the efficacy and safety of OV therapy in GBM, few of them have progressed to phase III trials. A phase III ASPECT study evaluated a gene therapy using adenovirus with Citimagen Seradenovec followed by intravenous injection of ganciclovir in cases with new onset resectable GBM. The safety and efficacy of internal administration were evaluated. ASPECT did not find significant effects on the OS [315]. Recently, a phase III clinical trial with Toca511 and Toca FC was stopped for unknown reasons. Toca 511 consists of a purified retroviral replicating vector which encodes a modulated yeast cytosine deaminase (CD) gene. This gene converts 5-flucytosine (5-FC) to the anticancer drug 5-FU in Toca 511 vector-infected tumor cells. In particular, several phase III studies combined cancer immunotherapy and OV have shown clinical potential for various cancer types [316]. Oncolytic virus therapy in GBM remains promising approach and may affect the patient care future. Recent investigations have found that the Zika virus (ZIKV) exhibits oncolytic activity against GSCs, which suggests that the development of ZIKV may be a therapeutic approach for glioblastoma [317–319]. ZIKV could selectively infect and destroy GSCs compared to normal neurons, making it a potential treatment choice for GBM. Of note, even though the overall safety of OV use has been shown by preclinical and clinical studies, modest clinical efficacy still lives up to the preclinical promise obtained in laboratory studies.

### Vaccination for GBM therapy

Cancer vaccine as a promising approach has offered both curative and preventive potential [320, 321]. In GBM, cancer vaccines target tumor-specific antigens and aim to induce the development of an immune response against the tumor. Because antigens specific to GBM are rare, the targets of GBM antigens are mostly tumor-associated antigens, which leads to limited patient participation. Already, only a few vaccine-based approaches have reached clinical phase III in GBM cases, and many others are in the early stages of clinical investigations. The best-studied tumor-associated antigen is EGFRvIII. It is a constitutively active mutant form of EGFR, 25–30% of GBM [322]. Lindopepimto, a peptide vaccine that targets EGFRvIII, has been studied in several clinical investigations. In three uncontrolled phase II trials, Lindopepimto vaccination in patients with GBM who underwent radiochemotherapy and gross-total resection suggested a 24-month improvement in median survival compared with historical controls [323, 324]. Based on these promising results, an international phase III clinical trial ACT IV was performed to further evaluate the efficacy of lindopepimto in recently diagnosed subjects with EGFRvIII-positive GBM. Despite the patient’s robust immune response against EGFRvIII, the primary analysis of the results showed a survival benefit in patients with minimal residual disease who were injected with lindopepimto in combination with TMZ compared to those who were injected with TMZ alone [325]. Notably, spontaneous antigen loss was observed in both treatment and control groups, raising questions about the application of immunotherapy to target single-tumor antigens with heterogeneous expression in tumors [325]. Recent research from a randomized, double-blind phase II trial in a small group of recurrent EGFRvIII-positive GBM patients showed favorable results with lindopepimto plus standard bevacizumab compared with bevacizumab alone [326]. In conclusion, the positive findings of lindopepimto in recurrent GBM in ReACT and the negative findings of ACT IV in recently diagnosed GBM suggest the need for further clinical studies using combined therapeutic strategies such as angiogenesis inhibition and immunotherapy. ICT107 is a six-synthetic peptide-induced DC vaccine specifically developed for GBM, already in phase III clinical trials. A phase I trial showed the safety of ICT-107 and demonstrated benefits for HLA-A2-positive patients [327]. A phase II study suggested potential therapeutic activity for ICT-107 in HLA-A2-positive cases. Given the encouraging results in preclinical studies and early clinical investigations [328], a phase III trial on utilizing DCVax-L in patients with newly diagnosed GBM was conducted. In this study, the mean OS for the entire intent-to-treat population was 23.1 months, which was more than the mean OS of 15–17 months in previous trials and clinical practice [329]. Nevertheless, this process was later discontinued for unknown reasons. In summary, the results of current clinical trials of vaccines against GBM are disappointing. Lack of antigens specific for GBM and high tumor heterogeneity make challenges for vaccine therapy in GBM patients, but recent advances in next-generation sequencing and new bioinformatics tools have resulted in the discovery of tumor-derived somatic mutations. It is now possible to systematically discover tumor neoantigens that it is therefore tumor-specific [321, 330]. Because neoantigens are extremely specific to individual patients, neoantigen-targeted tumor vaccines efficiently induce novel responses by T cell against neoantigens, enabling precise personalized therapy. Early investigations of personalized neoantigen-based vaccines have shown preliminary evidence of strong tumor-specific immunogenicity and antitumor activity in cases with high-risk melanoma and other cancers [330]. Based on these promising results, a phase I/Ib trial of a personalized neoantigen vaccine was conducted in 10 patients with recently diagnosed MGMT unmethylated GBM after conventional radiation therapy and surgical resection. Patients who did not receive dexamethasone produced circulating multifunctional neoantigen-specific CD4 + and CD8 + T cell responses that were enriched for the memory phenotype and showed increased numbers of T cells infiltrating tumors [331]. Although intratumoral and systemic immune responses against neoantigen were generated after vaccination, all patients experienced recurrence of tumors and eventually died because of progressive disease. This suggests that induced responses by T cells still overcome significant challenges to generate clinically relevant activity against tumor, including tumor-intrinsic anti-tumor activity. Given that neoantigen-targeted vaccines can positively alter the immune environment in glioblastoma, it may be beneficial to combine vaccination with other therapies like immune checkpoint blockade.

## Role of ultrasound for GBM therapy

Despite gradual progress in treating GBM, little new and existing drug therapy has been developed for recurrent GBM [332]. The last agent that significantly improved OS in GBM was TMZ, introduced around 2 decades ago [333]. After years of development, a humanized monoclonal antibody called bevacizumab, which is a vascular endothelial growth factor (VEGF) inhibitor approved by FD for cases with recurrent GBM even without completing a randomized phase III trial. This makes bevacizumab the third treatment for GBM that is approved by the FDA [334]. Subsequently, bevacizumab was investigated in two large randomized phase III clinical trials [335, 336]. Despite impressive median progression-free survival (PFS) in both investigations, utilizing bevacizumab as a first-line therapy did not boost OS in glioblastoma patients. Correspondingly, a systematic analysis revealed that concomitant bevacizumab in newly diagnosed GBM was profitable in prolonging median PFS but not OS [135]. Therefore, innovative therapeutic approaches are needed to improve ultimate outcomes for glioblastoma patients. One of the main limitations of novel GBM treatments is due in part to the inefficiency in the delivery of drugs across the blood–brain barrier. The blood–brain barrier consists of endothelial cells lining the brain's microvasculature and poses special challenges for drug delivery [337]. Recently, focused ultrasound as a solution for this problem has made this technique a viable new option for CNS targeting [338]. Preclinical studies have shown that low-intensity pulsed ultrasound improves levels of systemically administered drug therapy in the parenchyma of the brain in animal models which resulted in prolonged survival in preclinical models of GBM [339–343]. After decades of preclinical research, centralized ultrasound has recently been applied to clinical trials of GBM [344]. In 2016, the first human single-center, single-arm study was started to investigate the feasibility and safety of repeated pulse ultrasound in patients with recurrent GBM [337]. Results of the study suggested that focused ultrasound as a new technique to treat patients with GBM is safe and less burdensome [333, 337]. More importantly, the additional treatment of pulsed ultrasound reported in this study can be enhanced and combined with further treatments to improve the penetration of the drug in GBM patients [333]. A prospective, open-label, single-arm, study was performed to investigate the effect of continuous magnetic resonance-guided focused ultrasound (MRgFUS) combined with adjuvant TMZ in GBM patients. This first human proof-of-concept study demonstrated that MRgFUS enhances the signals of circulating brain-derived biomarkers and provides data on the feasibility of focused ultrasound frames for liquid biopsy in patients with neuro tumors [345]. Temporary opening of the BBB in tumors by non-invasive low-intensity MRgFUS and systemic chemotherapy is safe and feasible [346]. Meanwhile, it is recommended that photodynamic therapy (PDT) be considered in this category [347].

## Role of nucleic acid-based GBM therapy

Oligonucleotide-based therapies offer a wide range of treatment options for cancer. Oligonucleotides can be precisely engineered to have gene sequences that are unique to tumors rather than natural cells, allowing this type of drug to have precise specificity during treatment. One such agent recognizes the insulin-like growth factor-1 (IGF-1) receptor as a target, an oncogenic receptor constitutively overexpressed in GBMs that confers tumor cell resistance to radiation and apoptosis; IMV-001, a siRNA antisense oligonucleotide [348]. This drug in combination with autologous GBM cells was studied in a phase I trial of 33 newly diagnosed GBM patients [349]. Analysis revealed a mean overall PFS of 11.6 months and 17.1 months for patients who received the highest dose. The median overall survival of patients eligible for the protocol was determined to be 38.2 months [349]. In this way, a phase I study using two engineered DNA plasmids combined with the anti-PD-1 antibody semiplimab for treatment is a plasmid encoding hTERT, PSMA, and WT-1 and INO-9012 encodes IL-12 and is delivered by electroporation to ensure its uptake [350]. Importantly, hTERT, the human telomerase reverse transcriptase gene, is frequently mutated and hyper-activated in GBM cells [351]. WT-1 or Wilms tumor 1 is an overexpressed oncoprotein in GBM which is considered a tumor-associated antigen [352, 353]. PSMA, a prostate-specific membrane antigen, is found on new blood vessels within the GBM structure [354]. This grouping and semiplimab are thought to induce the response of the immune system to these specific antigens that are not expressed in healthy cells in normal conditions. The recent success of an mRNA vaccine against COVID-19 highlights the potential of mRNA technology in immunotherapy for cancer [355]. Much remains to be found out about these therapeutics and their potential in vivo efficacy and/or complications in humans. Numerous clinical trials are likely to begin over the next decade as more and more biotech companies acquire and perfect their capabilities to manufacture these compounds. Various immunotherapeutic approaches have shown promising findings in preclinical studies, but many have not produced effective or sustained responses in the clinical setting. Several interventions focused on combined immunotherapy could be applied to enhance the response of the immune system. These approaches in GBM have been discussed elsewhere [356]. The principles and practices of combined immunotherapy in general oncology have also been extensively reviewed in recent research [357]. Below are some important examples of preclinical studies and active clinical investigations in the area of ​​ immunotherapy and GBM. This strategy showed higher survival rates and responses of T cell memory upon tumor reclamation [358]. A combination of anti-CTLA-4 and anti-PD-1 antibodies was used with IL-12-expressing oncolytic herpes simplex virus to treat the GSC-based mouse model of GBM [359]. Several clinical studies investigating combination immunotherapies are currently underway. In a TEM-GBM study, hematopoietic stem cells are transduced with a lentivirus that directs expression of IFN-α in Tie-2-positive monocytes [360]. In addition to therapies using immunomodulatory cytokines, novel cell-based therapies are combined with gene therapy viral administration. No dose-limiting toxicity was reported in this study [360]. In the future, autologous glioma cell lysates and other personalized medicine strategies will be applied to target tumors at an individual tumor-specific level. As this technology advances, the ability of modern oncology to extend the survival of patients with glioma will increase accordingly. In addition, advances in oligonucleotide and other nucleic acid-based therapeutics, including prevalent mRNA vaccine technology in the current pandemic’s coronavirus vaccines, will open up opportunities for personalized medicine. With the rise of immunotherapy and several new agents, physicians must exercise great caution in evaluating all available clinical studies and weighing potential benefits compared to conventional radiation and chemotherapy. The aim is to identify patients who are interested and suitable for participation in clinical studies. Key factors influencing clinical trial participation include the presence of American Society of Clinical Oncology (ASCO)-related comorbidities. The primary responsibility for recruiting patients interested in clinical trials rests with clinicians. Knowing about the various clinical trial options, their possible side effects, and the centers that offer them, can help physicians help patients make more informed decisions about treatment.

## Role of MEK in GBM therapy

Extracellular signal-activated kinases (MEKs) can be classified into two distinct forms: in the same vein, important genes such as MEK1 and MEK2. MEK, which is a downstream kinase and part of the RAS cascade, is responsible for signaling related to important cellular functions such as cell proliferation, survival, and differentiation [361, 362]. Dysregulation of the Ras-Raf-MEK-ERK signaling pathway is therefore common in oncogenesis. It is activated by both growth factors and changes in several proteins involved in this cascade. Mutation prevalence decreases along the pathway, making it most common in RAS (22%) and BRAF (7%), least common in MEK (less than 1%), and less common in ERK (very rare). At the initiation of the Ras-Raf-MEK-ERK cascade mechanism, growth factors bind to tyrosine kinase receptors (TKR). Epidermal growth factor receptor (EGFR) leads to receptor activation and subsequent activation of Ras small GTPase. This leads to the formation of Ras-GTP and subsequent activation of Ras. The Raf serine/threonine kinase then becomes a downstream effector target of Ras, and finally, the activated Raf protein, upon phosphorylation, activates MEK1 and MEK2 hyperactivity of this cascade is significantly correlated with tumor cell proliferation and cancer progression. Therefore, molecular therapeutics targeting the Ras-Raf-MEK-ERK signaling pathway are being actively developed [363]. Dysregulation of signaling through the Ras signaling pathway has been observed in approximately 90% of GBM [364]. MEK1/2 is involved not only in tumorigenesis but also in the inhibition of apoptosis. Therefore, MEK1/2 inhibitors are a suitable treatment option [365]. There is evidence that blocking MEK signaling in GBM is associated with antiproliferative effects by blocking cell division and reducing the percentage of Ki67-positive cells. As a MEK1/2 inhibitor, trametinib has been shown to inhibit the Ras-Raf-MEK-ERK pathway and block its downstream extracellular kinases. In addition, it can limit the proliferation, migration, and invasion of GBM. Trametinib monotherapy has been approved by the FDA for the treatment of melanoma to date [366, 367]. In addition to trametinib, other MEK inhibitors such as cobimetinib are also considered novel treatments for GBM. They are generally well tolerated and their efficacy can be judged by a reduction in tumor size [368]. Phosphatidylinositol-3-kinases (PI3Ks) represent a family of lipid kinases that are activated by numerous receptor tyrosine kinases. The mammalian target of rapamycin (mTOR) is a serine/threonine kinase that can sense and transduce signals from a variety of stimuli and belongs to the family of PI3K-related protein kinases. The PI3K/mTOR signaling pathway plays a key role in several key cellular functions such as cell metabolism, growth, survival angiogenesis, and motility [369]. In addition, aberrant activity of this signaling pathway has also been implicated in the development of GBM. PI3K mutations are found in 1 in 4 of GBM patients [370]. The most common mechanisms associated with the overactivation of the PI3K signaling pathway include mutations in the catalytic α subunit of phosphatidylinositol-4, 5-bisphosphate-3-kinase (PIK3CA), phosphatase, and tensin homologs (PTEN). Loss of function and epidermal growth factor receptor (EGFR) gene [371] are important in the mechanism of the PI3K/Akt/mTOR signaling pathway. PI3K migrates to the plasma membrane and catalyzes the production of phosphatidylinositol-3,4,5-triphosphate (PIP3), which in turn activates phosphoinositide-dependent serine/threonine kinase 1 (PDK1) or Akt, which the result leads to the inhibition of apoptosis. Activated Akt then activates mTOR (a downstream target of PI3K) by mediating protein synthesis [372]. Rapid tumor growth and multidrug resistance result from over-activation of the PI3K/Akt pathway in GBM. Consequently, inhibition of PI3K alone or in combination with other targets may lead to cellular apoptosis and slow progression of GBM. PI3K inhibitors include pan-PI3K inhibitors. Furthermore, PI3Ks can be divided into three classes, and Class I PI3Ks consist of a catalytic subunit and a regulatory subunit [373]. Pan-PI3K inhibitors and isoform-selective PI3K inhibitors suppress the activity of p110 catalytic isoforms, whereas dual PI3K/mTOR inhibitors act on both p110 and mTOR complex 1/2. Over 50 PI3K inhibitors have been discovered in cancer therapy, but only a few are currently being tested in clinical trials [374]. PI3K inhibitors that have been shown to have the ability to enter the brain to date include NVP-BEZ235, XL765, GDC-0084, and PQR 309. The development of targeted therapies that focus on the PI3K signaling pathway will have wide-ranging and clinically important applications.

## Role of FGFR in GBM therapy

Key regulators of tissue growth, metabolism, differentiation, and repair are fibroblast growth factors (FGFs). FGF signaling is induced by acting through tyrosine kinase receptors known as fibroblast growth factor receptors (FGFRs). Four transmembrane receptors can be distinguished in the FGFR family. FGFR1-4 [375]. FGF–FGFR induces cell signaling pathways such as RAC/JNK, RAS-MAPK (both related to cell proliferation), and PI3K/AKT. Furthermore, gene expression analysis showed a correlation between somatic FGFR mutations and GBM progression [376]. With a prevalence of approximately 6%, FGFR genomic alterations are less common in GBM [377]. The FGFR3-TACC3 fusion has been identified as the most common FGFR alteration underlying IDH wild-type GBM [378]. FGFR1 has been implicated in the development of cytotoxicity and resistance to hormone therapy in various types of tumors [379, 380]. FGFR1 can also modulate the tumor microenvironment and angiogenic response, resulting in decreased GBM radiosensitivity [381]. FGFR1 levels increase with tumor progression, whereas FGFR2 expression steadily decreases with increasing GBM grade [382]. Decreased expression of FGFR2 is closely associated with poor patient prognosis and worse outcomes [383]. Higher levels of FGFR2, as measured by Ki-67 core antigen expression, may correlate with decreased proliferation. The fusion of FGFR3 and TACC3 genes, which occurs in 3% of GBM, leads to the formation of oncogenic FGFR3 [376]. In these cases, increased oxidative phosphorylation and mitochondrial activity are observed, which play a key role in GBM [384]. Recent studies have shown that FGFR4 contributes to GBM cell viability, adhesion, migration, and clonogenicity. Furthermore, FGFR4 has been reported as a predictor of shorter survival in patients with this type of brain tumor [385]. Considering the above points, FGFR inhibition is a potential therapeutic target in GBM patients [386]. Selective inhibitors like nintedanib and pemigatinib are already being tested in clinical studies.

## Role of VEGF in GBM therapy

The vascular endothelial growth factor (VEGF) family includes proteins associated with specific receptors such as neuropilin-1, neuropilin-2, VEGFR-1, VEGFR-2, and VEGFR-3 [387]. VEGF is a prognostic angiogenesis marker that has been shown to play an important role in the pathobiology of GBM [388]. Necrotic and hypoxic conditions activate GBM cells and induce the pro-angiogenic factors to release like VEGF [389]. VEGF is generated by GBM tumor cells, stromal cells, and inflammatory cells and stimulates VEGF receptors, leading to endothelial cell proliferation, migration, and survival. This contributes significantly to increased tumor perfusion and increased interstitial pressure. This leads to the blood–brain barrier loss and the development of mass-effect vasogenic edema, a major etiology of morbidity in GBM patients [390]. GBM is one of the most vascularized solid tumors. It is characterized by strong vascular proliferation, leading to the formation of dilated, tortuous, impermeable, and hyperpermeable vessels. Abnormal and dysfunctional vasculature can result in limited delivery of chemotherapeutic agents to the tumor mass. Malignant vasculature is closely associated with the development of the GBM. Therefore, the degree of angiogenesis strongly correlates with prognosis. Antiangiogenic drugs are currently being investigated as potentially potent anti-GBM therapies [389, 391]. Bevacizumab is a humanized monoclonal antibody that targets VEGF-A. The only FDA-approved VEGF inhibitor therapy for recurrent GBM was Bevacizumab monotherapy [392]. Anti-angiogenic therapies evaluated in clinical trials offer complementary or alternative options to conventional treatment of GBM. It has been considered in the treatment of GBM [393].

## Role of pharmaceutical applications for GBM therapy

The main potential antineoplastic agents are important in GBM therapy. These include BRAF inhibitors (dabrafenib and vemurafenib), MEK inhibitors (cobimetinib and trametinib), PI3K inhibitors (paxalisib), FGFR inhibitors (pemigatinib and nintedanib), mTOR inhibitors (everolimus), VEGF inhibitors (Bevacizumab and Vercept), and VEGFR inhibitors (pazopanib, nintedanib, sorafenib, lenvatinib, sunitinib, regorafenib, and apatinib). Based on recent investigations, drug treatment efficacy as measured by progression-free and overall survival was compared in patients who were treated with selected targeted agents.

## Conclusions

Cancer immunotherapy is considered one of the most new and practical types of cancer therapy particularly for GBM. Notably, this molecular and practical structure of GBM induced scientists to consider strongly to particular and alternative procedures, for the most impressive result in therapy and with fewer side effects. In this account, immune checkpoints in GBM therapy pursue to exceed the tolerance of induced tumors by the reversal of T cell rebuilding and exhaustion of anti-tumor immunity, and many different clinical investigations have recently been done for patients with brain tumors, particularly GBM. The progression of an applied and effective vaccine for GBM is one of the most challenging discoveries for researchers. Notably, most novel cancer immunotherapies are centralized on the significance of all cytotoxic T cells. Predictably, it can be concluded to underestimate the importance of innate immune structures in the microenvironment of the tumor, comprising TAMs. Tumors strongly have adaptive properties and conserve abundant non-cancerous cells. Consequently, concurrent therapies including multiple attitudes that synchronously target tumor cells, T cells, and TAMs must be investigated. In this account, blocking TAM-mediated immunosuppression has been shown to hold great promise in increasing the efficacy of gene therapy-mediated immunotherapies for GBM. In the treatment of GBM, CAR T cell therapies, especially second and third-generation CAR T cell therapies, have achieved promising preclinical efficacy in prolonging the survival of the patient. Meaningly, CAR T cell therapies are transforming the treatment of hematological malignancies and have the potential to do the same for solid tumors. However, despite some evidence of anti-tumor effects, CAR T cell therapies against GBM have not yet demonstrated their efficacy as a viable and impactful treatment option. A large portion of immunosuppressive cells (like Tregs, TAMs, and MDSCs) enter the GBM microenvironment, upregulating several immune checkpoints (e.g., PD-1, Tim-3, CTLA-4, and IDO-1) as well as immunosuppressive ligands (e.g., PD-L1, on GBM and tumor-infiltrating myeloid cells) and GBM tumor antigen masking. These factors lead to the GBM’s immunosuppressive environment and cause inhibition and dysfunction of the proliferation of infiltrating T cells. Thus, reducing or eliminating immunosuppressive cell infiltration and increasing the number and activity of effector T cells are key to the success of GBM immunotherapy. In summary, degradation of the immunosuppressive environment, activation of tumor killer cells in the tumor microenvironment, and release of tumor antigens are the most promising immunotherapeutic strategies for the treatment of tumors. GBM therapy and a combination of two or three strategies will inhibit GBM tumor growth and improve treatment.

## Data Availability

Not applicable.

## References

[1] Davis FG, Freels S, Grutsch J, Barlas S, Brem S. Survival rates in patients with primary malignant brain tumors stratified by patient age and tumor histological type: an analysis based on Surveillance, Epidemiology, and End Results (SEER) data, 1973–1991. J Neurosurg. 1998;88(1):1–10. 9420066 10.3171/jns.1998.88.1.0001

[2] Hanahan D, Weinberg RA. Hallmarks of cancer: the next generation. cell. 2011;144(5):646–74. 10.1016/j.cell.2011.02.013 21376230

[3] Stupp R, Hegi ME, Mason WP, Van Den Bent MJ, Taphoorn MJ, Janzer RC, et al. Effects of radiotherapy with concomitant and adjuvant temozolomide versus radiotherapy alone on survival in glioblastoma in a randomised phase III study: 5-year analysis of the EORTC-NCIC trial. Lancet Oncol. 2009;10(5):459–66. 19269895 10.1016/S1470-2045(09)70025-7

[4] Stupp R, Taillibert S, Kanner A, Read W, Steinberg DM, Lhermitte B, et al. Effect of tumor-treating fields plus maintenance temozolomide vs maintenance temozolomide alone on survival in patients with glioblastoma: a randomized clinical trial. JAMA. 2017;318(23):2306–16. 29260225 10.1001/jama.2017.18718 PMC5820703

[5] Verhaak RG, Hoadley KA, Purdom E, Wang V, Qi Y, Wilkerson MD, et al. Integrated genomic analysis identifies clinically relevant subtypes of glioblastoma characterized by abnormalities in PDGFRA, IDH1, EGFR, and NF1. Cancer Cell. 2010;17(1):98–110. 20129251 10.1016/j.ccr.2009.12.020 PMC2818769

[6] Noushmehr H, Weisenberger DJ, Diefes K, Phillips HS, Pujara K, Berman BP, et al. Identification of a CpG island methylator phenotype that defines a distinct subgroup of glioma. Cancer Cell. 2010;17(5):510–22. 20399149 10.1016/j.ccr.2010.03.017 PMC2872684

[7] Cuddapah VA, Robel S, Watkins S, Sontheimer H. A neurocentric perspective on glioma invasion. Nat Rev Neurosci. 2014;15(7):455–65. 24946761 10.1038/nrn3765 PMC5304245

[8] Vitorino P, Meyer T. Modular control of endothelial sheet migration. Genes Dev. 2008;22(23):3268–81. 19056882 10.1101/gad.1725808 PMC2600767

[9] Hartmann CH, Klein CA. Gene expression profiling of single cells on large-scale oligonucleotide arrays. Nucleic acids research. 2006;34(21):e143-e. 10.1093/nar/gkl740 PMC1635316 17071717

[10] Claes A, Idema AJ, Wesseling P. Diffuse glioma growth: a guerilla war. Acta Neuropathol. 2007;114:443–58. 17805551 10.1007/s00401-007-0293-7 PMC2039798

[11] Friedl P, Wolf K. Tumour-cell invasion and migration: diversity and escape mechanisms. Nat Rev Cancer. 2003;3(5):362–74. 12724734 10.1038/nrc1075

[12] Wolf K, Friedl P. Molecular mechanisms of cancer cell invasion and plasticity. Br J Dermatol. 2006;154(s1):11–5. 16712711 10.1111/j.1365-2133.2006.07231.x

[13] Kim J, Lee I-H, Cho HJ, Park C-K, Jung Y-S, Kim Y, et al. Spatiotemporal evolution of the primary glioblastoma genome. Cancer Cell. 2015;28(3):318–28. 26373279 10.1016/j.ccell.2015.07.013

[14] Hou LC, Veeravagu A, Hsu AR, Victor C. Recurrent glioblastoma multiforme: a review of natural history and management options. Neurosurg Focus. 2006;20(4):E3. 10.3171/foc.2006.20.4.2 16709036

[15] Friedl P, Gilmour D. Collective cell migration in morphogenesis, regeneration and cancer. Nat Rev Mol Cell Biol. 2009;10(7):445–57. 19546857 10.1038/nrm2720

[16] Friedl P, Locker J, Sahai E, Segall JE. Classifying collective cancer cell invasion. Nat Cell Biol. 2012;14(8):777–83. 22854810 10.1038/ncb2548

[17] Cheung KJ, Gabrielson E, Werb Z, Ewald AJ. Collective invasion in breast cancer requires a conserved basal epithelial program. Cell. 2013;155(7):1639–51. 24332913 10.1016/j.cell.2013.11.029 PMC3941206

[18] Alieva M, Leidgens V, Riemenschneider MJ, Klein CA, Hau P, van Rheenen J. Intravital imaging of glioma border morphology reveals distinctive cellular dynamics and contribution to tumor cell invasion. Sci Rep. 2019;9(1):2054. 30765850 10.1038/s41598-019-38625-4 PMC6375955

[19] Friedl P, Alexander S. Cancer invasion and the microenvironment: plasticity and reciprocity. Cell. 2011;147(5):992–1009. 22118458 10.1016/j.cell.2011.11.016

[20] Nevo I, Woolard K, Cam M, Li A, Webster JD, Kotliarov Y, et al. Identification of molecular pathways facilitating glioma cell invasion in situ. PLoS ONE. 2014;9(11): e111783. 25365423 10.1371/journal.pone.0111783 PMC4218815

[21] Engler JR, Robinson AE, Smirnov I, Hodgson JG, Berger MS, Gupta N, et al. Increased microglia/macrophage gene expression in a subset of adult and pediatric astrocytomas. 2012. 10.1371/journal.pone.0043339 PMC3425586 22937035

[22] Wang Q, Hu B, Hu X, Kim H, Squatrito M, Scarpace L, et al. Tumor evolution of glioma-intrinsic gene expression subtypes associates with immunological changes in the microenvironment. Cancer cell. 2017;32(1):42–56. e6. 10.1016/j.ccell.2017.06.003 PMC5599156 28697342

[23] Doucette T, Rao G, Rao A, Shen L, Aldape K, Wei J, et al. Immune heterogeneity of glioblastoma subtypes: extrapolation from the cancer genome atlas. Cancer Immunol Res. 2013;1(2):112–22. 24409449 10.1158/2326-6066.CIR-13-0028 PMC3881271

[24] Han S, Ma E, Wang X, Yu C, Dong T, Zhan W, et al. Rescuing defective tumor-infiltrating T-cell proliferation in glioblastoma patients. Oncol Lett. 2016;12(4):2924–9. 27703529 10.3892/ol.2016.4944 PMC5038909

[25] Zhai L, Ladomersky E, Lauing KL, Wu M, Genet M, Gritsina G, et al. Infiltrating T cells increase IDO1 expression in glioblastoma and contribute to decreased patient survival. Clin Cancer Res. 2017;23(21):6650–60. 28751450 10.1158/1078-0432.CCR-17-0120 PMC5850948

[26] Hosseinalizadeh H, Mahmoodpour M, Samadani AA, Roudkenar MH. The immunosuppressive role of indoleamine 2, 3-dioxygenase in glioblastoma: mechanism of action and immunotherapeutic strategies. Med Oncol. 2022;39(9):130. 35716323 10.1007/s12032-022-01724-w PMC9206138

[27] Curiel TJ. Regulatory T cells and treatment of cancer. Curr Opin Immunol. 2008;20(2):241–6. 18508251 10.1016/j.coi.2008.04.008 PMC3319305

[28] Crane CA, Ahn BJ, Han SJ, Parsa AT. Soluble factors secreted by glioblastoma cell lines facilitate recruitment, survival, and expansion of regulatory T cells: implications for immunotherapy. Neuro Oncol. 2012;14(5):584–95. 22406925 10.1093/neuonc/nos014 PMC3337302

[29] Jacobs JF, Idema AJ, Bol KF, Grotenhuis JA, de Vries IJM, Wesseling P, Adema GJ. Prognostic significance and mechanism of Treg infiltration in human brain tumors. J Neuroimmunol. 2010;225(1–2):195–9. 20537408 10.1016/j.jneuroim.2010.05.020

[30] Fecci PE, Mitchell DA, Whitesides JF, Xie W, Friedman AH, Archer GE, et al. Increased regulatory T-cell fraction amidst a diminished CD4 compartment explains cellular immune defects in patients with malignant glioma. Can Res. 2006;66(6):3294–302. 10.1158/0008-5472.CAN-05-3773 16540683

[31] Herting C, Chen Z, Pitter K, Szulzewsky F, Kaffes I, Kaluzova M, et al. Genetic driver mutations define the expression signature and microenvironmental composition of high-grade gliomas. Glia. 2017;65(12):1914–26. 28836293 10.1002/glia.23203 PMC5988206

[32] Hambardzumyan D, Parada LF, Holland EC, Charest A. Genetic modeling of gliomas in mice: new tools to tackle old problems. Glia. 2011;59(8):1155–68. 21305617 10.1002/glia.21142 PMC3619979

[33] Ginhoux F, Greter M, Leboeuf M, Nandi S, See P, Gokhan S, et al. Fate mapping analysis reveals that adult microglia derive from primitive macrophages. Science. 2010;330(6005):841–5. 20966214 10.1126/science.1194637 PMC3719181

[34] Elmore MR, Najafi AR, Koike MA, Dagher NN, Spangenberg EE, Rice RA, et al. Colony-stimulating factor 1 receptor signaling is necessary for microglia viability, unmasking a microglia progenitor cell in the adult brain. Neuron. 2014;82(2):380–97. 24742461 10.1016/j.neuron.2014.02.040 PMC4161285

[35] Kierdorf K, Erny D, Goldmann T, Sander V, Schulz C, Perdiguero EG, et al. Microglia emerge from erythromyeloid precursors via Pu. 1-and Irf8-dependent pathways. Nature neuroscience. 2013;16(3):273–80. 10.1038/nn.3318 23334579

[36] Shi C, Pamer EG. Monocyte recruitment during infection and inflammation. Nat Rev Immunol. 2011;11(11):762–74. 21984070 10.1038/nri3070 PMC3947780

[37] Yona S, Kim K-W, Wolf Y, Mildner A, Varol D, Breker M, et al. Fate mapping reveals origins and dynamics of monocytes and tissue macrophages under homeostasis. Immunity. 2013;38(1):79–91. 23273845 10.1016/j.immuni.2012.12.001 PMC3908543

[38] Gordon S, Taylor PR. Monocyte and macrophage heterogeneity. Nat Rev Immunol. 2005;5(12):953–64. 16322748 10.1038/nri1733

[39] Chen Z, Feng X, Herting CJ, Garcia VA, Nie K, Pong WW, et al. Cellular and molecular identity of tumor-associated macrophages in glioblastoma. Can Res. 2017;77(9):2266–78. 10.1158/0008-5472.CAN-16-2310 PMC5741820 28235764

[40] Dal-Secco D, Wang J, Zeng Z, Kolaczkowska E, Wong CH, Petri B, et al. A dynamic spectrum of monocytes arising from the in situ reprogramming of CCR2+ monocytes at a site of sterile injury. J Exp Med. 2015;212(4):447–56. 25800956 10.1084/jem.20141539 PMC4387291

[41] Shemer A, Jung S, editors. Differential roles of resident microglia and infiltrating monocytes in murine CNS autoimmunity. Seminars in immunopathology; 2015: Springer. 10.1007/s00281-015-0519-z 26240063

[42] London A, Cohen M, Schwartz M. Microglia and monocyte-derived macrophages: functionally distinct populations that act in concert in CNS plasticity and repair. Front Cell Neurosci. 2013;7:34. 23596391 10.3389/fncel.2013.00034 PMC3625831

[43] Ajami B, Bennett JL, Krieger C, McNagny KM, Rossi FM. Infiltrating monocytes trigger EAE progression, but do not contribute to the resident microglia pool. Nat Neurosci. 2011;14(9):1142–9. 21804537 10.1038/nn.2887

[44] Moqrich A. Peripheral pain-sensing neurons: from molecular diversity to functional specialization. Cell Rep. 2014;6(2):245–6. 24484769 10.1016/j.celrep.2014.01.018

[45] Feng X, Szulzewsky F, Yerevanian A, Chen Z, Heinzmann D, Rasmussen RD, et al. Loss of CX3CR1 increases accumulation of inflammatory monocytes and promotes gliomagenesis. Oncotarget. 2015;6(17):15077. 25987130 10.18632/oncotarget.3730 PMC4558137

[46] Gabrilovich DI, Ostrand-Rosenberg S, Bronte V. Coordinated regulation of myeloid cells by tumours. Nat Rev Immunol. 2012;12(4):253–68. 22437938 10.1038/nri3175 PMC3587148

[47] Gordon S. Alternative activation of macrophages. Nat Rev Immunol. 2003;3(1):23–35. 12511873 10.1038/nri978

[48] Ding AH, Nathan CF, Stuehr D. Release of reactive nitrogen intermediates and reactive oxygen intermediates from mouse peritoneal macrophages. Comparison of activating cytokines and evidence for independent production. Journal of immunology (Baltimore, Md: 1950). 1988;141(7):2407–12. 3139757

[49] Martinez FO, Helming L, Gordon S. Alternative activation of macrophages: an immunologic functional perspective. Annu Rev Immunol. 2009;27:451–83. 19105661 10.1146/annurev.immunol.021908.132532

[50] Mosser DM, Edwards JP. Exploring the full spectrum of macrophage activation. Nat Rev Immunol. 2008;8(12):958–69. 19029990 10.1038/nri2448 PMC2724991

[51] Biswas SK, Mantovani A. Macrophage plasticity and interaction with lymphocyte subsets: cancer as a paradigm. Nat Immunol. 2010;11(10):889–96. 20856220 10.1038/ni.1937

[52] Guruharsha K, Kankel MW, Artavanis-Tsakonas S. The Notch signalling system: recent insights into the complexity of a conserved pathway. Nat Rev Genet. 2012;13(9):654–66. 22868267 10.1038/nrg3272 PMC4369923

[53] Andersson ER, Sandberg R, Lendahl U. Notch signaling: simplicity in design, versatility in function. Development. 2011;138(17):3593–612. 21828089 10.1242/dev.063610

[54] Yang X, Klein R, Tian X, Cheng H-T, Kopan R, Shen J. Notch activation induces apoptosis in neural progenitor cells through a p53-dependent pathway. Dev Biol. 2004;269(1):81–94. 15081359 10.1016/j.ydbio.2004.01.014

[55] Sestan N, Artavanis-Tsakonas S, Rakic P. Contact-dependent inhibition of cortical neurite growth mediated by notch signaling. Science. 1999;286(5440):741–6. 10531053 10.1126/science.286.5440.741

[56] Breunig JJ, Silbereis J, Vaccarino FM, Šestan N, Rakic P. Notch regulates cell fate and dendrite morphology of newborn neurons in the postnatal dentate gyrus. Proc Natl Acad Sci. 2007;104(51):20558–63. 18077357 10.1073/pnas.0710156104 PMC2154470

[57] Stump G, Durrer A, Klein A-L, Lütolf S, Suter U, Taylor V. Notch1 and its ligands Delta-like and Jagged are expressed and active in distinct cell populations in the postnatal mouse brain. Mech Dev. 2002;114(1–2):153–9. 12175503 10.1016/s0925-4773(02)00043-6

[58] Irvin DK, Zurcher SD, Nguyen T, Weinmaster G, Kornblum HI. Expression patterns of Notch1, Notch2, and Notch3 suggest multiple functional roles for the Notch-DSL signaling system during brain development. Journal of Comparative Neurology. 2001;436(2):167–81. 11438922

[59] Murphy PA, Lam MT, Wu X, Kim TN, Vartanian SM, Bollen AW, et al. Endothelial Notch4 signaling induces hallmarks of brain arteriovenous malformations in mice. Proc Natl Acad Sci. 2008;105(31):10901–6. 18667694 10.1073/pnas.0802743105 PMC2504798

[60] Irvin DK, Nakano I, Paucar A, Kornblum HI. Patterns of Jagged1, Jagged2, Delta-like 1 and Delta-like 3 expression during late embryonic and postnatal brain development suggest multiple functional roles in progenitors and differentiated cells. J Neurosci Res. 2004;75(3):330–43. 14743446 10.1002/jnr.10843

[61] Yoon K-J, Koo B-K, Im S-K, Jeong H-W, Ghim J, Kwon M-c, et al. Mind bomb 1-expressing intermediate progenitors generate notch signaling to maintain radial glial cells. Neuron. 2008;58(4):519–31. 10.1016/j.neuron.2008.03.018 18498734

[62] Shutter JR, Scully S, Fan W, Richards WG, Kitajewski J, Deblandre GA, et al. Dll4, a novel Notch ligand expressed in arterial endothelium. Genes Dev. 2000;14(11):1313–8. 10837024 PMC316657

[63] Alberi L, Liu S, Wang Y, Badie R, Smith-Hicks C, Wu J, et al. Activity-induced Notch signaling in neurons requires Arc/Arg3. 1 and is essential for synaptic plasticity in hippocampal networks. Neuron. 2011;69(3):437–44. 10.1016/j.neuron.2011.01.004 PMC3056341 21315255

[64] Lavado A, Lagutin OV, Chow LM, Baker SJ, Oliver G. Prox1 is required for granule cell maturation and intermediate progenitor maintenance during brain neurogenesis. PLoS Biol. 2010;8(8): e1000460. 20808958 10.1371/journal.pbio.1000460 PMC2923090

[65] Miller AC, Lyons EL, Herman TG. cis-Inhibition of Notch by endogenous Delta biases the outcome of lateral inhibition. Curr Biol. 2009;19(16):1378–83. 19631544 10.1016/j.cub.2009.06.042 PMC2761761

[66] del Álamo D, Rouault H, Schweisguth F. Mechanism and significance of cis-inhibition in Notch signalling. Curr Biol. 2011;21(1):R40–7. 21215938 10.1016/j.cub.2010.10.034

[67] LeBon L, Lee TV, Sprinzak D, Jafar-Nejad H, Elowitz MB. Fringe proteins modulate Notch-ligand cis and trans interactions to specify signaling states. elife. 2014;3:e02950. 10.7554/eLife.02950 PMC4174579 25255098

[68] Kopan R, Ilagan MXG. The canonical Notch signaling pathway: unfolding the activation mechanism. Cell. 2009;137(2):216–33. 19379690 10.1016/j.cell.2009.03.045 PMC2827930

[69] Penton AL, Leonard LD, Spinner NB, editors. Notch signaling in human development and disease. Seminars in cell & developmental biology; 2012: Elsevier. 10.1016/j.semcdb.2012.01.010 PMC3638987 22306179

[70] Takebe N, Nguyen D, Yang SX. Targeting notch signaling pathway in cancer: clinical development advances and challenges. Pharmacol Ther. 2014;141(2):140–9. 24076266 10.1016/j.pharmthera.2013.09.005 PMC3982918

[71] Ranganathan P, Weaver KL, Capobianco AJ. Notch signalling in solid tumours: a little bit of everything but not all the time. Nat Rev Cancer. 2011;11(5):338–51. 21508972 10.1038/nrc3035

[72] Chen Y, Pang J, Ye L, Zhang Z, Lin S, Lin N, et al. Disorders of the central nervous system: Insights from Notch and Nrf2 signaling. Biomed Pharmacother. 2023;166: 115383. 37643483 10.1016/j.biopha.2023.115383

[73] Kanamori M, Kawaguchi T, Nigro JM, Feuerstein BG, Berger MS, Miele L, Pieper RO. Contribution of Notch signaling activation to human glioblastoma multiforme. J Neurosurg. 2007;106(3):417–27. 17367064 10.3171/jns.2007.106.3.417

[74] Zhang X-P, Zheng G, Zou L, Liu H-L, Hou L-H, Zhou P, et al. Notch activation promotes cell proliferation and the formation of neural stem cell-like colonies in human glioma cells. Mol Cell Biochem. 2008;307:101–8. 17849174 10.1007/s11010-007-9589-0

[75] Phillips HS, Kharbanda S, Chen R, Forrest WF, Soriano RH, Wu TD, et al. Molecular subclasses of high-grade glioma predict prognosis, delineate a pattern of disease progression, and resemble stages in neurogenesis. Cancer Cell. 2006;9(3):157–73. 16530701 10.1016/j.ccr.2006.02.019

[76] Hulleman E, Quarto M, Vernell R, Masserdotti G, Colli E, Kros JM, et al. A role for the transcription factor HEY1 in glioblastoma. J Cell Mol Med. 2009;13(1):136–46. 18363832 10.1111/j.1582-4934.2008.00307.x PMC3823042

[77] El Hindy N, Keyvani K, Pagenstecher A, Dammann P, Sandalcioglu IE, Sure U, Zhu Y. Implications of Dll4-Notch signaling activation in primary glioblastoma multiforme. Neuro Oncol. 2013;15(10):1366–78. 23787764 10.1093/neuonc/not071 PMC3779034

[78] Margareto J, Larrarte E, Leis O, Carrasco A, Lafuente J, Idoate M. Gene expression profiling of human gliomas reveals differences between GBM and LGA related to energy metabolism and notch signaling pathways. J Mol Neurosci. 2007;32:53–63. 17873288 10.1007/s12031-007-0008-5

[79] Cheng W, Zhang C, Ren X, Jiang Y, Han S, Liu Y, et al. Bioinformatic analyses reveal a distinct Notch activation induced by STAT3 phosphorylation in the mesenchymal subtype of glioblastoma. J Neurosurg. 2017;126(1):249–59. 26967788 10.3171/2015.11.JNS15432

[80] Cooper LA, Gutman DA, Long Q, Johnson BA, Cholleti SR, Kurc T, et al. The proneural molecular signature is enriched in oligodendrogliomas and predicts improved survival among diffuse gliomas. PLoS ONE. 2010;5(9): e12548. 20838435 10.1371/journal.pone.0012548 PMC2933229

[81] Allen BK, Stathias V, Maloof ME, Vidovic D, Winterbottom EF, Capobianco AJ, et al. Epigenetic pathways and glioblastoma treatment: insights from signaling cascades. J Cell Biochem. 2015;116(3):351–63. 25290986 10.1002/jcb.24990

[82] Bazzoni R, Bentivegna A. Role of Notch signaling pathway in glioblastoma pathogenesis. Cancers. 2019;11(3):292. 30832246 10.3390/cancers11030292 PMC6468848

[83] Tsung AJ, Guda MR, Asuthkar S, Labak CM, Purvis IJ, Lu Y, et al. Methylation regulates HEY1 expression in glioblastoma. Oncotarget. 2017;8(27):44398. 28574840 10.18632/oncotarget.17897 PMC5546488

[84] Sun P, Xia S, Lal B, Eberhart CG, Quinones-Hinojosa A, Maciaczyk J, et al. DNER, an epigenetically modulated gene, regulates glioblastoma-derived neurosphere cell differentiation and tumor propagation. Stem cells. 2009;27(7):1473–86. 19544453 10.1002/stem.89 PMC2935595

[85] Velásquez C, Mansouri S, Mora C, Nassiri F, Suppiah S, Martino J, et al. Molecular and clinical insights into the invasive capacity of glioblastoma cells. Journal of Oncology. 2019;2019. 10.1155/2019/1740763 PMC6699388 31467533

[86] Liu Y, Liu X, Chen L-C, Du W-Z, Cui Y-Q, Piao X-Y, et al. Targeting glioma stem cells via the Hedgehog signaling pathway. Neuroimmunology and Neuroinflammation. 2014;1:51–9.

[87] Dlugosz AA, Talpaz M. Following the hedgehog to new cancer therapies. Mass Medical Soc; 2009. p. 1202–5. 10.1056/NEJMe0906092 19726764

[88] Yoo YA, Kang MH, Lee HJ, Kim B-h, Park JK, Kim HK, et al. Sonic hedgehog pathway promotes metastasis and lymphangiogenesis via activation of Akt, EMT, and MMP-9 pathway in gastric cancer. Cancer research. 2011;71(22):7061–70. 10.1158/0008-5472.CAN-11-1338 21975935

[89] Hayhurst M, McConnell SK. Mouse models of holoprosencephaly. Curr Opin Neurol. 2003;16(2):135–41. 12644739 10.1097/01.wco.0000063761.15877.40

[90] Bresler SC, Padwa BL, Granter SR. Nevoid basal cell carcinoma syndrome (Gorlin syndrome). Head Neck Pathol. 2016;10:119–24. 26971503 10.1007/s12105-016-0706-9 PMC4838974

[91] Caro I, Low JA. The role of the hedgehog signaling pathway in the development of basal cell carcinoma and opportunities for treatment. Clin Cancer Res. 2010;16(13):3335–9. 20439455 10.1158/1078-0432.CCR-09-2570

[92] Wu F, Zhang Y, Sun B, McMahon AP, Wang Y. Hedgehog signaling: from basic biology to cancer therapy. Cell Chem Biol. 2017;24(3):252–80. 28286127 10.1016/j.chembiol.2017.02.010 PMC7442121

[93] Giroux-Leprieur E, Costantini A, Ding VW, He B. Hedgehog signaling in lung cancer: From oncogenesis to cancer treatment resistance. Int J Mol Sci. 2018;19(9):2835. 30235830 10.3390/ijms19092835 PMC6165231

[94] Clement V, Sanchez P, de Tribolet N, Radovanovic I, i Altaba AR. HEDGEHOG-GLI1 signaling regulates human glioma growth, cancer stem cell self-renewal, and tumorigenicity. Current biology. 2007;17(2):165–72. 10.1016/j.cub.2006.11.033 PMC1855204 17196391

[95] Allen BL, Song JY, Izzi L, Althaus IW, Kang J-S, Charron F, et al. Overlapping roles and collective requirement for the coreceptors GAS1, CDO, and BOC in SHH pathway function. Dev Cell. 2011;20(6):775–87. 21664576 10.1016/j.devcel.2011.04.018 PMC3121104

[96] Robbins DJ, Fei DL, Riobo NA. The Hedgehog signal transduction network. Science signaling. 2012;5(246):re6-re. 10.1126/scisignal.2002906 PMC3705708 23074268

[97] Wang T, Liu X-h, Guan J, Ge S, Wu M-B, Lin J-p, Yang L-r. Advancement of multi-target drug discoveries and promising applications in the field of Alzheimer's disease. European Journal of Medicinal Chemistry. 2019;169:200–23. 10.1016/j.ejmech.2019.02.076 30884327

[98] Bar EE, Chaudhry A, Lin A, Fan X, Schreck K, Matsui W, et al. Cyclopamine-mediated hedgehog pathway inhibition depletes stem-like cancer cells in glioblastoma. Stem cells. 2007;25(10):2524–33. 17628016 10.1634/stemcells.2007-0166 PMC2610257

[99] Zbinden M, Duquet A, Lorente‐Trigos A, Ngwabyt SN, Borges I, Ruiz i Altaba A. NANOG regulates glioma stem cells and is essential in vivo acting in a cross‐functional network with GLI1 and p53. The EMBO journal. 2010;29(15):2659–74. 10.1038/emboj.2010.137 PMC2928692 20581802

[100] Ehtesham M, Sarangi A, Valadez J, Chanthaphaychith S, Becher M, Abel T, et al. Ligand-dependent activation of the Hedgehog pathway in glioma progenitor cells. Oncogene. 2007;26(39):5752–61. 17353902 10.1038/sj.onc.1210359

[101] Rimkus TK, Carpenter RL, Sirkisoon S, Zhu D, Pasche BC, Chan MD, et al. Truncated glioma-associated oncogene homolog 1 (tGLI1) mediates mesenchymal glioblastoma via transcriptional activation of CD44. Can Res. 2018;78(10):2589–600. 10.1158/0008-5472.CAN-17-2933 PMC5955849 29463580

[102] Sirkisoon SR, Carpenter RL, Rimkus T, Anderson A, Harrison A, Lange AM, et al. Interaction between STAT3 and GLI1/tGLI1 oncogenic transcription factors promotes the aggressiveness of triple-negative breast cancers and HER2-enriched breast cancer. Oncogene. 2018;37(19):2502–14. 29449694 10.1038/s41388-018-0132-4 PMC5948110

[103] Wang K, Pan L, Che X, Cui D, Li C. Sonic Hedgehog/GLI1 signaling pathway inhibition restricts cell migration and invasion in human gliomas. Neurol Res. 2010;32(9):975–80. 20444323 10.1179/016164110X12681290831360

[104] Uchida H, Arita K, Yunoue S, Yonezawa H, Shinsato Y, Kawano H, et al. Role of sonic hedgehog signaling in migration of cell lines established from CD133-positive malignant glioma cells. J Neurooncol. 2011;104:697–704. 21380601 10.1007/s11060-011-0552-2

[105] Wang YH, Sui XM, Sui YN, Zhu QW, Yan K, Wang LS, et al. BRD4 induces cell migration and invasion in HCC cells through MMP-2 and MMP-9 activation mediated by the Sonic Hedgehog signaling pathway. Oncol Lett. 2015;10(4):2227–32. 26622824 10.3892/ol.2015.3570 PMC4579919

[106] Tang Y, Gholamin S, Schubert S, Willardson MI, Lee A, Bandopadhayay P, et al. Epigenetic targeting of Hedgehog pathway transcriptional output through BET bromodomain inhibition. Nat Med. 2014;20(7):732–40. 24973920 10.1038/nm.3613 PMC4108909

[107] Malatesta M, Steinhauer C, Mohammad F, Pandey DP, Squatrito M, Helin K. Histone acetyltransferase PCAF is required for Hedgehog–Gli-dependent transcription and cancer cell proliferation. Can Res. 2013;73(20):6323–33. 10.1158/0008-5472.CAN-12-4660 23943798

[108] Maresca L, Crivaro E, Migliorini F, Anichini G, Giammona A, Pepe S, et al. Targeting GLI1 and GLI2 with small molecule inhibitors to suppress GLI-dependent transcription and tumor growth. Pharmacol Res. 2023;195: 106858. 37473878 10.1016/j.phrs.2023.106858

[109] McCord M, Mukouyama Y-S, Gilbert MR, Jackson S. Targeting WNT signaling for multifaceted glioblastoma therapy. Front Cell Neurosci. 2017;11:318. 29081735 10.3389/fncel.2017.00318 PMC5645527

[110] Logan CY, Nusse R. The Wnt signaling pathway in development and disease. Annu Rev Cell Dev Biol. 2004;20:781–810. 15473860 10.1146/annurev.cellbio.20.010403.113126

[111] D’Alimonte I, Nargi E, Lannutti A, Marchisio M, Pierdomenico L, Costanzo G, et al. Adenosine A1 receptor stimulation enhances osteogenic differentiation of human dental pulp-derived mesenchymal stem cells via WNT signaling. Stem cell research. 2013;11(1):611–24. 23651584 10.1016/j.scr.2013.04.002

[112] McMahon AP, Bradley A. The Wnt-1 (int-1) proto-oncogene is required for development of a large region of the mouse brain. Cell. 1990;62(6):1073–85. 2205396 10.1016/0092-8674(90)90385-r

[113] Zhou C-J, Pinson KI, Pleasure SJ. Severe defects in dorsal thalamic development in low-density lipoprotein receptor-related protein-6 mutants. J Neurosci. 2004;24(35):7632–9. 15342729 10.1523/JNEUROSCI.2123-04.2004 PMC6729615

[114] Klaus A, Birchmeier W. Wnt signalling and its impact on development and cancer. Nat Rev Cancer. 2008;8(5):387–98. 18432252 10.1038/nrc2389

[115] Zhan T, Rindtorff N, Boutros M. Wnt signaling in cancer. Oncogene. 2017;36(11):1461–73. 27617575 10.1038/onc.2016.304 PMC5357762

[116] Zarkou V, Galaras A, Giakountis A, Hatzis P. Crosstalk mechanisms between the WNT signaling pathway and long non-coding RNAs. Non-coding RNA research. 2018;3(2):42–53. 30159439 10.1016/j.ncrna.2018.04.001 PMC6096407

[117] Widelitz R. Wnt signaling through canonical and non-canonical pathways: recent progress. Growth Factors. 2005;23(2):111–6. 16019432 10.1080/08977190500125746

[118] Niehrs C. The complex world of WNT receptor signalling. Nat Rev Mol Cell Biol. 2012;13(12):767–79. 23151663 10.1038/nrm3470

[119] Blache P, Van De Wetering M, Duluc I, Domon C, Berta P, Freund J-N, et al. SOX9 is an intestine crypt transcription factor, is regulated by the Wnt pathway, and represses the CDX2 and MUC2 genes. J Cell Biol. 2004;166(1):37–47. 15240568 10.1083/jcb.200311021 PMC2172132

[120] Zheng H, Ying H, Wiedemeyer R, Yan H, Quayle SN, Ivanova EV, et al. PLAGL2 regulates Wnt signaling to impede differentiation in neural stem cells and gliomas. Cancer Cell. 2010;17(5):497–509. 20478531 10.1016/j.ccr.2010.03.020 PMC2900858

[121] Jin X, Jeon H-Y, Joo KM, Kim J-K, Jin J, Kim SH, et al. Frizzled 4 regulates stemness and invasiveness of migrating glioma cells established by serial intracranial transplantation. Can Res. 2011;71(8):3066–75. 10.1158/0008-5472.CAN-10-1495 21363911

[122] Holland JD, Klaus A, Garratt AN, Birchmeier W. Wnt signaling in stem and cancer stem cells. Curr Opin Cell Biol. 2013;25(2):254–64. 23347562 10.1016/j.ceb.2013.01.004

[123] Noda T, Nagano H, Takemasa I, Yoshioka S, Murakami M, Wada H, et al. Activation of Wnt/β-catenin signalling pathway induces chemoresistance to interferon-α/5-fluorouracil combination therapy for hepatocellular carcinoma. Br J Cancer. 2009;100(10):1647–58. 19401692 10.1038/sj.bjc.6605064 PMC2696759

[124] Sandberg CJ, Altschuler G, Jeong J, Strømme KK, Stangeland B, Murrell W, et al. Comparison of glioma stem cells to neural stem cells from the adult human brain identifies dysregulated Wnt-signaling and a fingerprint associated with clinical outcome. Exp Cell Res. 2013;319(14):2230–43. 23791939 10.1016/j.yexcr.2013.06.004

[125] Denysenko T, Annovazzi L, Cassoni P, Melcarne A, Mellai M, Schiffer D. WNT/β-catenin signaling pathway and downstream modulators in low-and high-grade glioma. Cancer Genomics Proteomics. 2016;13(1):31–45. 26708597

[126] Chen L, Huang K, Han L, Shi Z, Zhang K, Pu P, et al. β-catenin/Tcf-4 complex transcriptionally regulates AKT1 in glioma. Int J Oncol. 2011;39(4):883–90. 21720709 10.3892/ijo.2011.1104

[127] Zhang K, Zhang J, Han L, Pu P, Kang C. Wnt/beta-catenin signaling in glioma. J Neuroimmune Pharmacol. 2012;7:740–9. 22454041 10.1007/s11481-012-9359-y

[128] Gong A, Huang S. FoxM1 and Wnt/β-catenin signaling in glioma stem cells. Can Res. 2012;72(22):5658–62. 10.1158/0008-5472.CAN-12-0953 PMC3500394 23139209

[129] Zhang M, Atkinson RL, Rosen JM. Selective targeting of radiation-resistant tumor-initiating cells. Proc Natl Acad Sci. 2010;107(8):3522–7. 20133717 10.1073/pnas.0910179107 PMC2840501

[130] Arnés M, Casas TS. Aberrant Wnt signaling: a special focus in CNS diseases. J Neurogenet. 2017;31(4):216–22. 28635355 10.1080/01677063.2017.1338696

[131] Götze S, Wolter M, Reifenberger G, Müller O, Sievers S. Frequent promoter hypermethylation of Wnt pathway inhibitor genes in malignant astrocytic gliomas. Int J Cancer. 2010;126(11):2584–93. 19847810 10.1002/ijc.24981

[132] Vassallo I, Zinn P, Lai M, Rajakannu P, Hamou M, Hegi M. WIF1 re-expression in glioblastoma inhibits migration through attenuation of non-canonical WNT signaling by downregulating the lncRNA MALAT1. Oncogene. 2016;35(1):12–21. 25772239 10.1038/onc.2015.61

[133] Lövkvist C, Dodd IB, Sneppen K, Haerter JO. DNA methylation in human epigenomes depends on local topology of CpG sites. Nucleic Acids Res. 2016;44(11):5123–32. 26932361 10.1093/nar/gkw124 PMC4914085

[134] Bird A. DNA methylation patterns and epigenetic memory. Genes Dev. 2002;16(1):6–21. 11782440 10.1101/gad.947102

[135] Liao P, Ostrom QT, Stetson L, Barnholtz-Sloan JS. Models of epigenetic age capture patterns of DNA methylation in glioma associated with molecular subtype, survival, and recurrence. Neuro Oncol. 2018;20(7):942–53. 29432558 10.1093/neuonc/noy003 PMC6007761

[136] Choudhury SR, Cui Y, Milton JR, Li J, Irudayaraj J. Selective increase in subtelomeric DNA methylation: an epigenetic biomarker for malignant glioma. Clin Epigenetics. 2015;7:1–11. 26451167 10.1186/s13148-015-0140-y PMC4597615

[137] Kraus TF, Globisch D, Wagner M, Eigenbrod S, Widmann D, Münzel M, et al. Low values of 5-hydroxymethylcytosine (5hmC), the “sixth base”, are associated with anaplasia in human brain tumors. Int J Cancer. 2012;131(7):1577–90. 22234893 10.1002/ijc.27429

[138] Mack SC, Witt H, Piro R, Gu L, Zuyderduyn S, Stütz A, et al. Epigenomic alterations define lethal CIMP-positive ependymomas of infancy. Nature. 2014;506(7489):445–50. 24553142 10.1038/nature13108 PMC4174313

[139] Malta TM, de Souza CF, Sabedot TS, Silva TC, Mosella MS, Kalkanis SN, et al. Glioma CpG island methylator phenotype (G-CIMP): biological and clinical implications. Neuro Oncol. 2018;20(5):608–20. 29036500 10.1093/neuonc/nox183 PMC5892155

[140] Lee S, Kim M, Kwon H, Park I, Park M, Lee C, et al. Growth inhibitory effect on glioma cells of adenovirus-mediated p16/INK4a gene transfer in vitro and in vivo. Int J Mol Med. 2000;6(5):559–622. 11029524

[141] Kolodziej MA, Weischer C, Reinges MH, Uhl E, Weigand MA, Schwarm FP, et al. NDRG2 and NDRG4 expression is altered in glioblastoma and influences survival in patients with MGMT-methylated tumors. Anticancer Res. 2016;36(3):887–97. 26976975

[142] Esteller M, Garcia-Foncillas J, Andion E, Goodman SN, Hidalgo OF, Vanaclocha V, et al. Inactivation of the DNA-repair gene MGMT and the clinical response of gliomas to alkylating agents. N Engl J Med. 2000;343(19):1350–4. 11070098 10.1056/NEJM200011093431901

[143] Yu Z, Chen Y, Wang S, Li P, Zhou G, Yuan Y. Inhibition of NF-κB results in anti-glioma activity and reduces temozolomide-induced chemoresistance by down-regulating MGMT gene expression. Cancer Lett. 2018;428:77–89. 29705182 10.1016/j.canlet.2018.04.033

[144] Jiang Z, Li X, Hu J, Zhou W, Jiang Y, Li G, Lu D. Promoter hypermethylation-mediated down-regulation of LATS1 and LATS2 in human astrocytoma. Neurosci Res. 2006;56(4):450–8. 17049657 10.1016/j.neures.2006.09.006

[145] Watanabe T, Huang H, Nakamura M, Wischhusen J, Weller M, Kleihues P, Ohgaki H. Methylation of the p73 gene in gliomas. Acta Neuropathol. 2002;104:357–62. 12200621 10.1007/s00401-002-0549-1

[146] Schwartzentruber J, Korshunov A, Liu X-Y, Jones DT, Pfaff E, Jacob K, et al. Driver mutations in histone H3. 3 and chromatin remodelling genes in paediatric glioblastoma. Nature. 2012;482(7384):226–31. 10.1038/nature10833 22286061

[147] Marks PA, Breslow R. Dimethyl sulfoxide to vorinostat: development of this histone deacetylase inhibitor as an anticancer drug. Nat Biotechnol. 2007;25(1):84–90. 17211407 10.1038/nbt1272

[148] Sekar S, Liang WS. Circular RNA expression and function in the brain. Non-coding RNA research. 2019;4(1):23–9. 30891534 10.1016/j.ncrna.2019.01.001 PMC6404376

[149] Rasool M, Malik A, Zahid S, Basit Ashraf M, Qazi M, Asif M, et al. Non-coding RNAs in cancer diagnosis and therapy. Non-Coding RNA Res. 2016;1:69–76. 10.1016/j.ncrna.2016.11.001 PMC6096421 30159413

[150] Alfardus H, McIntyre A, Smith S. MicroRNA regulation of glycolytic metabolism in glioblastoma. BioMed research international. 2017;2017. 10.1155/2017/9157370 PMC5539934 28804724

[151] Dai D-W, Lu Q, Wang L-X, Zhao W-Y, Cao Y-Q, Li Y-N, et al. Decreased miR-106a inhibits glioma cell glucose uptake and proliferation by targeting SLC2A3 in GBM. BMC Cancer. 2013;13(1):1–8. 24124917 10.1186/1471-2407-13-478 PMC3853007

[152] Zhao S, Liu H, Liu Y, Wu J, Wang C, Hou X, et al. miR-143 inhibits glycolysis and depletes stemness of glioblastoma stem-like cells. Cancer Lett. 2013;333(2):253–60. 23376635 10.1016/j.canlet.2013.01.039

[153] Kefas B, Comeau L, Erdle N, Montgomery E, Amos S, Purow B. Pyruvate kinase M2 is a target of the tumor-suppressive microRNA-326 and regulates the survival of glioma cells. Neuro Oncol. 2010;12(11):1102–12. 20667897 10.1093/neuonc/noq080 PMC3098027

[154] Luan W, Wang Y, Chen X, Shi Y, Wang J, Zhang J, et al. PKM2 promotes glucose metabolism and cell growth in gliomas through a mechanism involving a let-7a/c-Myc/hnRNPA1 feedback loop. Oncotarget. 2015;6(15):13006. 25948776 10.18632/oncotarget.3514 PMC4536995

[155] Liu Z, Wang J, Li Y, Fan J, Chen L, Xu R. MicroRNA-153 regulates glutamine metabolism in glioblastoma through targeting glutaminase. Tumor Biology. 2017;39(2):1010428317691429. 28218035 10.1177/1010428317691429

[156] Xu G, Li JY. ATP5A1 and ATP5B are highly expressed in glioblastoma tumor cells and endothelial cells of microvascular proliferation. J Neurooncol. 2016;126:405–13. 26526033 10.1007/s11060-015-1984-x

[157] Yang W, Xia Y, Cao Y, Zheng Y, Bu W, Zhang L, et al. Erratum: EGFR-induced and PKCε monoubiquitylation-dependent NF-κB activation upregulates PKM2 expression and promotes tumorigenesis (molecular cell (2012) 48 (5)(771–784)(S1097276512008283)(10.1016/j. molcel. 2012.09. 028)). Molecular cell. 2018;69(2):347.

[158] Rao SAM, Arimappamagan A, Pandey P, Santosh V, Hegde AS, Chandramouli BA, Somasundaram K. miR-219-5p inhibits receptor tyrosine kinase pathway by targeting EGFR in glioblastoma. PLoS ONE. 2013;8(5): e63164. 23690991 10.1371/journal.pone.0063164 PMC3656853

[159] Papagiannakopoulos T, Friedmann-Morvinski D, Neveu P, Dugas J, Gill R, Huillard E, et al. Pro-neural miR-128 is a glioma tumor suppressor that targets mitogenic kinases. Oncogene. 2012;31(15):1884–95. 21874051 10.1038/onc.2011.380 PMC4160048

[160] Chan JA, Krichevsky AM, Kosik KS. MicroRNA-21 is an antiapoptotic factor in human glioblastoma cells. Can Res. 2005;65(14):6029–33. 10.1158/0008-5472.CAN-05-0137 16024602

[161] Gaglio D, Metallo CM, Gameiro PA, Hiller K, Danna LS, Balestrieri C, et al. Oncogenic K-Ras decouples glucose and glutamine metabolism to support cancer cell growth. Mol Syst Biol. 2011;7(1):523. 21847114 10.1038/msb.2011.56 PMC3202795

[162] Zhang Y, Kim J, Mueller A, Dey B, Yang Y, Lee D, et al. Correction to: multiple receptor tyrosine kinases converge on microRNA-134 to control KRAS, STAT5B, and glioblastoma. Cell Death Differ. 2019;26(1):197. 29899381 10.1038/s41418-018-0145-0 PMC6294759

[163] Tan X, Wang S, Yang B, Zhu L, Yin B, Chao T, et al. The CREB-miR-9 negative feedback minicircuitry coordinates the migration and proliferation of glioma cells. PLoS ONE. 2012;7(11): e49570. 23185366 10.1371/journal.pone.0049570 PMC3502497

[164] Wu Z, Wang L, Li G, Liu H, Fan F, Li Z, et al. Increased expression of microRNA-9 predicts an unfavorable prognosis in human glioma. Mol Cell Biochem. 2013;384:263–8. 24122417 10.1007/s11010-013-1805-5

[165] Masui K, Tanaka K, Akhavan D, Babic I, Gini B, Matsutani T, et al. mTOR complex 2 controls glycolytic metabolism in glioblastoma through FoxO acetylation and upregulation of c-Myc. Cell Metab. 2013;18(5):726–39. 24140020 10.1016/j.cmet.2013.09.013 PMC3840163

[166] Zhou C, Zhang Y, Dai J, Zhou M, Liu M, Wang Y, et al. Pygo2 functions as a prognostic factor for glioma due to its up-regulation of H3K4me3 and promotion of MLL1/MLL2 complex recruitment. Sci Rep. 2016;6(1):22066. 26902498 10.1038/srep22066 PMC4763266

[167] Stanton BZ, Hodges C, Crabtree GR, Zhao K. A General non-radioactive ATPase assay for chromatin remodeling complexes. Current protocols in chemical biology. 2017;9(1):1–10. 28253434 10.1002/cpch.16 PMC5334659

[168] Banasavadi-Siddegowda YK, Welker AM, An M, Yang X, Zhou W, Shi G, et al. PRMT5 as a druggable target for glioblastoma therapy. Neuro Oncol. 2018;20(6):753–63. 29106602 10.1093/neuonc/nox206 PMC5961180

[169] Choi YJ, Yoo NJ, Lee SH. Mutation of HELLS, a chromatin remodeling gene, gastric and colorectal cancers. Pathology & Oncology Research. 2015;21:851–2. 25351940 10.1007/s12253-014-9862-y

[170] Liau BB, Sievers C, Donohue LK, Gillespie SM, Flavahan WA, Miller TE, et al. Adaptive chromatin remodeling drives glioblastoma stem cell plasticity and drug tolerance. Cell stem cell. 2017;20(2):233–46. e7. 10.1016/j.stem.2016.11.003 PMC5291795 27989769

[171] Xiao D, Huang J, Pan Y, Li H, Fu C, Mao C, et al. Chromatin remodeling factor LSH is upregulated by the LRP6-GSK3β-E2F1 axis linking reversely with survival in gliomas. Theranostics. 2017;7(1):132. 28042322 10.7150/thno.17032 PMC5196891

[172] Zhao D. Single nucleotide alterations in microRNAs and human cancer-a not fully explored field. Non-coding RNA research. 2020;5(1):27–31. 32128468 10.1016/j.ncrna.2020.02.003 PMC7044681

[173] ElKhouly AM, Youness R, Gad M. MicroRNA-486-5p and microRNA-486-3p: multifaceted pleiotropic mediators in oncological and non-oncological conditions. Non-coding RNA research. 2020;5(1):11–21. 31993547 10.1016/j.ncrna.2020.01.001 PMC6971376

[174] Kwok GT, Zhao JT, Weiss J, Mugridge N, Brahmbhatt H, MacDiarmid JA, et al. Translational applications of microRNAs in cancer, and therapeutic implications. Non-coding RNA research. 2017;2(3–4):143–50. 30159433 10.1016/j.ncrna.2017.12.002 PMC6084838

[175] Rajgor D. Macro roles for microRNAs in neurodegenerative diseases. Non-Coding RNA Research. 2018;3(3):154–9. 30175288 10.1016/j.ncrna.2018.07.001 PMC6114258

[176] Wang G, Fu XL, Wang JJ, Guan R, Sun Y, Tony To Ss. Inhibition of glycolytic metabolism in glioblastoma cells by Pt3glc combinated with PI3K inhibitor via SIRT3‐mediated mitochondrial and PI3K/Akt–MAPK pathway. Journal of Cellular Physiology. 2019;234(5):5888–903. 10.1002/jcp.26474 29336479

[177] Cai J, Zhao J, Zhang N, Xu X, Li R, Yi Y, et al. MicroRNA-542-3p suppresses tumor cell invasion via targeting AKT pathway in human astrocytoma. J Biol Chem. 2015;290(41):24678–88. 26286747 10.1074/jbc.M115.649004 PMC4598981

[178] Liu Z, Jiang Z, Huang J, Huang S, Li Y, Yu S, et al. miR-7 inhibits glioblastoma growth by simultaneously interfering with the PI3K/ATK and Raf/MEK/ERK pathways. Int J Oncol. 2014;44(5):1571–80. 24603851 10.3892/ijo.2014.2322

[179] Miyake N, Chikumi H, Takata M, Nakamoto M, Igishi T, Shimizu E. Rapamycin induces p53-independent apoptosis through the mitochondrial pathway in non-small cell lung cancer cells. Oncol Rep. 2012;28(3):848–54. 22710790 10.3892/or.2012.1855

[180] Guo P, Nie Q, Lan J, Ge J, Qiu Y, Mao Q. C-Myc negatively controls the tumor suppressor PTEN by upregulating miR-26a in glioblastoma multiforme cells. Biochem Biophys Res Commun. 2013;441(1):186–90. 24140063 10.1016/j.bbrc.2013.10.034

[181] Xia X, Li Y, Wang W, Tang F, Tan J, Sun L, et al. MicroRNA-1908 functions as a glioblastoma oncogene by suppressing PTEN tumor suppressor pathway. Mol Cancer. 2015;14(1):1–14. 26265437 10.1186/s12943-015-0423-0 PMC4534015

[182] Li X-t, Wang H-z, Wu Z-w, Yang T-q, Zhao Z-h, Chen G-l, et al. miR-494-3p regulates cellular proliferation, invasion, migration, and apoptosis by PTEN/AKT signaling in human glioblastoma cells. Cell Mol Neurobiol. 2015;35:679–87. 25662849 10.1007/s10571-015-0163-0 PMC4477718

[183] Koppenol WH, Bounds PL. The Warburg effect and metabolic efficiency: re-crunching the numbers. Science. 2009;324(5930):1029–33. 19460998 10.1126/science.1160809 PMC2849637

[184] Shen L, Sun C, Li Y, Li X, Sun T, Liu C, et al. MicroRNA-199a-3p suppresses glioma cell proliferation by regulating the AKT/mTOR signaling pathway. Tumor Biology. 2015;36:6929–38. 25854175 10.1007/s13277-015-3409-z PMC4644202

[185] Vargas JE, Filippi-Chiela EC, Suhre T, Kipper FC, Bonatto D, Lenz G. Inhibition of HDAC increases the senescence induced by natural polyphenols in glioma cells. Biochem Cell Biol. 2014;92(4):297–304. 25070040 10.1139/bcb-2014-0022

[186] Ghildiyal R, Sen E. Concerted action of histone methyltransferases G9a and PRMT-1 regulates PGC-1α-RIG-I axis in IFNγ treated glioma cells. Cytokine. 2017;89:185–93. 26725954 10.1016/j.cyto.2015.12.008

[187] Guo AS, Huang YQ, Ma XD, Lin RS. Mechanism of G9a inhibitor BIX-01294 acting on U251 glioma cells. Mol Med Rep. 2016;14(5):4613–21. 27748874 10.3892/mmr.2016.5815 PMC5102021

[188] Chen X, Ma H, Wang Z, Zhang S, Yang H, Fang Z. EZH2 palmitoylation mediated by ZDHHC5 in p53-mutant glioma drives malignant development and progression. Can Res. 2017;77(18):4998–5010. 10.1158/0008-5472.CAN-17-1139 28775165

[189] Kurmasheva RT, Sammons M, Favours E, Wu J, Kurmashev D, Cosmopoulos K, et al. Initial testing (stage 1) of tazemetostat (EPZ-6438), a novel EZH2 inhibitor, by the pediatric preclinical testing program. Pediatr Blood Cancer. 2017;64(3): e26218. 10.1002/pbc.26218 PMC5584632 27555605

[190] Zhang Y, Dong W, Zhu J, Wang L, Wu X, Shan H. Combination of EZH2 inhibitor and BET inhibitor for treatment of diffuse intrinsic pontine glioma. Cell Biosci. 2017;7:1–10. 29118968 10.1186/s13578-017-0184-0 PMC5663148

[191] Cheng C, Ru P, Geng F, Liu J, Yoo JY, Wu X, et al. Glucose-mediated N-glycosylation of SCAP is essential for SREBP-1 activation and tumor growth. Cancer Cell. 2015;28(5):569–81. 26555173 10.1016/j.ccell.2015.09.021 PMC4643405

[192] Ru P, Guo D. microRNA-29 mediates a novel negative feedback loop to regulate SCAP/SREBP-1 and lipid metabolism. RNA & disease (Houston, Tex). 2017;4(1). 10.14800/rd.1525 PMC5485916 28664184

[193] Berindan‐Neagoe I, Monroig PdC, Pasculli B, Calin GA. MicroRNAome genome: a treasure for cancer diagnosis and therapy. CA: a cancer journal for clinicians. 2014;64(5):311–36. 10.3322/caac.21244 PMC4461198 25104502

[194] Zang L, Kondengaden SM, Che F, Wang L, Heng X. Potential epigenetic-based therapeutic targets for glioma. Front Mol Neurosci. 2018;11:408. 30498431 10.3389/fnmol.2018.00408 PMC6249994

[195] Zhou X, Ren Y, Moore L, Mei M, You Y, Xu P, et al. Downregulation of miR-21 inhibits EGFR pathway and suppresses the growth of human glioblastoma cells independent of PTEN status. Lab Invest. 2010;90(2):144–55. 20048743 10.1038/labinvest.2009.126

[196] Zhang C, Zhang J, Hao J, Shi Z, Wang Y, Han L, et al. High level of miR-221/222 confers increased cell invasion and poor prognosis in glioma. J Transl Med. 2012;10(1):1–11. 22681957 10.1186/1479-5876-10-119 PMC3403924

[197] Zhang C, Kang C, You Y, Pu P, Yang W, Zhao P, et al. Co-suppression of miR-221/222 cluster suppresses human glioma cell growth by targeting p27kip1 in vitro and in vivo. Int J Oncol. 2009;34(6):1653–60. 19424584 10.3892/ijo_00000296

[198] Shankaraiah RC, Veronese A, Sabbioni S, Negrini M. Non-coding RNAs in the reprogramming of glucose metabolism in cancer. Cancer Lett. 2018;419:167–74. 29366802 10.1016/j.canlet.2018.01.048

[199] Balas MM, Johnson AM. Exploring the mechanisms behind long noncoding RNAs and cancer. Non-coding RNA research. 2018;3(3):108–17. 30175284 10.1016/j.ncrna.2018.03.001 PMC6114262

[200] Grixti JM, Ayers D. Long noncoding RNAs and their link to cancer. Non-coding RNA research. 2020;5(2):77–82. 32490292 10.1016/j.ncrna.2020.04.003 PMC7256057

[201] Ayers D, Scerri C. Non-coding RNA influences in dementia. Non-coding RNA research. 2018;3(4):188–94. 30533568 10.1016/j.ncrna.2018.09.002 PMC6260472

[202] An H, Williams NG, Shelkovnikova TA. NEAT1 and paraspeckles in neurodegenerative diseases: a missing lnc found? Non-coding RNA research. 2018;3(4):243–52. 30533572 10.1016/j.ncrna.2018.11.003 PMC6257911

[203] Chen X, Gao Y, Li D, Cao Y, Hao B. LncRNA-TP53TG1 participated in the stress response under glucose deprivation in glioma. J Cell Biochem. 2017;118(12):4897–904. 28569381 10.1002/jcb.26175

[204] Yang Y, Gao X, Zhang M, Yan S, Sun C, Xiao F, et al. Novel role of FBXW7 circular RNA in repressing glioma tumorigenesis. JNCI: Journal of the National Cancer Institute. 2018;110(3):304–15. 10.1093/jnci/djx166 PMC6019044 28903484

[205] Dong Z, Cui H, editors. Epigenetic modulation of metabolism in glioblastoma. Seminars in cancer biology; 2019: Elsevier. 10.1016/j.semcancer.2018.09.002 30205139

[206] Norozi F, Ahmadzadeh A, Shahrabi S, Vosoughi T, Saki N. Mesenchymal stem cells as a double-edged sword in suppression or progression of solid tumor cells. Tumor Biology. 2016;37:11679–89. 27440203 10.1007/s13277-016-5187-7

[207] Cloughesy TF, Cavenee WK, Mischel PS. Glioblastoma: from molecular pathology to targeted treatment. Annu Rev Pathol. 2014;9:1–25. 23937436 10.1146/annurev-pathol-011110-130324

[208] Wang R-j, Li J-w, Bao B-h, Wu H-c, Du Z-h, Su J-l, et al. MicroRNA-873 (miRNA-873) inhibits glioblastoma tumorigenesis and metastasis by suppressing the expression of IGF2BP1. J Biol Chem. 2015;290(14):8938–48. 25670861 10.1074/jbc.M114.624700 PMC4423684

[209] Mo X, Cao Q, Liang H, Liu J, Li H, Liu F. MicroRNA-610 suppresses the proliferation of human glioblastoma cells by repressing CCND2 and AKT3. Mol Med Rep. 2016;13(3):1961–6. 26782072 10.3892/mmr.2016.4760 PMC4768983

[210] Clarke J, Penas C, Pastori C, Komotar RJ, Bregy A, Shah AH, et al. Epigenetic pathways and glioblastoma treatment. Epigenetics. 2013;8(8):785–95. 23807265 10.4161/epi.25440 PMC3883781

[211] Rasras S, Zibara K, Vosughi T, Zayeri Z. Genetics and epigenetics of glioblastoma: therapeutic challenges. Clinical Cancer Investigation Journal. 2018;7(2).

[212] Nebbioso A, Carafa V, Benedetti R, Altucci L. Trials with ‘epigenetic’drugs: an update. Mol Oncol. 2012;6(6):657–82. 23103179 10.1016/j.molonc.2012.09.004 PMC5528349

[213] Yuan J, Llamas Luceño N, Sander B, Golas MM. Synergistic anti-cancer effects of epigenetic drugs on medulloblastoma cells. Cell Oncol. 2017;40:263–79. 10.1007/s13402-017-0319-7 PMC13001549 28429280

[214] Kelly AD, Issa J-PJ. The promise of epigenetic therapy: reprogramming the cancer epigenome. Current opinion in genetics & development. 2017;42:68–77. 10.1016/j.gde.2017.03.015 28412585

[215] Hazane-Puch F, Arnaud J, Trocmé C, Faure P, Laporte F, Champelovier P. Sodium selenite decreased HDAC activity, cell proliferation and induced apoptosis in three human glioblastoma cells. Anti-Cancer Agents in Medicinal Chemistry (Formerly Current Medicinal Chemistry-Anti-Cancer Agents). 2016;16(4):490–500. 10.2174/1871520615666150819095426 26286659

[216] Pei Y, Liu K-W, Wang J, Garancher A, Tao R, Esparza LA, et al. HDAC and PI3K antagonists cooperate to inhibit growth of MYC-driven medulloblastoma. Cancer Cell. 2016;29(3):311–23. 26977882 10.1016/j.ccell.2016.02.011 PMC4794752

[217] Pathania R, Ramachandran S, Mariappan G, Thakur P, Shi H, Choi J-H, et al. Combined inhibition of DNMT and HDAC blocks the tumorigenicity of cancer stem-like cells and attenuates mammary tumor growth. Can Res. 2016;76(11):3224–35. 10.1158/0008-5472.CAN-15-2249 PMC4891240 27197203

[218] Krauze AV, Myrehaug SD, Chang MG, Holdford DJ, Smith S, Shih J, et al. A phase 2 study of concurrent radiation therapy, temozolomide, and the histone deacetylase inhibitor valproic acid for patients with glioblastoma. International Journal of Radiation Oncology* Biology* Physics. 2015;92(5):986–92. 10.1016/j.ijrobp.2015.04.038 PMC4510472 26194676

[219] Kim WJ, Newman WC, Amankulor NM. Phase I/II trial of combination of temozolomide chemotherapy and immunotherapy with fusions of dendritic and glioma cells in patients with glioblastoma. Neurosurgery. 2017;81(1):N11. 28873996 10.1093/neuros/nyx263

[220] Hummel TR, Wagner L, Ahern C, Fouladi M, Reid JM, McGovern RM, et al. A pediatric phase 1 trial of vorinostat and temozolomide in relapsed or refractory primary brain or spinal cord tumors: a Children’s Oncology Group phase 1 consortium study. Pediatr Blood Cancer. 2013;60(9):1452–7. 23554030 10.1002/pbc.24541 PMC4139006

[221] Galanis E, Jaeckle KA, Maurer MJ, Reid JM, Ames MM, Hardwick JS, et al. Phase II trial of vorinostat in recurrent glioblastoma multiforme: a north central cancer treatment group study. J Clin Oncol. 2009;27(12):2052. 19307505 10.1200/JCO.2008.19.0694 PMC2669764

[222] Chen D, Yang J. Development of novel antigen receptors for CAR T-cell therapy directed toward solid malignancies. Transl Res. 2017;187:11–21. 28641074 10.1016/j.trsl.2017.05.006

[223] Sadelain M, Rivière I, Riddell S. Therapeutic T cell engineering. Nature. 2017;545(7655):423–31. 28541315 10.1038/nature22395 PMC5632949

[224] Bagley SJ, O’Rourke DM. Clinical investigation of CAR T cells for solid tumors: Lessons learned and future directions. Pharmacol Ther. 2020;205: 107419. 31629009 10.1016/j.pharmthera.2019.107419

[225] Riddell SR, Sommermeyer D, Berger C, Liu LS, Balakrishnan A, Salter A, et al. Adoptive therapy with chimeric antigen receptor modified T cells of defined subset composition. Cancer Journal (Sudbury, Mass). 2014;20(2):141. 24667960 10.1097/PPO.0000000000000036 PMC4149222

[226] Kershaw MH, Westwood JA, Slaney CY, Darcy PK. Clinical application of genetically modified T cells in cancer therapy. Clinical & translational immunology. 2014;3(5): e16. 25505964 10.1038/cti.2014.7 PMC4232070

[227] Maude SL, Laetsch TW, Buechner J, Rives S, Boyer M, Bittencourt H, et al. Tisagenlecleucel in children and young adults with B-cell lymphoblastic leukemia. N Engl J Med. 2018;378(5):439–48. 29385370 10.1056/NEJMoa1709866 PMC5996391

[228] Locke FL, Ghobadi A, Jacobson CA, Miklos DB, Lekakis LJ, Oluwole OO, et al. Long-term safety and activity of axicabtagene ciloleucel in refractory large B-cell lymphoma (ZUMA-1): a single-arm, multicentre, phase 1–2 trial. Lancet Oncol. 2019;20(1):31–42. 30518502 10.1016/S1470-2045(18)30864-7 PMC6733402

[229] Samadani AA, Keymoradzdeh A, Shams S, Soleymanpour A, Rashidy-Pour A, Hashemian H, et al. CAR T-cells profiling in carcinogenesis and tumorigenesis: an overview of CAR T-cells cancer therapy. Int Immunopharmacol. 2021;90: 107201. 33249047 10.1016/j.intimp.2020.107201

[230] Ko AH, Jordan AC, Tooker E, Lacey SF, Chang RB, Li Y, et al. Dual targeting of mesothelin and CD19 with chimeric antigen receptor-modified T cells in patients with metastatic pancreatic cancer. Mol Ther. 2020;28(11):2367–78. 32730744 10.1016/j.ymthe.2020.07.017 PMC7647666

[231] Zhang C, Wang Z, Yang Z, Wang M, Li S, Li Y, et al. Phase I escalating-dose trial of CAR-T therapy targeting CEA+ metastatic colorectal cancers. Mol Ther. 2017;25(5):1248–58. 28366766 10.1016/j.ymthe.2017.03.010 PMC5417843

[232] Lamers CH, Klaver Y, Gratama JW, Sleijfer S, Debets R. Treatment of metastatic renal cell carcinoma (mRCC) with CAIX CAR-engineered T-cells–a completed study overview. Biochem Soc Trans. 2016;44(3):951–9. 27284065 10.1042/BST20160037

[233] Kandalaft LE, Powell DJ, Coukos G. A phase I clinical trial of adoptive transfer of folate receptor-alpha redirected autologous T cells for recurrent ovarian cancer. J Transl Med. 2012;10:1–10. 22863016 10.1186/1479-5876-10-157 PMC3439340

[234] Tchou J, Zhao Y, Levine BL, Zhang PJ, Davis MM, Melenhorst JJ, et al. Safety and efficacy of intratumoral injections of chimeric antigen receptor (CAR) T cells in metastatic breast cancer. Cancer Immunol Res. 2017;5(12):1152–61. 29109077 10.1158/2326-6066.CIR-17-0189 PMC5712264

[235] Bagley SJ, Desai AS, Linette GP, June CH, O’Rourke DM. CAR T-cell therapy for glioblastoma: recent clinical advances and future challenges. Neuro Oncol. 2018;20(11):1429–38. 29509936 10.1093/neuonc/noy032 PMC6176794

[236] Brown CE, Alizadeh D, Starr R, Weng L, Wagner JR, Naranjo A, et al. Regression of glioblastoma after chimeric antigen receptor T-cell therapy. N Engl J Med. 2016;375(26):2561–9. 28029927 10.1056/NEJMoa1610497 PMC5390684

[237] O’Rourke DM, Nasrallah MP, Desai A, Melenhorst JJ, Mansfield K, Morrissette JJ, et al. A single dose of peripherally infused EGFRvIII-directed CAR T cells mediates antigen loss and induces adaptive resistance in patients with recurrent glioblastoma. Science translational medicine. 2017;9(399):eaaa0984. 10.1126/scitranslmed.aaa0984 PMC5762203 28724573

[238] Ahmed N, Brawley V, Hegde M, Bielamowicz K, Kalra M, Landi D, et al. HER2-specific chimeric antigen receptor–modified virus-specific T cells for progressive glioblastoma: a phase 1 dose-escalation trial. JAMA Oncol. 2017;3(8):1094–101. 28426845 10.1001/jamaoncol.2017.0184 PMC5747970

[239] Depil S, Duchateau P, Grupp S, Mufti G, Poirot L. ‘Off-the-shelf’allogeneic CAR T cells: development and challenges. Nat Rev Drug Discovery. 2020;19(3):185–99. 31900462 10.1038/s41573-019-0051-2

[240] McCreedy BJ, Senyukov VV, Nguyen KT. Off the shelf T cell therapies for hematologic malignancies. Best Pract Res Clin Haematol. 2018;31(2):166–75. 29909917 10.1016/j.beha.2018.03.001

[241] Nitsche A, Zhang M, Clauss T, Siegert W, Brune K, Pahl A. Cytokine profiles of cord and adult blood leukocytes: differences in expression are due to differences in expression and activation of transcription factors. BMC Immunol. 2007;8:1–12. 17764543 10.1186/1471-2172-8-18 PMC2018703

[242] Gutman J, Ross K, Smith C, Myint H, Lee C, Salit R, et al. Chronic graft versus host disease burden and late transplant complications are lower following adult double cord blood versus matched unrelated donor peripheral blood transplantation. Bone Marrow Transplant. 2016;51(12):1588–93. 27400068 10.1038/bmt.2016.186

[243] Li D, Li X, Liao L, Li N. Unrelated cord blood transplantation versus haploidentical transplantation in adult and pediatric patients with hematological malignancies-a meta-analysis and systematic review. American Journal of Blood Research. 2020;10(1):1. 32206440 PMC7076284

[244] Sharma P, Purev E, Haverkos B, Pollyea DA, Cherry E, Kamdar M, et al. Adult cord blood transplant results in comparable overall survival and improved GRFS vs matched related transplant. Blood Adv. 2020;4(10):2227–35. 32442301 10.1182/bloodadvances.2020001554 PMC7252552

[245] Ma Q, Garber HR, Lu S, He H, Tallis E, Ding X, et al. A novel TCR-like CAR with specificity for PR1/HLA-A2 effectively targets myeloid leukemia in vitro when expressed in human adult peripheral blood and cord blood T cells. Cytotherapy. 2016;18(8):985–94. 27265873 10.1016/j.jcyt.2016.05.001 PMC4935572

[246] Papapetrou EP. Induced pluripotent stem cells, past and future. Science. 2016;353(6303):991–2. 27701103 10.1126/science.aai7626 PMC5234330

[247] Themeli M, Kloss CC, Ciriello G, Fedorov VD, Perna F, Gonen M, Sadelain M. Generation of tumor-targeted human T lymphocytes from induced pluripotent stem cells for cancer therapy. Nat Biotechnol. 2013;31(10):928–33. 23934177 10.1038/nbt.2678 PMC5722218

[248] Styczyński J, Tridello G, Koster L, Iacobelli S, van Biezen A, van der Werf S, et al. Death after hematopoietic stem cell transplantation: changes over calendar year time, infections and associated factors. Bone Marrow Transplant. 2020;55(1):126–36. 31455899 10.1038/s41409-019-0624-z PMC6957465

[249] Jagasia MH, Greinix HT, Arora M, Williams KM, Wolff D, Cowen EW, et al. National Institutes of Health consensus development project on criteria for clinical trials in chronic graft-versus-host disease: I. The 2014 Diagnosis and Staging Working Group report. Biology of Blood and Marrow Transplantation. 2015;21(3):389–401. e1. 10.1016/j.bbmt.2014.12.001 PMC4329079 25529383

[250] Abdelhakim H, Abdel-Azim H, Saad A. Role of αβ T cell depletion in prevention of graft versus host disease. Biomedicines. 2017;5(3):35. 28672883 10.3390/biomedicines5030035 PMC5618293

[251] Withers B, Blyth E, Clancy LE, Yong A, Fraser C, Burgess J, et al. Long-term control of recurrent or refractory viral infections after allogeneic HSCT with third-party virus-specific T cells. Blood Adv. 2017;1(24):2193–205. 29296867 10.1182/bloodadvances.2017010223 PMC5737128

[252] Tzannou I, Papadopoulou A, Naik S, Leung K, Martinez CA, Ramos CA, et al. Off-the-shelf virus-specific T cells to treat BK virus, human herpesvirus 6, cytomegalovirus, Epstein-Barr virus, and adenovirus infections after allogeneic hematopoietic stem-cell transplantation. J Clin Oncol. 2017;35(31):3547. 28783452 10.1200/JCO.2017.73.0655 PMC5662844

[253] D’Orsogna LJ, Roelen DL, Doxiadis II, Claas FH. Alloreactivity from human viral specific memory T-cells. Transpl Immunol. 2010;23(4):149–55. 20600900 10.1016/j.trim.2010.06.008

[254] Cruz CRY, Micklethwaite KP, Savoldo B, Ramos CA, Lam S, Ku S, et al. Infusion of donor-derived CD19-redirected virus-specific T cells for B-cell malignancies relapsed after allogeneic stem cell transplant: a phase 1 study. Blood, The Journal of the American Society of Hematology. 2013;122(17):2965–73. 10.1182/blood-2013-06-506741 PMC3811171 24030379

[255] Münz C. Redirecting T cells against Epstein-Barr Virus infection and associated oncogenesis. Cells. 2020;9(6):1400. 32512847 10.3390/cells9061400 PMC7349826

[256] Okamoto S, Mineno J, Ikeda H, Fujiwara H, Yasukawa M, Shiku H, Kato I. Improved expression and reactivity of transduced tumor-specific TCRs in human lymphocytes by specific silencing of endogenous TCR. Can Res. 2009;69(23):9003–11. 10.1158/0008-5472.CAN-09-1450 19903853

[257] Provasi E, Genovese P, Lombardo A, Magnani Z, Liu P-Q, Reik A, et al. Editing T cell specificity towards leukemia by zinc finger nucleases and lentiviral gene transfer. Nat Med. 2012;18(5):807–15. 22466705 10.1038/nm.2700 PMC5019824

[258] Sommer C, Boldajipour B, Kuo TC, Bentley T, Sutton J, Chen A, et al. Preclinical evaluation of allogeneic CAR T cells targeting BCMA for the treatment of multiple myeloma. Mol Ther. 2019;27(6):1126–38. 31005597 10.1016/j.ymthe.2019.04.001 PMC6554542

[259] Osborn MJ, Webber BR, Knipping F, Lonetree C-l, Tennis N, DeFeo AP, et al. Evaluation of TCR gene editing achieved by TALENs, CRISPR/Cas9, and megaTAL nucleases. Molecular Therapy. 2016;24(3):570–81. 10.1038/mt.2015.197 PMC4786913 26502778

[260] Georgiadis C, Preece R, Nickolay L, Etuk A, Petrova A, Ladon D, et al. Long terminal repeat CRISPR-CAR-coupled “universal” T cells mediate potent anti-leukemic effects. Mol Ther. 2018;26(5):1215–27. 29605708 10.1016/j.ymthe.2018.02.025 PMC5993944

[261] Wiebking V, Lee CM, Mostrel N, Lahiri P, Bak R, Bao G, et al. Genome editing of donor-derived T cells to generate allogeneic chimeric antigen receptor-modified T cells: optimizing αβ T-cell-depleted haploidentical hematopoietic stem cell transplantation. Haematologica. 2021;106(3):847. 32241852 10.3324/haematol.2019.233882 PMC7928014

[262] Ren J, Liu X, Fang C, Jiang S, June CH, Zhao Y. Multiplex genome editing to generate universal CAR T cells resistant to PD1 inhibition. Clin Cancer Res. 2017;23(9):2255–66. 27815355 10.1158/1078-0432.CCR-16-1300 PMC5413401

[263] Bothmer A, Gareau KW, Abdulkerim HS, Buquicchio F, Cohen L, Viswanathan R, et al. Detection and modulation of DNA translocations during multi-gene genome editing in T cells. The CRISPR journal. 2020;3(3):177–87. 32584143 10.1089/crispr.2019.0074

[264] Hale M, Lee B, Honaker Y, Leung W-H, Grier AE, Jacobs HM, et al. Homology-directed recombination for enhanced engineering of chimeric antigen receptor T cells. Molecular Therapy-Methods & Clinical Development. 2017;4:192–203. 28345004 10.1016/j.omtm.2016.12.008 PMC5363294

[265] Schober K, Müller TR, Gökmen F, Grassmann S, Effenberger M, Poltorak M, et al. Orthotopic replacement of T-cell receptor α-and β-chains with preservation of near-physiological T-cell function. Nature biomedical engineering. 2019;3(12):974–84. 31182835 10.1038/s41551-019-0409-0

[266] Petty AJ, Heyman B, Yang Y. Chimeric antigen receptor cell therapy: overcoming obstacles to battle cancer. Cancers. 2020;12(4):842. 32244520 10.3390/cancers12040842 PMC7226583

[267] Saetersmoen ML, Hammer Q, Valamehr B, Kaufman DS, Malmberg K-J, editors. Off-the-shelf cell therapy with induced pluripotent stem cell-derived natural killer cells. Seminars in immunopathology; 2019: Springer. 10.1007/s00281-018-0721-x 30361801

[268] Zeng J, Tang SY, Wang S. Derivation of mimetic γδ T cells endowed with cancer recognition receptors from reprogrammed γδ T cell. PLoS ONE. 2019;14(5): e0216815. 31071196 10.1371/journal.pone.0216815 PMC6508724

[269] Xu X, Huang W, Heczey A, Liu D, Guo L, Wood M, et al. NKT cells coexpressing a GD2-specific chimeric antigen receptor and IL15 show enhanced in vivo persistence and antitumor activity against neuroblastoma. Clin Cancer Res. 2019;25(23):7126–38. 31484667 10.1158/1078-0432.CCR-19-0421 PMC6891170

[270] Yazdanifar M, Barbarito G, Bertaina A, Airoldi I. γδ T cells: the ideal tool for cancer immunotherapy. Cells. 2020;9(5):1305. 32456316 10.3390/cells9051305 PMC7290982

[271] Capsomidis A, Benthall G, Van Acker HH, Fisher J, Kramer AM, Abeln Z, et al. Chimeric antigen receptor-engineered human gamma delta T cells: enhanced cytotoxicity with retention of cross presentation. Mol Ther. 2018;26(2):354–65. 29310916 10.1016/j.ymthe.2017.12.001 PMC5835118

[272] Anwer F, Shaukat A-A, Zahid U, Husnain M, McBride A, Persky D, et al. Donor origin CAR T cells: graft versus malignancy effect without GVHD, a systematic review. Immunotherapy. 2017;9(2):123–30. 28128714 10.2217/imt-2016-0127 PMC5827793

[273] Fraietta JA, Lacey SF, Orlando EJ, Pruteanu-Malinici I, Gohil M, Lundh S, et al. Determinants of response and resistance to CD19 chimeric antigen receptor (CAR) T cell therapy of chronic lymphocytic leukemia. Nat Med. 2018;24(5):563–71. 29713085 10.1038/s41591-018-0010-1 PMC6117613

[274] Louis CU, Savoldo B, Dotti G, Pule M, Yvon E, Myers GD, et al. Antitumor activity and long-term fate of chimeric antigen receptor–positive T cells in patients with neuroblastoma. Blood, The Journal of the American Society of Hematology. 2011;118(23):6050–6. 10.1182/blood-2011-05-354449 PMC3234664 21984804

[275] Benjamin R, Graham C, Yallop D, Jozwik A, Ciocarlie O, Jain N, et al. Preliminary data on safety, cellular kinetics and anti-leukemic activity of UCART19, an allogeneic anti-CD19 CAR T-cell product, in a pool of adult and pediatric patients with high-risk CD19+ relapsed/refractory B-cell acute lymphoblastic leukemia. Blood. 2018;132:896.

[276] Valton J, Guyot V, Marechal A, Filhol J-M, Juillerat A, Duclert A, et al. A multidrug-resistant engineered CAR T cell for allogeneic combination immunotherapy. Mol Ther. 2015;23(9):1507–18. 26061646 10.1038/mt.2015.104 PMC4817890

[277] Poirot L, Philip B, Schiffer-Mannioui C, Le Clerre D, Chion-Sotinel I, Derniame S, et al. Multiplex genome-edited T-cell manufacturing platform for “off-the-shelf” adoptive T-cell immunotherapies. Can Res. 2015;75(18):3853–64. 10.1158/0008-5472.CAN-14-3321 26183927

[278] Zhao W, Lei A, Tian L, Wang X, Correia C, Weiskittel T, et al. Strategies for genetically engineering hypoimmunogenic universal pluripotent stem cells. Iscience. 2020;23(6). 10.1016/j.isci.2020.101162 PMC7270609 32502965

[279] Revenfeld ALS, Steffensen R, Pugholm LH, Jørgensen MM, Stensballe A, Varming K. Presence of HLA-Dr Molecules and HLA-DRB 1 Mrna in Circulating CD 4+ T Cells. Scand J Immunol. 2016;84(4):211–21. 27417521 10.1111/sji.12462

[280] Krawczyk M, Peyraud N, Rybtsova N, Masternak K, Bucher P, Barras E, Reith W. Long distance control of MHC class II expression by multiple distal enhancers regulated by regulatory factor X complex and CIITA. J Immunol. 2004;173(10):6200–10. 15528357 10.4049/jimmunol.173.10.6200

[281] Kagoya Y, Guo T, Yeung B, Saso K, Anczurowski M, Wang C-H, et al. Genetic ablation of HLA class I, class II, and the T-cell receptor enables allogeneic T cells to be used for adoptive T-cell therapy. Cancer Immunol Res. 2020;8(7):926–36. 32321775 10.1158/2326-6066.CIR-18-0508

[282] de Rham C, Calderin Sollet Z, Burkhard P, Villard J. Natural killer cell alloreactivity against human induced pluripotent stem cells and their neuronal derivatives into dopaminergic neurons. Stem cells and development. 2020;29(13):853–62. 32245345 10.1089/scd.2019.0201

[283] Baier C, Fino A, Sanchez C, Farnault L, Rihet P, Kahn-Perlès B, Costello RT. Natural killer cells modulation in hematological malignancies. Front Immunol. 2013;4:459. 24391641 10.3389/fimmu.2013.00459 PMC3867693

[284] Gornalusse GG, Hirata RK, Funk SE, Riolobos L, Lopes VS, Manske G, et al. HLA-E-expressing pluripotent stem cells escape allogeneic responses and lysis by NK cells. Nat Biotechnol. 2017;35(8):765–72. 28504668 10.1038/nbt.3860 PMC5548598

[285] Carosella ED, Rouas-Freiss N, Tronik-Le Roux D, Moreau P, LeMaoult J. HLA-G: an immune checkpoint molecule. Adv Immunol. 2015;127:33–144. 26073983 10.1016/bs.ai.2015.04.001

[286] Zheng Y, Ma X, Su D, Zhang Y, Yu L, Jiang F, et al. The roles of Siglec7 and Siglec9 on natural killer cells in virus infection and tumour progression. Journal of immunology research. 2020;2020. 10.1155/2020/6243819 PMC7165337 32322597

[287] Mo F, Watanabe N, McKenna MK, Hicks MJ, Srinivasan M, Gomes-Silva D, et al. Engineered off-the-shelf therapeutic T cells resist host immune rejection. Nat Biotechnol. 2021;39(1):56–63. 32661440 10.1038/s41587-020-0601-5 PMC7854790

[288] Majzner RG, Mackall CL. Tumor antigen escape from CAR T-cell therapy. Cancer Discov. 2018;8(10):1219–26. 30135176 10.1158/2159-8290.CD-18-0442

[289] Ruella M, Maus MV. Catch me if you can: leukemia escape after CD19-directed T cell immunotherapies. Comput Struct Biotechnol J. 2016;14:357–62. 27761200 10.1016/j.csbj.2016.09.003 PMC5061074

[290] Anurathapan U, Chan RC, Hindi HF, Mucharla R, Bajgain P, Hayes BC, et al. Kinetics of tumor destruction by chimeric antigen receptor-modified T cells. Mol Ther. 2014;22(3):623–33. 24213558 10.1038/mt.2013.262 PMC3945803

[291] Minutolo NG, Hollander EE, Powell DJ Jr. The emergence of universal immune receptor T cell therapy for cancer. Front Oncol. 2019;9:176. 30984613 10.3389/fonc.2019.00176 PMC6448045

[292] Hughes-Parry HE, Cross RS, Jenkins MR. The evolving protein engineering in the design of chimeric antigen receptor T cells. Int J Mol Sci. 2019;21(1):204. 31892219 10.3390/ijms21010204 PMC6981602

[293] Cho JH, Collins JJ, Wong WW. Universal chimeric antigen receptors for multiplexed and logical control of T cell responses. Cell. 2018;173(6):1426–38. e11. 10.1016/j.cell.2018.03.038 PMC5984158 29706540

[294] Cartellieri M, Feldmann A, Koristka S, Arndt C, Loff S, Ehninger Av, et al. Switching CAR T cells on and off: a novel modular platform for retargeting of T cells to AML blasts. Blood cancer journal. 2016;6(8):e458-e. 10.1038/bcj.2016.61 PMC5022178 27518241

[295] Bachmann M. The UniCAR system: a modular CAR T cell approach to improve the safety of CAR T cells. Immunol Lett. 2019;211:13–22. 31091431 10.1016/j.imlet.2019.05.003

[296] Feldmann A, Arndt C, Koristka S, Berndt N, Bergmann R, Bachmann MP. Conventional CARs versus modular CARs. Cancer Immunol Immunother. 2019;68:1713–9. 31542798 10.1007/s00262-019-02399-5 PMC6805801

[297] Minutolo NG, Sharma P, Poussin M, Shaw LC, Brown DP, Hollander EE, et al. Quantitative control of gene-engineered T-cell activity through the covalent attachment of targeting ligands to a universal immune receptor. J Am Chem Soc. 2020;142(14):6554–68. 32191035 10.1021/jacs.9b11622 PMC7306176

[298] Liu D, Zhao J, Song Y. Engineering switchable and programmable universal CARs for CAR T therapy. J Hematol Oncol. 2019;12:1–9. 31272471 10.1186/s13045-019-0763-0 PMC6610960

[299] Liu T-C, Galanis E, Kirn D. Clinical trial results with oncolytic virotherapy: a century of promise, a decade of progress. Nat Clin Pract Oncol. 2007;4(2):101–17. 17259931 10.1038/ncponc0736

[300] Taqi A, Abdurrahman M, Yakubu A, Fleming A. Regression of Hodgkin’s disease after measles. The Lancet. 1981;317(8229):1112. 10.1016/s0140-6736(81)92286-8 6112483

[301] Southam CM. Division of microbiology: present status of oncolytic virus studies. Transactions of the New York Academy of Sciences. 1960;22(8 Series II):657–73. 10.1111/j.2164-0947.1960.tb00739.x 13833074

[302] Msaouel P, Dispenzieri A, Galanis E. Clinical testing of engineered oncolytic measles virus strains in the treatment of cancer: an overview. Curr Opin Mol Ther. 2009;11(1):43. 19169959 PMC2717625

[303] Wollmann G, Ozduman K, Van Den Pol AN. Oncolytic virus therapy of glioblastoma multiforme–concepts and candidates. Cancer journal (Sudbury, Mass). 2012;18(1):69. 22290260 10.1097/PPO.0b013e31824671c9 PMC3632333

[304] Chiocca EA, Abbed KM, Tatter S, Louis DN, Hochberg FH, Barker F, et al. A phase I open-label, dose-escalation, multi-institutional trial of injection with an E1B-Attenuated adenovirus, ONYX-015, into the peritumoral region of recurrent malignant gliomas, in the adjuvant setting. Mol Ther. 2004;10(5):958–66. 15509513 10.1016/j.ymthe.2004.07.021

[305] Markert JM, Liechty PG, Wang W, Gaston S, Braz E, Karrasch M, et al. Phase Ib trial of mutant herpes simplex virus G207 inoculated pre-and post-tumor resection for recurrent GBM. Mol Ther. 2009;17(1):199–207. 18957964 10.1038/mt.2008.228 PMC2834981

[306] Markert JM, Razdan SN, Kuo H-C, Cantor A, Knoll A, Karrasch M, et al. A phase 1 trial of oncolytic HSV-1, G207, given in combination with radiation for recurrent GBM demonstrates safety and radiographic responses. Mol Ther. 2014;22(5):1048–55. 24572293 10.1038/mt.2014.22 PMC4015243

[307] Allen C, Opyrchal M, Aderca I, Schroeder MA, Sarkaria JN, Domingo E, et al. Oncolytic measles virus strains have significant antitumor activity against glioma stem cells. Gene Ther. 2013;20(4):444–9. 22914495 10.1038/gt.2012.62 PMC3509233

[308] Kicielinski KP, Chiocca EA, John SY, Gill GM, Coffey M, Markert JM. Phase 1 clinical trial of intratumoral reovirus infusion for the treatment of recurrent malignant gliomas in adults. Mol Ther. 2014;22(5):1056–62. 24553100 10.1038/mt.2014.21 PMC4015229

[309] Gromeier M, Lachmann S, Rosenfeld MR, Gutin PH, Wimmer E. Intergeneric poliovirus recombinants for the treatment of malignant glioma. Proc Natl Acad Sci. 2000;97(12):6803–8. 10841575 10.1073/pnas.97.12.6803 PMC18745

[310] Freeman AI, Zakay-Rones Z, Gomori JM, Linetsky E, Rasooly L, Greenbaum E, et al. Phase I/II trial of intravenous NDV-HUJ oncolytic virus in recurrent glioblastoma multiforme. Mol Ther. 2006;13(1):221–8. 16257582 10.1016/j.ymthe.2005.08.016

[311] Russell SJ, Peng K-W, Bell JC. Oncolytic virotherapy. Nat Biotechnol. 2012;30(7):658–70. 22781695 10.1038/nbt.2287 PMC3888062

[312] Martuza RL, Malick A, Markert JM, Ruffner KL, Coen DM. Experimental therapy of human glioma by means of a genetically engineered virus mutant. Science. 1991;252(5007):854–6. 1851332 10.1126/science.1851332

[313] Wheeler LA, Manzanera AG, Bell SD, Cavaliere R, McGregor JM, Grecula JC, et al. Phase II multicenter study of gene-mediated cytotoxic immunotherapy as adjuvant to surgical resection for newly diagnosed malignant glioma. Neuro Oncol. 2016;18(8):1137–45. 26843484 10.1093/neuonc/now002 PMC4933478

[314] Chiocca EA, Aguilar LK, Bell SD, Kaur B, Hardcastle J, Cavaliere R, et al. Phase IB study of gene-mediated cytotoxic immunotherapy adjuvant to up-front surgery and intensive timing radiation for malignant glioma. J Clin Oncol. 2011;29(27):3611. 21844505 10.1200/JCO.2011.35.5222 PMC3179270

[315] Westphal M, Ylä-Herttuala S, Martin J, Warnke P, Menei P, Eckland D, et al. Adenovirus-mediated gene therapy with sitimagene ceradenovec followed by intravenous ganciclovir for patients with operable high-grade glioma (ASPECT): a randomised, open-label, phase 3 trial. Lancet Oncol. 2013;14(9):823–33. 23850491 10.1016/S1470-2045(13)70274-2

[316] Twumasi-Boateng K, Pettigrew JL, Kwok YE, Bell JC, Nelson BH. Oncolytic viruses as engineering platforms for combination immunotherapy. Nat Rev Cancer. 2018;18(7):419–32. 29695749 10.1038/s41568-018-0009-4

[317] Zwernik SD, Adams BH, Raymond DA, Warner CM, Kassam AB, Rovin RA, Akhtar P. AXL receptor is required for Zika virus strain MR-766 infection in human glioblastoma cell lines. Molecular Therapy-Oncolytics. 2021;23:447–57. 34901388 10.1016/j.omto.2021.11.001 PMC8626839

[318] Chen Q, Wu J, Ye Q, Ma F, Zhu Q, Wu Y, et al. Treatment of human glioblastoma with a live attenuated Zika virus vaccine candidate. MBio. 2018;9(5):e01683-e1718. 30228241 10.1128/mBio.01683-18 PMC6143740

[319] Zhang Z, Rong L, Li Y-P. Flaviviridae viruses and oxidative stress: implications for viral pathogenesis. Oxidative medicine and cellular longevity. 2019;2019. 10.1155/2019/1409582 PMC6720866 31531178

[320] Saxena M, van der Burg SH, Melief CJ, Bhardwaj N. Therapeutic cancer vaccines. Nat Rev Cancer. 2021;21(6):360–78. 33907315 10.1038/s41568-021-00346-0

[321] Hu Z, Ott PA, Wu CJ. Towards personalized, tumour-specific, therapeutic vaccines for cancer. Nat Rev Immunol. 2018;18(3):168–82. 29226910 10.1038/nri.2017.131 PMC6508552

[322] Weller M, Kaulich K, Hentschel B, Felsberg J, Gramatzki D, Pietsch T, et al. Assessment and prognostic significance of the epidermal growth factor receptor vIII mutation in glioblastoma patients treated with concurrent and adjuvant temozolomide radiochemotherapy. Int J Cancer. 2014;134(10):2437–47. 24614983 10.1002/ijc.28576

[323] Schuster J, Lai RK, Recht LD, Reardon DA, Paleologos NA, Groves MD, et al. A phase II, multicenter trial of rindopepimut (CDX-110) in newly diagnosed glioblastoma: the ACT III study. Neuro Oncol. 2015;17(6):854–61. 25586468 10.1093/neuonc/nou348 PMC4483122

[324] Sampson JH, Aldape KD, Archer GE, Coan A, Desjardins A, Friedman AH, et al. Greater chemotherapy-induced lymphopenia enhances tumor-specific immune responses that eliminate EGFRvIII-expressing tumor cells in patients with glioblastoma. Neuro Oncol. 2011;13(3):324–33. 21149254 10.1093/neuonc/noq157 PMC3064599

[325] Weller M, Butowski N, Tran DD, Recht LD, Lim M, Hirte H, et al. Rindopepimut with temozolomide for patients with newly diagnosed, EGFRvIII-expressing glioblastoma (ACT IV): a randomised, double-blind, international phase 3 trial. Lancet Oncol. 2017;18(10):1373–85. 28844499 10.1016/S1470-2045(17)30517-X

[326] Reardon DA, Desjardins A, Vredenburgh JJ, O’Rourke DM, Tran DD, Fink KL, et al. Rindopepimut with bevacizumab for patients with relapsed EGFRvIII-expressing glioblastoma (ReACT): results of a double-blind randomized phase II trial. Clin Cancer Res. 2020;26(7):1586–94. 32034072 10.1158/1078-0432.CCR-18-1140

[327] Phuphanich S, Wheeler CJ, Rudnick JD, Mazer M, Wang H, Nuno MA, et al. Phase I trial of a multi-epitope-pulsed dendritic cell vaccine for patients with newly diagnosed glioblastoma. Cancer Immunol Immunother. 2013;62:125–35. 22847020 10.1007/s00262-012-1319-0 PMC3541928

[328] Prins RM, Soto H, Konkankit V, Odesa SK, Eskin A, Yong WH, et al. Gene expression profile correlates with T-cell infiltration and relative survival in glioblastoma patients vaccinated with dendritic cell immunotherapy. Clin Cancer Res. 2011;17(6):1603–15. 21135147 10.1158/1078-0432.CCR-10-2563 PMC3071163

[329] Liau LM, Ashkan K, Tran DD, Campian JL, Trusheim JE, Cobbs CS, et al. First results on survival from a large Phase 3 clinical trial of an autologous dendritic cell vaccine in newly diagnosed glioblastoma. J Transl Med. 2018;16(1):1–9. 29843811 10.1186/s12967-018-1507-6 PMC5975654

[330] Blass E, Ott PA. Advances in the development of personalized neoantigen-based therapeutic cancer vaccines. Nat Rev Clin Oncol. 2021;18(4):215–29. 33473220 10.1038/s41571-020-00460-2 PMC7816749

[331] Keskin DB, Anandappa AJ, Sun J, Tirosh I, Mathewson ND, Li S, et al. Neoantigen vaccine generates intratumoral T cell responses in phase Ib glioblastoma trial. Nature. 2019;565(7738):234–9. 30568305 10.1038/s41586-018-0792-9 PMC6546179

[332] Tan AC, Ashley DM, López GY, Malinzak M, Friedman HS, Khasraw M. Management of glioblastoma: state of the art and future directions. CA: a cancer journal for clinicians. 2020;70(4):299–312. 10.3322/caac.21613 32478924

[333] Idbaih A, Canney M, Belin L, Desseaux C, Vignot A, Bouchoux G, et al. Safety and feasibility of repeated and transient blood–brain barrier disruption by pulsed ultrasound in patients with recurrent glioblastoma. Clin Cancer Res. 2019;25(13):3793–801. 30890548 10.1158/1078-0432.CCR-18-3643

[334] Castro BA, Aghi MK. Bevacizumab for glioblastoma: current indications, surgical implications, and future directions. Neurosurg Focus. 2014;37(6):E9. 25581938 10.3171/2014.9.focus14516 PMC4839778

[335] Chinot OL, Wick W, Mason W, Henriksson R, Saran F, Nishikawa R, et al. Bevacizumab plus radiotherapy–temozolomide for newly diagnosed glioblastoma. N Engl J Med. 2014;370(8):709–22. 24552318 10.1056/NEJMoa1308345

[336] Gilbert MR, Dignam JJ, Armstrong TS, Wefel JS, Blumenthal DT, Vogelbaum MA, et al. A randomized trial of bevacizumab for newly diagnosed glioblastoma. N Engl J Med. 2014;370(8):699–708. 24552317 10.1056/NEJMoa1308573 PMC4201043

[337] Carpentier A, Canney M, Vignot A, Reina V, Beccaria K, Horodyckid C, et al. Clinical trial of blood-brain barrier disruption by pulsed ultrasound. Science translational medicine. 2016;8(343):343re2-re2. 10.1126/scitranslmed.aaf6086 27306666

[338] Timbie KF, Mead BP, Price RJ. Drug and gene delivery across the blood–brain barrier with focused ultrasound. J Control Release. 2015;219:61–75. 26362698 10.1016/j.jconrel.2015.08.059 PMC4656107

[339] Beccaria K, Canney M, Goldwirt L, Fernandez C, Piquet J, Perier M-C, et al. Ultrasound-induced opening of the blood-brain barrier to enhance temozolomide and irinotecan delivery: an experimental study in rabbits. J Neurosurg. 2016;124(6):1602–10. 26566207 10.3171/2015.4.JNS142893

[340] Sun T, Zhang Y, Power C, Alexander PM, Sutton JT, Aryal M, et al. Closed-loop control of targeted ultrasound drug delivery across the blood–brain/tumor barriers in a rat glioma model. Proc Natl Acad Sci. 2017;114(48):E10281–90. 29133392 10.1073/pnas.1713328114 PMC5715774

[341] Newman WC, Amankulor NA. Focused ultrasound enhances central nervous system delivery of bevacizumab for malignant glioma treatment. Neurosurgery. 2016;79(6):N12. 27861405 10.1227/NEU.0000000000001451

[342] Liu H-L, Hsu P-H, Lin C-Y, Huang C-W, Chai W-Y, Chu P-C, et al. Focused ultrasound enhances central nervous system delivery of bevacizumab for malignant glioma treatment. Radiology. 2016;281(1):99–108. 27192459 10.1148/radiol.2016152444

[343] Kobus T, Zervantonakis IK, Zhang Y, McDannold NJ. Growth inhibition in a brain metastasis model by antibody delivery using focused ultrasound-mediated blood-brain barrier disruption. J Control Release. 2016;238:281–8. 27496633 10.1016/j.jconrel.2016.08.001 PMC5014601

[344] Dréan A, Lemaire N, Bouchoux G, Goldwirt L, Canney M, Goli L, et al. Temporary blood–brain barrier disruption by low intensity pulsed ultrasound increases carboplatin delivery and efficacy in preclinical models of glioblastoma. J Neurooncol. 2019;144:33–41. 31197598 10.1007/s11060-019-03204-0

[345] Meng Y, Pople CB, Suppiah S, Llinas M, Huang Y, Sahgal A, et al. MR-guided focused ultrasound liquid biopsy enriches circulating biomarkers in patients with brain tumors. Neuro Oncol. 2021;23(10):1789–97. 33693781 10.1093/neuonc/noab057 PMC8485448

[346] Mainprize T, Lipsman N, Huang Y, Meng Y, Bethune A, Ironside S, et al. Blood-brain barrier opening in primary brain tumors with non-invasive MR-guided focused ultrasound: a clinical safety and feasibility study. Sci Rep. 2019;9(1):321. 30674905 10.1038/s41598-018-36340-0 PMC6344541

[347] Sobhani N, Samadani AA. Implications of photodynamic cancer therapy: an overview of PDT mechanisms basically and practically. J Egypt Natl Canc Inst. 2021;33:1–13. 34778919 10.1186/s43046-021-00093-1 PMC13316895

[348] Tirrò E, Massimino M, Romano C, Martorana F, Pennisi MS, Stella S, et al. Prognostic and therapeutic roles of the insulin growth factor system in glioblastoma. Front Oncol. 2021;10: 612385. 33604294 10.3389/fonc.2020.612385 PMC7885861

[349] Andrews DW, Judy KD, Scott CB, Garcia S, Harshyne LA, Kenyon L, et al. Phase Ib clinical trial of IGV-001 for patients with newly diagnosed glioblastoma. Clin Cancer Res. 2021;27(7):1912–22. 33500356 10.1158/1078-0432.CCR-20-3805

[350] Reardon DA, Brem S, Desai AS, Bagley SJ, Kurz SC, De La Fuente MI, et al. INO-5401 and INO-9012 delivered intramuscularly (IM) with electroporation (EP) in combination with cemiplimab (REGN2810) in newly diagnosed glioblastoma (GBM): Interim results. American Society of Clinical Oncology; 2020.

[351] Lee Y, Koh J, Kim S-I, Won JK, Park C-K, Choi SH, Park S-H. The frequency and prognostic effect of TERT promoter mutation in diffuse gliomas. Acta Neuropathol Commun. 2017;5:1–11. 28851427 10.1186/s40478-017-0465-1 PMC5574236

[352] Lee S, Kambhampati M, Yadavilli S, Gordish-Dressman H, Santi M, Cruz CR, et al. Differential expression of Wilms’ tumor protein in diffuse intrinsic pontine glioma. J Neuropathol Exp Neurol. 2019;78(5):380–8. 30990879 10.1093/jnen/nlz021 PMC6467196

[353] Nakahara Y, Okamoto H, Mineta T, Tabuchi K. Expression of the Wilms’ tumor gene product WT1 in glioblastomas and medulloblastomas. Brain Tumor Pathol. 2004;21:113–6. 15696971 10.1007/BF02482185

[354] Holzgreve A, Biczok A, Ruf VC, Liesche-Starnecker F, Steiger K, Kirchner MA, et al. PSMA expression in glioblastoma as a basis for theranostic approaches: a retrospective, correlational panel study including immunohistochemistry, clinical parameters and PET imaging. Front Oncol. 2021;11: 646387. 33859946 10.3389/fonc.2021.646387 PMC8042319

[355] Chakraborty C, Sharma AR, Bhattacharya M, Lee S-S. From COVID-19 to cancer mRNA vaccines: moving from bench to clinic in the vaccine landscape. Front Immunol. 2021;12: 679344. 34305909 10.3389/fimmu.2021.679344 PMC8293291

[356] Chan HY, Choi J, Jackson C, Lim M. Combination immunotherapy strategies for glioblastoma. J Neurooncol. 2021;151:375–91. 33611705 10.1007/s11060-020-03481-0

[357] Zhu S, Zhang T, Zheng L, Liu H, Song W, Liu D, et al. Combination strategies to maximize the benefits of cancer immunotherapy. J Hematol Oncol. 2021;14(1):156. 34579759 10.1186/s13045-021-01164-5 PMC8475356

[358] Speranza M-C, Passaro C, Ricklefs F, Kasai K, Klein SR, Nakashima H, et al. Preclinical investigation of combined gene-mediated cytotoxic immunotherapy and immune checkpoint blockade in glioblastoma. Neuro Oncol. 2018;20(2):225–35. 29016938 10.1093/neuonc/nox139 PMC5777502

[359] Saha D, Martuza RL, Rabkin SD. Macrophage polarization contributes to glioblastoma eradication by combination immunovirotherapy and immune checkpoint blockade. Cancer cell. 2017;32(2):253–67. e5. 10.1016/j.ccell.2017.07.006 PMC5568814 28810147

[360] Finocchiaro G, Gentner B, Farina F, Capotondo A, Eoli M, Anghileri E, et al. A phase I-IIa study of genetically modified Tie-2 expressing monocytes in patients with glioblastoma multiforme (TEM-GBM Study). Wolters Kluwer Health; 2021.

[361] Hu S, Hui Z, Duan J, Garrido C, Xie T, Ye X-Y. Discovery of small-molecule ATR inhibitors for potential cancer treatment: a patent review from 2014 to present. Expert Opin Ther Pat. 2022;32(4):401–21. 35001778 10.1080/13543776.2022.2027911

[362] Cheng Y, Tian H. Current development status of MEK inhibitors. Molecules. 2017;22(10):1551. 28954413 10.3390/molecules22101551 PMC6151813

[363] Song Y, Bi Z, Liu Y, Qin F, Wei Y, Wei X. Targeting RAS–RAF–MEK–ERK signaling pathway in human cancer: Current status in clinical trials. Genes & Diseases. 2023;10(1):76–88. 37013062 10.1016/j.gendis.2022.05.006 PMC10066287

[364] Schreck KC, Allen AN, Wang J, Pratilas CA. Combination MEK and mTOR inhibitor therapy is active in models of glioblastoma. Neuro-Oncology Advances. 2020;2(1):vdaa138. 10.1093/noajnl/vdaa138 PMC7668446 33235998

[365] Li Y, Dong Q, Cui Y. Synergistic inhibition of MEK and reciprocal feedback networks for targeted intervention in malignancy. Cancer Biol Med. 2019;16(3):415. 31565475 10.20892/j.issn.2095-3941.2019.0137 PMC6743629

[366] Selvasaravanan KD, Wiederspohn N, Hadzalic A, Strobel H, Payer C, Schuster A, et al. The limitations of targeting MEK signalling in Glioblastoma therapy. Sci Rep. 2020;10(1):7401. 32366879 10.1038/s41598-020-64289-6 PMC7198577

[367] Gao M, Yang J, Gong H, Lin Y, Liu J. Trametinib inhibits the growth and aerobic glycolysis of glioma cells by targeting the PKM2/c-Myc axis. Front Pharmacol. 2021;12: 760055. 34744739 10.3389/fphar.2021.760055 PMC8566436

[368] Shannon S, Jia D, Entersz I, Beelen P, Yu M, Carcione C, et al. Inhibition of glioblastoma dispersal by the MEK inhibitor PD0325901. BMC Cancer. 2017;17(1):1–11. 28187762 10.1186/s12885-017-3107-x PMC5303286

[369] Colardo M, Segatto M, Di Bartolomeo S. Targeting RTK-PI3K-mTOR axis in gliomas: an update. Int J Mol Sci. 2021;22(9):4899. 34063168 10.3390/ijms22094899 PMC8124221

[370] Brennan CW, Verhaak RG, McKenna A, Campos B, Noushmehr H, Salama SR, et al. The somatic genomic landscape of glioblastoma. Cell. 2014;157(3):753. 10.1016/j.cell.2013.09.034 PMC3910500 24120142

[371] Ji M, Zhang Z, Lin S, Wang C, Jin J, Xue N, et al. The PI3K inhibitor XH30 enhances response to temozolomide in drug-resistant glioblastoma via the noncanonical Hedgehog signaling pathway. Front Pharmacol. 2021;12: 749242. 34899305 10.3389/fphar.2021.749242 PMC8662317

[372] Yao W, Gong H, Mei H, Shi L, Yu J, Hu Y. Taxifolin targets pi3k and mtor and inhibits glioblastoma multiforme. Journal of Oncology. 2021;2021. 10.1155/2021/5560915 PMC8403040 34462635

[373] Li X, Wu C, Chen N, Gu H, Yen A, Cao L, et al. PI3K/Akt/mTOR signaling pathway and targeted therapy for glioblastoma. Oncotarget. 2016;7(22):33440. 26967052 10.18632/oncotarget.7961 PMC5078108

[374] Zhao H-f, Wang J, Shao W, Wu C-p, Chen Z-p, To S-sT, Li W-p. Recent advances in the use of PI3K inhibitors for glioblastoma multiforme: current preclinical and clinical development. Molecular cancer. 2017;16:1–16. 10.1186/s12943-017-0670-3 PMC5463420 28592260

[375] Farooq M, Khan AW, Kim MS, Choi S. The role of fibroblast growth factor (FGF) signaling in tissue repair and regeneration. Cells. 2021;10(11):3242. 34831463 10.3390/cells10113242 PMC8622657

[376] Jimenez-Pascual A, A. Siebzehnrubl F. Fibroblast growth factor receptor functions in glioblastoma. Cells. 2019;8(7):715. 10.3390/cells8070715 PMC6678715 31337028

[377] Roskoski R Jr. The role of fibroblast growth factor receptor (FGFR) protein-tyrosine kinase inhibitors in the treatment of cancers including those of the urinary bladder. Pharmacol Res. 2020;151: 104567. 31770593 10.1016/j.phrs.2019.104567

[378] Georgescu M-M, Islam MZ, Li Y, Traylor J, Nanda A. Novel targetable FGFR2 and FGFR3 alterations in glioblastoma associate with aggressive phenotype and distinct gene expression programs. Acta Neuropathol Commun. 2021;9(1):1–17. 33853673 10.1186/s40478-021-01170-1 PMC8048363

[379] Turner N, Pearson A, Sharpe R, Lambros M, Geyer F, Lopez-Garcia MA, et al. FGFR1 amplification drives endocrine therapy resistance and is a therapeutic target in breast cancer. Can Res. 2010;70(5):2085–94. 10.1158/0008-5472.CAN-09-3746 PMC2832818 20179196

[380] Fernanda Amary M, Ye H, Berisha F, Khatri B, Forbes G, Lehovsky K, et al. Fibroblastic growth factor receptor 1 amplification in osteosarcoma is associated with poor response to neo-adjuvant chemotherapy. Cancer Med. 2014;3(4):980–7. 24861215 10.1002/cam4.268 PMC4303166

[381] Sayal KK, Higgins GS, Hammond EM. Uncovering the influence of the FGFR1 pathway on glioblastoma radiosensitivity. Annals of Translational Medicine. 2016;4(24). 10.21037/atm.2016.11.65 PMC5233509 28149899

[382] Hierro C, Rodon J, Tabernero J, editors. Fibroblast growth factor (FGF) receptor/FGF inhibitors: novel targets and strategies for optimization of response of solid tumors. Seminars in oncology; 2015: Elsevier. 10.1053/j.seminoncol.2015.09.027 26615127

[383] Ohashi R, Matsuda Y, Ishiwata T, Naito Z. Downregulation of fibroblast growth factor receptor 2 and its isoforms correlates with a high proliferation rate and poor prognosis in high-grade glioma. Oncol Rep. 2014;32(3):1163–9. 24968791 10.3892/or.2014.3283

[384] Hoang-Minh LB, Siebzehnrubl FA, Yang C, Suzuki-Hatano S, Dajac K, Loche T, et al. Infiltrative and drug-resistant slow-cycling cells support metabolic heterogeneity in glioblastoma. EMBO J. 2018;37(23): e98772. 30322894 10.15252/embj.201798772 PMC6276884

[385] Gabler L, Jaunecker CN, Katz S, van Schoonhoven S, Englinger B, Pirker C, et al. Fibroblast growth factor receptor 4 promotes glioblastoma progression: A central role of integrin-mediated cell invasiveness. Acta Neuropathol Commun. 2022;10(1):65. 35484633 10.1186/s40478-022-01363-2 PMC9052585

[386] Ardizzone A, Scuderi SA, Giuffrida D, Colarossi C, Puglisi C, Campolo M, et al. Role of fibroblast growth factors receptors (FGFRs) in brain tumors, focus on astrocytoma and glioblastoma. Cancers. 2020;12(12):3825. 33352931 10.3390/cancers12123825 PMC7766440

[387] Kurzyk A. Angiogenesis-possibilities, problems and perspectives. Postepy Biochem. 2015;61(1):25–34. 26281351

[388] Loureiro LVM, Neder L, Callegaro-Filho D, de Oliveira KL, Stavale JN, Malheiros SMF. The immunohistochemical landscape of the VEGF family and its receptors in glioblastomas. Surgical and Experimental Pathology. 2020;3(1):1–8.

[389] Tatla AS, Justin AW, Watts C, Markaki AE. A vascularized tumoroid model for human glioblastoma angiogenesis. Sci Rep. 2021;11(1):19550. 34599235 10.1038/s41598-021-98911-y PMC8486855

[390] Mahase S, Rattenni RN, Wesseling P, Leenders W, Baldotto C, Jain R, Zagzag D. Hypoxia-mediated mechanisms associated with antiangiogenic treatment resistance in glioblastomas. Am J Pathol. 2017;187(5):940–53. 28284719 10.1016/j.ajpath.2017.01.010 PMC5417003

[391] Guyon J, Chapouly C, Andrique L, Bikfalvi A, Daubon T. The normal and brain tumor vasculature: Morphological and functional characteristics and therapeutic targeting. Front Physiol. 2021;12:125. 10.3389/fphys.2021.622615 PMC7973205 33746770

[392] Hundsberger T, Reardon DA, Wen PY. Angiogenesis inhibitors in tackling recurrent glioblastoma. Expert Rev Anticancer Ther. 2017;17(6):507–15. 28438066 10.1080/14737140.2017.1322903

[393] Ahir BK, Engelhard HH, Lakka SS. Tumor development and angiogenesis in adult brain tumor: glioblastoma. Mol Neurobiol. 2020;57:2461–78. 32152825 10.1007/s12035-020-01892-8 PMC7170819

